# The Oxytetracycline Producer *Streptomyces lydicamycinicus* bja295 and Its Genomic Context

**DOI:** 10.3390/ijms27188403

**Published:** 2026-09-21

**Authors:** Mikhail Yu Dobryakov, Julia A. Buyuklyan, Mikhail V. Biryukov

**Affiliations:** 1 Research Center for Translational Medicine, Sirius University of Science and Technology, Sochi 354340, Russia; dobryakov.my@gmail.com; 2 Department of Biology, Lomonosov Moscow State University, Moscow 119991, Russia

**Keywords:** comparative genomics, type II polyketide synthase, gene cluster distribution, carriage frequency, vertical inheritance, gene loss, tetracycline resistance genes, genome mining, *oxyO*

## Abstract

Oxytetracycline (OTC) production is classically attributed to *Streptomyces rimosus*, and the distribution of its biosynthetic gene cluster (BGC) across the genus is poorly understood. We describe *Streptomyces lydicamycinicus* bja295 as an OTC producer, the first demonstrated for this species, and the distribution of the BGC across the genus. OTC was identified by mass spectrometry, and the titre reached 200 mg/L on ISP2, the most productive medium tested. A maximum-likelihood tree of 138 single-copy core proteins places the strain within *S. lydicamycinicus* as sister to its type strain at 99.36% ANI; the genome is a linear chromosome of 8,072,839 bp (71.2% GC). Its intact locus comprises 24 genes and lacks an ortholog of *oxyO*, a gene of the canonical *S. rimosus* cluster of long-debated function; since the strain produces OTC, *oxyO* is not required for the trait in this background. Of 13,198 *Streptomyces* genomes screened, 137 carry the BGC (1.04%): every other assembly of *S. lydicamycinicus*; the single assemblies of *S. albofaciens*, *S. viridifaciens* and *S. aureofaciens*; and subclades within *S. rimosus*, *S. varsoviensis*, *S. platensis* and *S. halstedii*. In non-carriers, the syntenic site holds unrelated 15–27 kb blocks or nothing, while *otrA* and *otrB* persist without the biosynthetic core, marking where the cluster once stood rather than a capacity to produce. Carriage is thus structured by lineage rather than scattered across the genus, and carriage, structural integrity and demonstrated production are three separate statements about a genome.

## 1. Introduction

The search for new antibiotics and the introduction of new antimicrobial agents into practice is one of the most important tasks of modern microbiology [1,2]. Actinomycetes of the genus *Streptomyces* remain the principal source of naturally occurring antibiotics, and the continuing renewal of screening approaches yields not only new compounds but also new biology for molecules that are already known [3,4].

A limitation of conventional dereplication pipelines—in which an activity is matched against databases of known compounds by mass spectrometry features—is that not every biologically active compound is efficiently identified by standard chemico-analytical methods. A considerable proportion of the active compounds therefore remains unrecognised [5]. The development of next-generation sequencing (NGS) methods has made ever more genomes available and has created a demand for approaches that allow the productive potential of strains to be assessed—both by examining individual genomes for known biosynthetic gene clusters (BGCs) and sequence patterns [6], and, by means of pangenomic analysis, by detecting potentially new and promising genetic constructs, judging their integrity, and devising ways to activate them through OSMAC (One Strain Many Compounds) or genome editing [7,8]. The volume of this material has itself become a problem: the first release of the Natural Products Discovery Center (NPDC) comprises 8490 sequenced strains and adds some 7000 gene cluster families that were not represented among RefSeq Actinobacteria [9], while only about 3% of the natural products encoded in sequenced bacterial genomes have so far been chemically characterised [10], and the majority of clusters remain transcriptionally silent under laboratory conditions and needed the genome-guided OSMAC optimisation or genome editing [4]. How best to exploit this abundance remains an open question.

In the course of a mechanism-based screening campaign employing the Dualrep2 system [11,12,13,14,15], we isolated strain *Streptomyces* sp. bja295, a producer of an inhibitor of bacterial translation; the compound is identified in Section 2.2.

Tetracycline antibiotics, the class to which that compound belongs, are one of the oldest groups, natural and synthetic alike, and still find active application in medicine and in veterinary practice, despite their moderate toxicity and the spread of resistance to them [16,17]. The surge of resistance is thought to be due in part to the excessive use of tetracyclines in animal husbandry, which follows from a side effect of these substances—the growth-promoting acceleration of anabolism in farm animals [18].

OTC is a natural antibiotic of the tetracycline series whose industrial production is carried out exclusively by microbiological synthesis (submerged fermentation). Global consumption of tetracyclines amounts to tens of thousands of tonnes per year—an estimated 33,305 t in food-producing animals in 2020 alone [19]. The largest producer and consumer of antibiotics in the world is China, with an annual output of about 210,000 t across all classes of antibiotic, of which some 48% is used in agriculture [20]; the main production capacities for OTC are concentrated in Asia.

Global consumption is dominated by the veterinary and agricultural sectors, which account for 80–90% of total output. Tetracyclines rank first among all classes of antimicrobial agents used in global animal husbandry, and OTC is the most widely applied individual antibiotic in aquaculture [19,21]. In addition, the substance is officially registered—in particular, by the United States Environmental Protection Agency (EPA)—as a bactericidal agent for the protection of plants against phytopathogens, in particular, against *Erwinia amylovora*, the causative agent of fire blight of apple and pear [22,23].

The natural first-generation tetracyclines are conventionally partitioned among producer species: chlortetracycline is synthesised by *Streptomyces aureofaciens* (now *Kitasatospora aureofaciens*), OTC by *S. rimosus*, whereas unmodified tetracycline is obtained industrially, for the most part, by dechlorination of chlortetracycline [17,24]. The principal industrial producer is the actinomycete *S. rimosus*, cultivated in submerged batch culture on a starch-based complex medium supplemented with soy flour and corn steep liquor [25]. The capacity for its synthesis has also been recorded in individual strains of *S. alboflavus* [26], *S. varsoviensis* [27] and *S. platensis* [28]. Full chemical synthesis of OTC is economically impractical because of its complex spatial configuration and its numerous chiral centres; related drugs, such as doxycycline and minocycline, are meanwhile obtained by semisynthetic modification of the natural substrate [16].

The genetics and biochemistry of OTC biosynthesis in *S. rimosus* have been described in detail, which makes it a suitable candidate for comparative pangenomic studies. A minimal type II polyketide synthase (OxyA/OxyB/OxyC) assembles the decaketide backbone, the amidotransferase OxyD supplies the malonamyl starter unit that gives tetracyclines their C2 carboxamide, and a set of tailoring enzymes (OxyD–OxyT) introduces the oxidative, cyclising and transferase modifications [24,29,30]. Regulation is provided by the cluster-situated SARP-type activator OtcR [31] and by the LAL-type (LuxR-family) regulator OtcG [32], while self-protection of the producer rests on the ribosomal protection protein OtrA and the MFS-family efflux pump OtrB, which flank the biosynthetic genes [33,34,35].

Strain bja295 was assigned to the species *Streptomyces lydicamycinicus* on the basis of whole-genome analysis. The species was described in 2020 from a strain isolated from marine water and was characterised primarily as a producer of the linear polyketide antibiotic lydicamycin and its congeners; the OTC BGC was reported in the article describing the type species, but production of the antibiotic itself was not mentioned, and in the available literature, the species is credited with lydicamycin only [36]. Although all published genomes of representatives of this species carry the OTC BGC, for none of them are there publications on the ability to produce this compound. *S. lydicamycinicus* is phylogenetically distant from *S. rimosus*, which is traditionally regarded as the OTC producer. The nomenclature of the clade to which the species belongs is further complicated by a history of reclassification: *S. hygroscopicus* subsp. *glebosus* and *S. libani* subsp. *rufus* have been reduced to later heterotypic synonyms of *S. platensis* [37], and *S. libani* to a later heterotypic synonym of *S. nigrescens* [38], so that a number of genomes in public databases are still deposited under obsolete names—which makes it difficult to collate data on OTC producers within the clade correctly. A literature search showed that OTC production has been reported for diverse representatives of the taxon *Streptomyces*, and, as generally understood, the ability to produce OTC is a strain-specific rather than a species-specific trait [39,40]. Within the framework of the present work, we therefore decided to carry out a more detailed pangenomic analysis of the distribution and the variability of the OTC BGC among representatives of the genus *Streptomyces*. Genome mining has recently widened the chemical picture of the family—a survey of some 600,000 bacterial genomes retrieved 82 representative tetracycline BGCs and yielded three new structural groups of tetracycline-type metabolites [41]—but that work was directed at new chemistry rather than at the fate of the canonical locus.

How a biosynthetic locus is distributed across a genus is not a bookkeeping question: the pattern separates dissemination by horizontal transfer, which leaves a mosaic at the level of strains, from descent with loss, which leaves a distribution structured by lineage, and it therefore determines whether the presence of a cluster in a genome can be predicted from taxonomy at all [42]. Three gaps follow from the above. Production of OTC has never been demonstrated for *S. lydicamycinicus*; although the cluster is present in its genomes, the distribution of the cluster across the genus has not been quantified, and the fate of the locus outside *S. rimosus*—whether it moves between lineages or descends within them—is unknown. The present work addresses these three questions on one strain and on the genus as a whole, and tests the hypothesis that the locus is inherited vertically, with losses prevailing over gains, rather than disseminated by frequent recent transfer. To the best of our knowledge, strain bja295 is the first representative of *S. lydicamycinicus* for which the production of OTC has been demonstrated experimentally. We report the mass-spectrometric identification of the synthesised molecule, a phylogenetic analysis of the strain from whole-genome sequencing data, an annotation of the BGC including the resistance and regulatory genes that are absent from the existing reference MIBiG entry BGC0000254, and a comparative-genomic reconstruction of the evolution of the cluster within the clade. Strain-specific genetic elements served as an additional tool for reconstructing the relationships among strains of the species. Methodologically, the contribution of this work lies in the framework into which the single strain is placed: a locus definition derived from co-inheritance rather than from cluster-prediction boundaries, an explicit separation of carriage, integrity and production, and null models against which the agreement between the cluster and the genome tree is judged.

## 2. Results and Discussion

### 2.1. Characterisation of S. lydicamycinicus bja295

**Figure 1:**
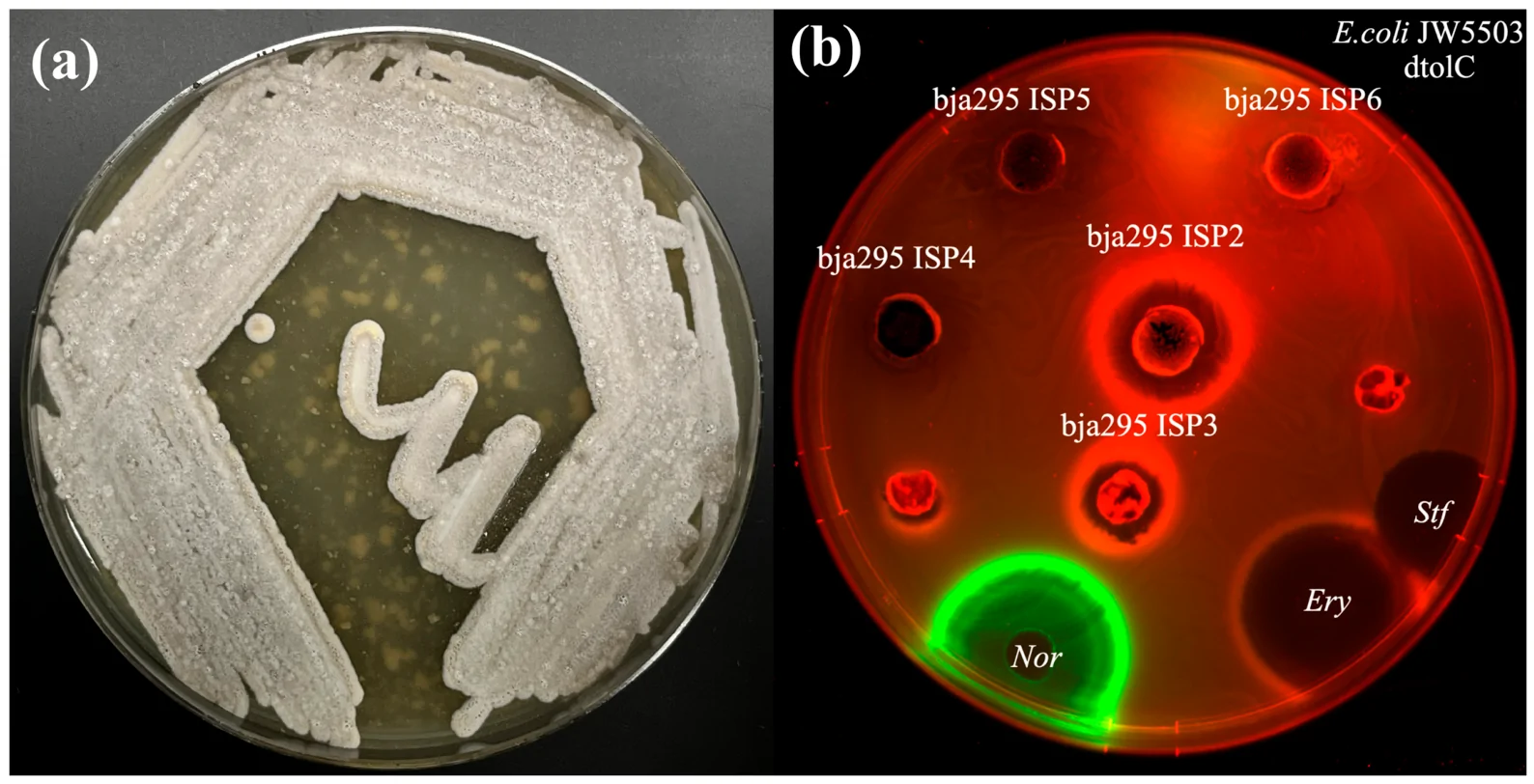
(**a**) Growth of the *S. lydicamycinicus* bja295 strain on ISP3 medium. (**b**) Activity of *S. lydicamycinicus* bja295 CFS (cell-free supernatant) against the test strain *E. coli* JW5503 Δ*tolC* pDualrep2. Positive controls were erythromycin (0.05 µg), which induces expression of Katushka2S; norfloxacin (1 µg), which induces expression of TurboRFP; and 1 µg of streptothricin. For clarity, the Katushka2S signal (inhibition of translation) is rendered in red and the TurboRFP signal (DNA damage) in green by the ChemiDoc MP v6.0.1 software.

Strain bja295 exhibits characteristics typical of the species *S. lydicamycinicus*: it is an aerobic, Gram-positive, non-motile actinobacterium. The strain showed vigorous growth on ISP3 media (Figure 1a). The substrate mycelium displayed colour variations ranging from white to silver grey on the tested media, whereas the aerial mycelium was consistently white. Melanoid pigment production was not observed on any of the ISP media recommended for taxonomic characterisation.

Carbon-source utilisation was assessed on ISP9 basal medium supplemented with individual substrates; the strain assimilated every substrate of the panel, the complete list being given in Table S1. Assimilation was recorded as growth or no growth, and the three replicates agreed on every substrate. Hydrolysis of starch, of cellulose and of casein was tested on the corresponding solid media and was negative in all three cases, in all three replicates: the strain assimilates the mono- and oligosaccharides of the panel without showing extracellular degradation of these polymers. Such uniformly positive assimilation indicates the broad catabolic versatility characteristic of soil streptomycetes and places no constraint on the choice of production medium; accordingly, the differences in metabolite output observed between media reflect regulation of biosynthesis rather than a limitation in substrate uptake.

The antibiotic susceptibility profile was determined by disc diffusion on ISP3 (Table S2); each disc was assayed in three independent biological replicates, and the table gives the mean, the standard deviation and the three individual readings. Strain bja295 was susceptible to inhibitors of protein synthesis acting on both ribosomal subunits. Three substances of the panel caused no or minimal inhibition zones. Streptomycin produced no detectable zone, and neither did streptothricin F applied at 500 µg; tigecycline, the only tetracycline in the panel and the most potent member of the class in clinical use, gave the smallest zone among all ribosome-targeting discs (16 mm) compared to 24–40 mm for the macrolides, the lincosamide and the aminoglycosides. The absence of a streptomycin zone is accounted for by the aminoglycoside phosphotransferase APH(3″)-Ia encoded in the genome, although no streptomycin biosynthetic cluster is predicted. The phenotypic profile places bja295 within the variation described for *S. lydicamycinicus* and provides the physiological reference against which the genomic analysis below is interpreted.

### 2.2. Identification of the Active Compound

During the primary screening of *S. lydicamycinicus* bja295 grown on various solid ISP media, antagonistic activity was detected against the test strain Δ*tolC*. This strain carries a disruption in the efflux pump system, resulting in compromised cellular defence mechanisms and increased susceptibility to antibacterial compounds. Notably, the metabolite produced by *S. lydicamycinicus* bja295 on ISP2 and ISP3 media induced the expression of Katushka2S, which is associated with the inhibition of bacterial translation (Figure 1b), whereas the TurboRFP reporter of the DNA damage remained silent. The mechanism of action indicated at this stage therefore guided the entire subsequent fractionation scheme: at each step, the fractions were followed by induction of Katushka2S rather than by inhibition zones alone.

CFS with detected activity was clarified, acidified and subjected to solid-phase extraction (SPE). Translation-inhibiting activity eluted in the 10% and 20% acetonitrile fractions; these were lyophilised, redissolved in the corresponding acetonitrile concentration to a 2.5-fold enrichment, and separated by reversed-phase HPLC. Activity was confined to a single peak with a retention time of 11.59 min and an absorption maximum at 356 nm.

The active HPLC fraction was analysed by LC–MS. The compound gave an intense [M + H]^+^ ion at *m*/*z* 461.1517, indistinguishable from that of an authentic oxytetracycline standard analysed in the same sequence (*m*/*z* 461.1515); both lie some 8 ppm below the value calculated for protonated C_22_H_24_N_2_O_9_ (461.1555), a systematic offset of the calibration of that run, which affects sample and standard alike. Fragmentation followed the course expected for a tetracycline, with the loss of water and the subsequent loss of ammonia (*m*/*z* 443.1407 and 426.1132). In the negative mode, no ion attributable to the deprotonated compound was recorded. Taken together with the absorption maximum at 356 nm, a search of the NPAtlas, Dictionary of Natural Products and PubChem databases revealed a compound with the molecular formula C_22_H_24_N_2_O_9_, matching OTC (Figure S1). The same formula is shared by 11a-hydroxytetracycline. The identification of OTC was confirmed by co-chromatography with an authentic OTC standard, whose retention time and UV spectrum coincided with those of the active fraction.

An independent, target-based confirmation of the tetracycline nature of the antibiotic was obtained with an isogenic pair constructed earlier in this laboratory: *E. coli* JW5503 Δ*tolC* and its derivative carrying a tetracycline-resistance determinant (Δ*tolC tetR*). The active fraction of bja295 inhibited the Δ*tolC* strain, but the Δ*tolC tetR* derivative was resistant to active compounds. In the same experiment, the fraction and authentic tetracycline gave a matching pattern of activity against *E. coli* K12, including comparable induction of the Katushka2S reporter. Quantification against a pharmacopoeial standard gave a titre of 200 mg/L on ISP2, the most productive of the media tested. The determination rests on a two-fold dilution series and is therefore semi-quantitative: it places the titre in a dilution step rather than measuring it to within a few per cent, and the value is given without a dispersion estimate for that reason. For scale, the wild-type *S. rimosus* ATCC 10970^T^ accumulates 70 mg/L of OTC in complex medium, whereas the industrially mutagenised hyperproducer HP126 reaches 4490 mg/L under the same conditions [25]. An unimproved strain grown without any optimisation of the fermentation therefore produces about three times the titre of the wild-type reference and some 20 times less than an industrial producer, which is the range expected of an intact but unengineered locus.

### 2.3. Genome Features and Phylogenomic Analysis

Whole-genome sequencing of strain bja295, followed by de novo genome assembly, yielded a linear chromosome of 8,072,839 bp with a G + C content of 71.2%, which is consistent with representatives of the genus *Streptomyces* [43]. Assessment of genome completeness using BUSCO v5.8.2 against the actinobacteria_class_odb10 lineage dataset (*n* = 356) revealed a completeness score of 100.0%, comprising 98.6% single-copy and 1.4% duplicated BUSCOs. The chromosome includes six ribosomal operons, an orphan 5S rRNA gene, 68 tRNA genes and one tmRNA gene, as predicted by Bakta, and the genome encodes 7094 coding sequences. The deposited record additionally contains a second, smaller replicon of 106,533 bp, so that the record measures 8,179,372 bp in total. The annotation was performed on the deposited record in full, so that the counts reported here are those of the record as deposited.

The terminal 21 kb of the chromosome record are duplicated within the assembly, and the last 6.9 kb carry almost no reads; the record was deposited without trimming, and all coordinates in this work refer to it.

**Figure 2:**
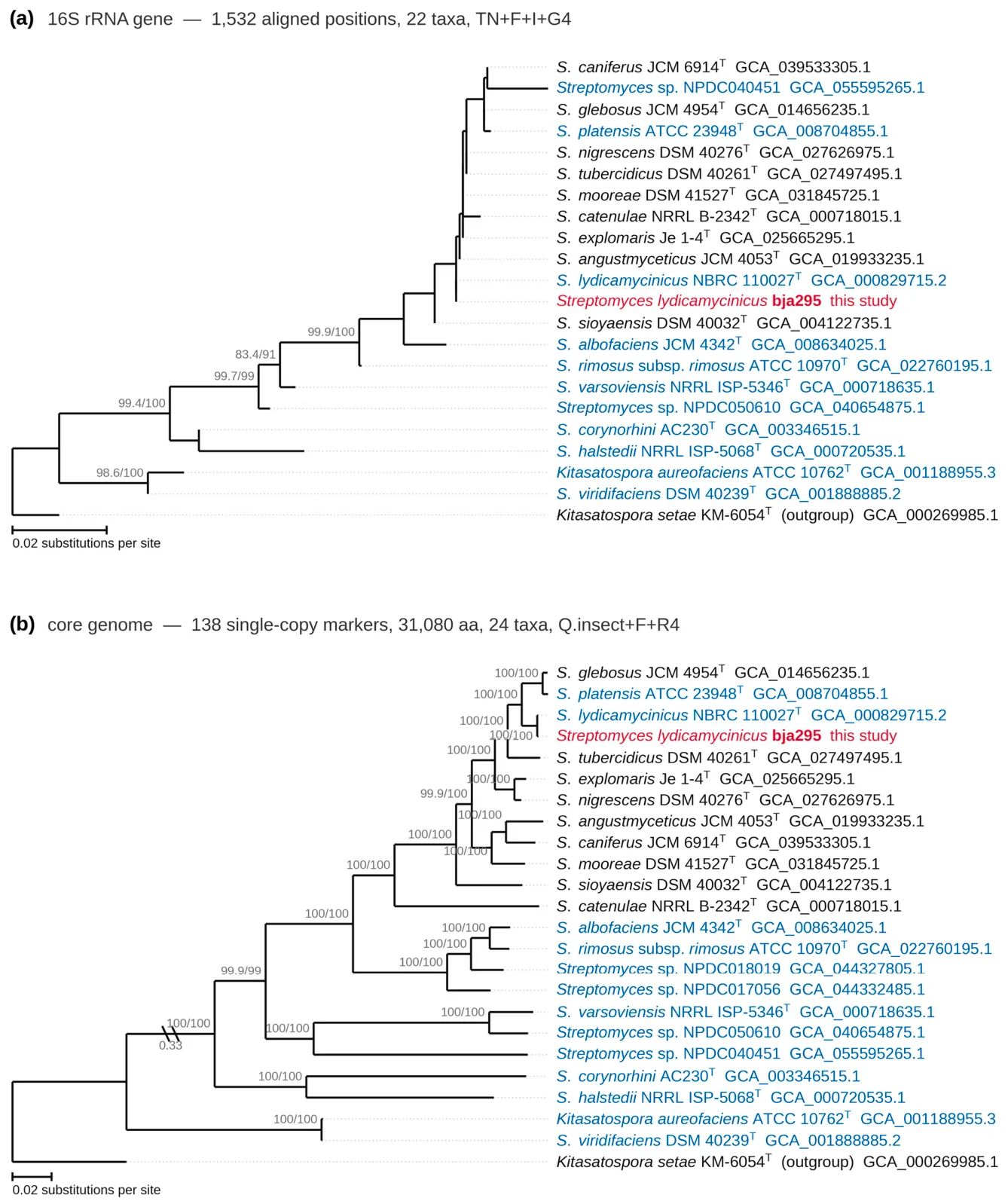
Phylogenetic position of strain bja295. (**a**) Maximum-likelihood tree of the 16S rRNA gene (1532 aligned positions, 22 taxa; model TN + F + I + G4). (**b**) Maximum-likelihood tree of a concatenate of 138 single-copy markers of the Actinobacteria set (31,080 amino acid positions, 24 taxa; model Q.insect + F + R4). Tip labels give the species and the strain, with the accession of the assembly; a superscript T marks a type strain. The strain described here is shown in red, species in which the oxytetracycline locus was detected in blue, and the remaining taxa in black. Both trees were inferred from the same genome panel in IQ-TREE v2.3.6; branch support is given as SH-aLRT/ultrafast bootstrap (1000 replicates) and is shown where both values are ≥70. GenBank accessions of the assemblies used are given next to the tip labels; the RefSeq counterparts of the same assemblies were the actual input. Strains NPDC018019 and NPDC017056 yielded no usable 16S rRNA gene sequence and are absent from (**a**). The panel comprises strain bja295, the type strains of all species carrying the oxytetracycline locus, one representative of each unnamed carrier lineage and the closest relatives of the strain; *Kitasatospora setae* KM-6054^T^ served as the outgroup. Type strains are marked with a superscript T; strain bja295 is shown in red and carrier species in blue. In (**b**), the branch subtending the *Streptomyces* clade is shortened for display and marked with a double slash; its actual length is 0.33 substitutions per site.

Maximum-likelihood analysis of the 16S rRNA gene and of a concatenate of 138 single-copy core proteins, over a panel comprising the type strains of all species that carry the OTC locus, one representative of every unnamed carrier lineage and the closest relatives of the strain, placed bja295 within the species *Streptomyces lydicamycinicus* as sister to its type strain with full support (Figure 2); the same placement follows from Genome BLAST Distance Phylogeny distances computed on the Type (Strain) Genome Server (Figure S2). The topology of the 16S tree agrees with the one published in the description of the species [36]: bja295 and the type strain NBRC 110027 form a pair inside the same group of close relatives, and the species is placed alongside *S. platensis* and *S. glebosus*. Accordingly, the strain is hereinafter designated as *S. lydicamycinicus* bja295. Average nucleotide identity computed with skani between bja295 and the genomes of the species panel ranged from 99.13% to 99.53%, the value against the type strain *S. lydicamycinicus* NBRC 110027^T^ being 99.36%; all of these exceed the 95% threshold generally accepted as the criterion for species delineation [44]. Digital DNA–DNA hybridisation values over a panel of 46 genomes of the complex are given in Table S3, and the matrix of the 1035 pairwise ANI comparisons from which the corresponding genomospecies threshold was derived is given in Table S4.

**Figure 3:**
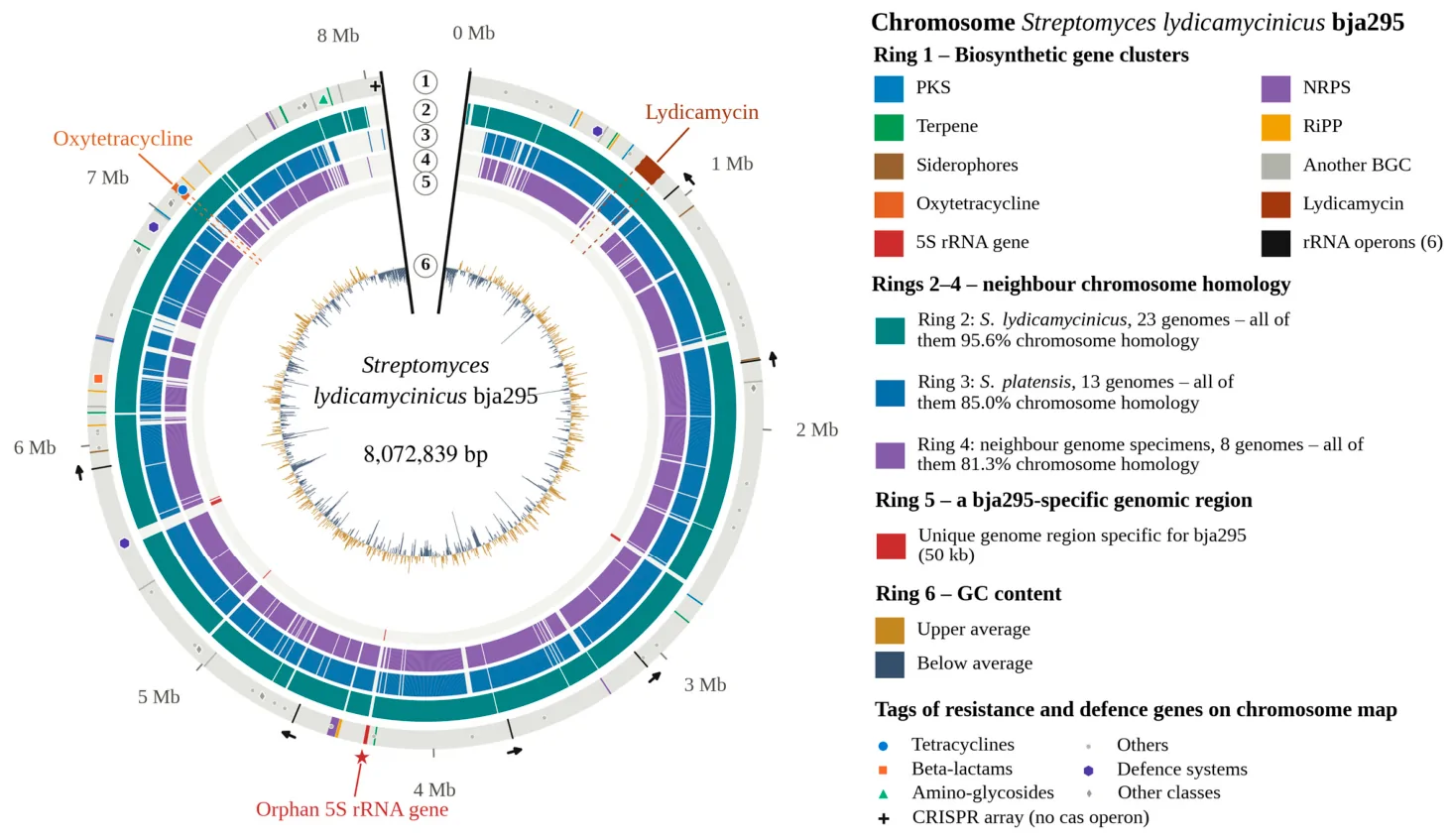
Map of chromosomal organisation and topology distribution of target metabolite biosynthetic gene clusters (BGCs), including oxytetracycline and lydicamycin. Ring 1, resistance and defence genes and the biosynthetic gene clusters predicted by antiSMASH, coloured by class; rings 2–4, homology to the chromosomes of the neighbour genome panels; ring 5, the bja295-specific region; ring 6, GC content relative to the genome average. The map is drawn for the chromosome alone; the second, smaller replicon of the deposited record is not represented.

Annotation of secondary metabolite biosynthesis gene clusters with antiSMASH [6] revealed 33 putative BGCs, two of which matched clusters encoding compounds with described antimicrobial activity. For the OTC region, KnownClusterBlast recovered hits to 19 proteins of the MIBiG entry BGC0000254 at 69–88% identity (median 77%); for the lydicamycin region, hits to 24 proteins of BGC0001477 at 89–100% identity (median 93%) (Table S5). The remaining BGCs were predicted to encode pigments, osmoprotectants, or clusters comprising only 30–60% of the genes confidently required for the biosynthesis of a complete secondary metabolite (Figure 3). The antimicrobial compounds predicted by this approach were considered primary candidates for the subsequent identification of the metabolites produced by the strain.

The inventory beyond these two clusters is dominated by classes whose products are either not antibacterial or not identifiable from sequence alone: five terpene regions, three siderophore regions, three butyrolactone regions, three RiPP-like regions, three lanthipeptide or lasso-peptide regions, and single regions for ectoine, hydrogen cyanide, an atropopeptide and a triceptide. Ten regions reach a MIBiG entry with more than four proteins hit, among them the spore pigment (10 proteins), dudomycin A (11), hopene (8), isorenieratene (7) and glycinocin A (7), whereas six regions return no MIBiG hit at all and correspond to no described compound. The two regions that can be tied to a product in this strain—lydicamycin and the OTC locus—are also the two best supported by homology, at 24 and 19 proteins, respectively; the unassigned regions remain untested and are the obvious material for further work on this strain.

A sliding window comparison of the bja295 chromosome with the 23 other genomes of the species examined in this work, using canonical 21-mers, shows that 95.6% of the chromosome belongs to the region shared by all 23 of them, while only 0.6% is strain-specific (Figure 3). Both of the species’ biosynthetic clusters are located in the shared region: all six windows covering the OTC locus and all 21 windows of the lydicamycin protocluster core are present in every genome of that panel. The bja295-specific blocks of the biosynthetic clusters, which relate to mobile elements and defence systems against foreign DNA, are entirely absent and have a lower GC content (61.1–66.8% compared with 71.2% across the chromosome).

In the deposited assembly, the OTC region occupies positions 7,078,336–7,150,890; these 72.6 kb are the antiSMASH region, which includes the flanking sequence, whereas the co-inherited 24-gene core of the locus spans 27.5 kb, from 7,109,763 to 7,137,267 (Section 2.6). The region lies 0.92 Mb from the nearest chromosome end and 1.2 Mb distal to the last ribosomal operon, that is, entirely within the terminal arm, as has also been described for *S. rimosus* [45]. The density of IS elements in the arms exceeds that in the central part of the chromosome by a factor of 24 (*p* = 1.4 × 10^−8^); the locus itself, however, does not differ from a randomly chosen arm window of the same length (*p* = 0.25), that is, it lacks the framing of mobile elements typical of recently transferred regions.

Having established bja295 as a producer of OTC that carries a complete and structurally intact locus, we use its genome in what follows as the reference point of the genus-wide analysis: to define the structural boundaries of the cluster from co-inheritance rather than from prediction boundaries (Section 2.4), to screen the genus for its presence (Section 2.5), and to reconstruct the history of the locus and of the chromosomal site that carries it (Section 2.6 and Section 2.7).

### 2.4. Structure of the Locus in Carrier Species

**Figure 4:**
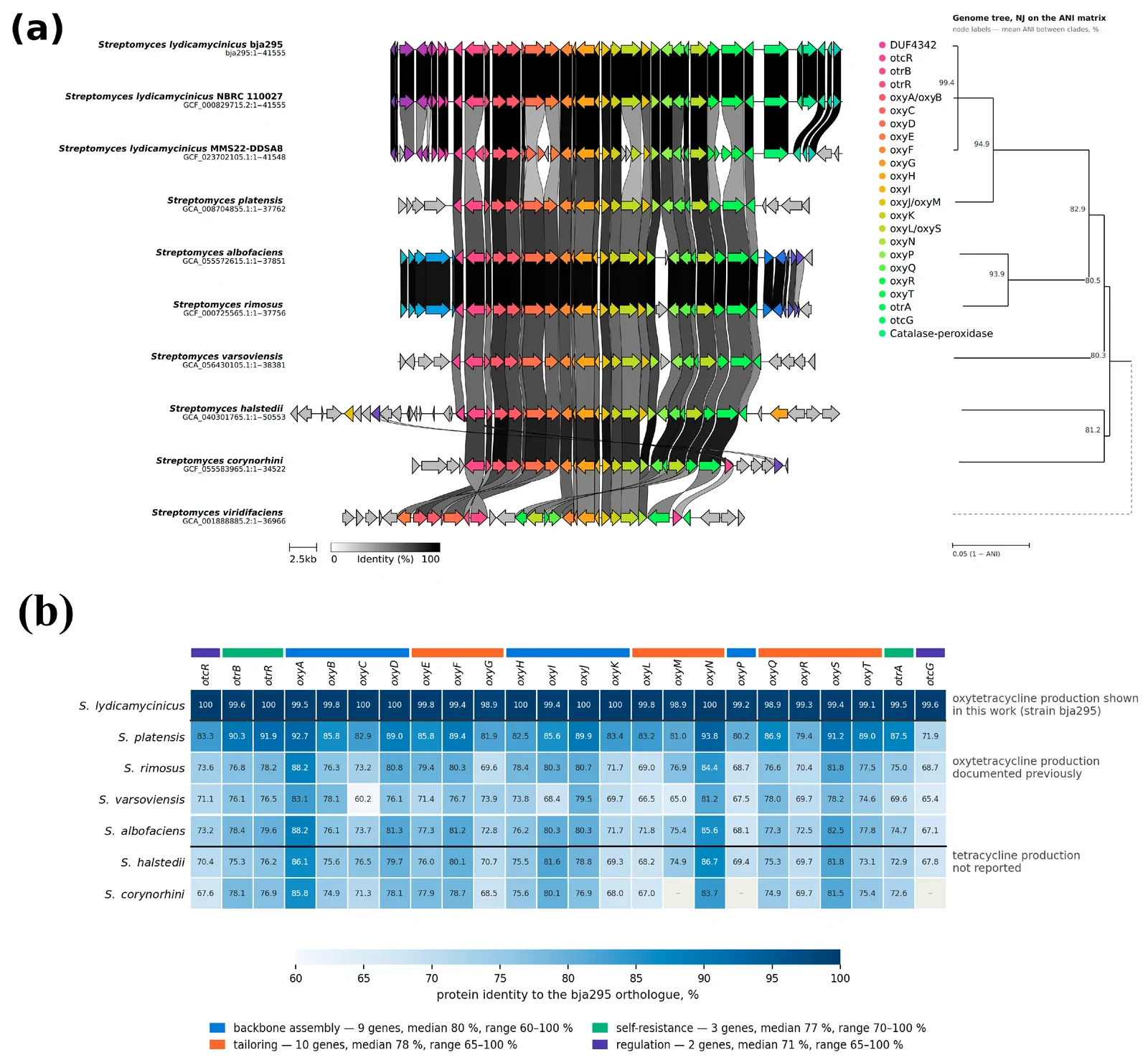
The oxytetracycline locus in carrier species. (**a**) Alignment of the locus with its flanking regions across ten genomes; rows are ordered according to the ANI tree shown on the right, with loci aligned against common genes; arrow colour denotes the homology group, and the annotations used were corrected for the merged oxyH–oxyG frames described in Section 2.4. Each row is labelled with the accession of the assembly and the coordinates of the region shown. The grey bands between the rows link genes of the same homology group and are shaded by their pairwise identity on the greyscale given below the panel (0–100%); the bar to its left marks 2.5 kb. The tree to the right of the panel is a neighbour-joining tree of the ANI matrix, on which the rows are ordered; the figures at its nodes are the mean ANI between the clades that the node joins, in per cent, and its scale bar is given in units of 1 − ANI. (**b**) Identity of the 24 proteins of the locus with the bja295 orthologues, with one representative from each carrier species; columns are arranged in the order of the genes within the locus. Identity is the amino acid identity of the best tblastn hit of each of the 24 bja295 locus proteins in the corresponding assembly, and a dash marks a protein for which no hit was recovered; the colour scale beneath the map runs from 60 to 100% identity. The annotations to the right of the map group the species by what is known of tetracycline production, and the key below it assigns each gene of the locus to one of four functional groups, giving for every group the number of genes it contains and the median and the range of their identity.

A comparison of the loci across 10 genomes (bja295, two further strains of *S. lydicamycinicus* and one representative of each remaining carrier species) shows that the core of the cluster is built identically: the same set of genes in the same order, with vertical homology ribbons that do not cross (Figure 4a). The same locus was reported in the description of the species, where it is designated t2pks-2 and assigned to oxytetracycline, its minimal polyketide synthase being listed as TPA0598_07_00590, TPA0598_07_00600 and TPA0598_07_00610 [36]; these three proteins correspond one-to-one to the ketosynthase, the chain-length factor and the acyl carrier protein of the core defined here, which occupy positions 7,113,336–7,114,613, 7,114,610–7,115,890 and 7,115,949–7,116,302 of the bja295 chromosome. That report established the presence of the cluster in the type strain; whether the locus is complete across the species, and whether the compound is produced, are the questions addressed here. Beyond the core, the similarity breaks off, and the flanking genes form separate, lineage-specific groups. The only crossing of ribbons within the core falls on *S. corynorhini*, which has lost *oxyM*, *oxyP* and *otcG*, with the retained *otcR* located on the opposite edge of the locus. Defective loci were revealed on the same material and in the same manner: an assembly passed the three-gene probe and entered the set of carriers, yet on the subsequent comparison of all 24 genes, it showed missing genes, an altered gene order or a mobile element inserted within the core. Thus, in the most distant carrier, the block occupies 34.8 kb instead of 25.6 kb, with an altered gene order and a median identity of 55.2%, while in *S. varsoviensis*, an IS110 element is inserted within the core alongside a pseudogenised methyltransferase. Tetracycline production has not been described for any of these strains.

One gene of the core is systematically missed by automatic annotation. In *S. albofaciens*, *S. rimosus*, *S. corynorhini* and two genomes deposited without a species name, oxyG lies in the same reading frame and on the same strand as the adjacent oxyH, with no stop codon and no intergenic gap between them, so gene callers merge the two into a single protein of 644 or 654 residues; in bja295 and in the other genomes of *S. lydicamycinicus* an insertion of about 53 bp carrying a stop codon separates the two frames, and the genes are called individually. The gene itself is present in every carrier: a protein search recovers it over its full length, at 88–100% identity to the entry of the reference cluster, in all of the loci examined. Figure 4a is drawn from annotations in which the merged frames were split accordingly, so that the panel agrees with the identities reported in Figure 4b; the discrepancy affected the annotation layer only and not the searches on which Table 1 and Table S5 are based.

The identity of the locus proteins to the bja295 orthologues forms a continuous gradient that follows the relatedness of the genomes: 99.6% in *S. lydicamycinicus*, 85.8% in *S. platensis*, 76.7% in *S. rimosus*, 73.6% in *S. varsoviensis* and 53.3% in the most distant carrier (Figure 4b); BiG-SCAPE distances [46] change in the same order, from 0.004 to 0.853. There are no discontinuities in the gradient, which corresponds to the divergence of a single ancestral locus rather than to the independent assembly of a similar set of functions in different lineages. Producer status is not predicted by this gradient: in *S. rimosus* ATCC 10970^T^, the median identity is 76.8%; in *S. varsoviensis*, for which production has been described previously [27], 73.8%; and in *S. albofaciens*, for which the locus has been reported but production has not [41], 76.8%. *S. halstedii* and *S. corynorhini*, for which no information on tetracycline production is available, give 75.4 and 75.6%, respectively.

Relative to the canonical cluster of *S. rimosus*, the 24-gene core defined here does not include an ortholog of oxyO. The observation concerns the composition of the locus rather than the requirements of the pathway: absence from the locus does not by itself establish absence from the chromosome, and dispensability cannot be decided from genomic data. It does mean, however, that the gene set common to all intact loci examined here is smaller than the canonical one, and that oxyO is not part of what defines an intact cluster in this dataset.

### 2.5. Distribution of the Locus in the Genus

All assemblies of the genus *Streptomyces* of scaffold level or higher available in NCBI (GenBank and RefSeq; 13,172 unique assemblies after collapsing paired accessions, of which 13,171 remained after one assembly was found to lie outside the genus), together with the 27 genomes of the NPDC collection that carry no INSDC accession and are described in this section, giving 13,198 genomes in total, were screened with a probe of three proteins of different function—OxyA, OtcR and OtrA—by tblastn at an e-value of 1 × 10^−6^, an identity of at least 30% and a query coverage of at least 50%, with a requirement of co-localisation within 40 kb.

Under these criteria, 137 assemblies proved to be carriers (1.04% of the published genomes belonging to the genus *Streptomyces*), distributed across nine groups of indistinguishable type strains (Table 1; the full list of carrier assemblies with accession numbers is given in Table S6). The estimate is a lower bound for two measured reasons: the probe is biased towards intact clusters and fails on fragmented assemblies in which the three genes do not fit on a single contig. Wherever a species was re-examined separately, the estimate rises: in *S. varsoviensis*, the locus is detected in all six RefSeq assemblies rather than in three out of four, since in three of them, it is split between two contigs at one and the same point, into five genes over 6101 bp and 19 genes over 21,951 bp, which together correspond to an intact locus; in *S. rimosus*, a deep tblastn search yields 92 carriers out of 96 (95.8% [89.8–98.4]) against the 90 out of 96 reproduced by the probe; and in *S. platensis*, resolving the site by the species’ own flanks reveals four carriers instead of two. The ratio is nevertheless a sharp one: the locus is present in almost one per cent of the genomes of the genus, whereas reliably documented production of OTC is known for single strains, and in all confirmed producers, the locus has the same composition and the same gene order. A relaxed criterion without the requirement of co-localisation gives an upper estimate of 130 assemblies; on further relaxation, however, the hits cease to be OTC ones—two assemblies of *S. hygroscopicus* yield 17 genes of the locus within a 45 kb window at a median identity of 45%, and the corresponding antiSMASH region corresponds to chelocardin (BGC0000208).

**Table 1:** Distribution of the oxytetracycline locus across the genus *Streptomyces*. Rows are groups of type strains that cannot be told apart at the species level (*S. libani* and *S. glebosus* are within 98.5% ANI of *S. platensis*; *S. griseolus* and *S. halstedii*, 99.99%), and every assembly is assigned to a group by ANI to the type strains rather than by the name under which it is deposited, since the labels in this clade are unreliable (Section 2.4). Counts are those returned by the three-gene probe; where a species was subsequently re-examined by a deeper search or by resolving the site on its own flanks, the higher count is given in the text and is not carried into the table. Shares are given with 95% Wilson confidence intervals. Denominator: 13,171 unique GenBank and RefSeq assemblies of the genus together with the 27 genomes of the NPDC collection that are not deposited in NCBI. Species are named as the assemblies are deposited in NCBI. ^a^ The type strain of this species, DSM 40239, has since been transferred to the genus *Kitasatospora* on genomic and phenotypic grounds [47]. ^b^ Assembly GCF_008704475.1, deposited as *S. viridifaciens*, is *S. aureofaciens* by ANI (99.6% to the type strain)—the chlortetracycline producer, likewise transferred to *Kitasatospora* [47].

Group of Indistinguishable Type Strains	Assemblies in Scan	Carriers	Share, % [95% CI]	Tetracycline Production
*Streptomyces lydicamycinicus*	23	23	100 [85.7–100]	described in the present work (bja295)
*Streptomyces albofaciens*	1	1	100 [20.7–100]	not described
*Streptomyces viridifaciens* ^a^	1	1	100 [20.7–100]	tetracycline [48]
*Streptomyces rimosus*	96	90	93.8 [87.0–97.1]	oxytetracycline [24,25]
*Streptomyces varsoviensis*	4	3	75.0 [30.1–95.4]	oxytetracycline [27]
*Streptomyces corynorhini*	3	2	66.7 [20.8–93.9]	not described
*Streptomyces glebosus*/*Streptomyces libani*/*Streptomyces platensis*	35	2	5.7 [1.6–18.6]	terramycin patent (*S. platensis* ATCC 23948) [28]
*Streptomyces griseolus*/*Streptomyces halstedii*	54	3	5.6 [1.9–15.1]	not described
*Streptomyces aureofaciens* ^b^	1	1	100 [20.7–100]	chlortetracycline [17]
All remaining named groups (580 groups, no carriers)	6996	0	0 [0–0.05]	
Below the species threshold	5169	11	0.21 [0.12–0.38]	
No hit to a type strain	815	0	0 [0–0.47]	
Total	13,198	137	1.04 [0.88–1.23]	

For *S. lydicamycinicus*, the count was completed beyond GenBank. Thirty assemblies deposited under the species name in the Natural Products Discovery Center collection were screened with the same probe. Twenty-two of them carry the locus and eight do not, and the split coincides exactly with genome relatedness: the carriers lie at 97.48–99.66% ANI to bja295 and the non-carriers at 96.24–96.82%, so that the highest value among the non-carriers is lower than the lowest value among the carriers. The eight fall below the species threshold and outside the species; four of them are deposited in GenBank as *S. platensis* and two as *Streptomyces* sp. Eleven of the 22 carriers are the same strains as GenBank assemblies of the species—10 of them deposited under the species name and one, NPDC029466, deposited as *Streptomyces* sp. under accession GCA_055574515.1 and assigned to the species by ANI. One of the 22, NPDC029466 itself, falls outside the species on the criterion used for the genomospecies of Section 2.4: its dDDH to members of *S. lydicamycinicus* is 68.6–69.1%, below the 70% threshold, and it forms a separate genomospecies in Table S3; by ANI it lies at 97.39–97.52% from the species, whereas the members of the species are no less than 98.83% from one another. That leaves 23 unique strains in total—the 21 collection carriers that fall within the species together with the type strain NBRC 110027^T^ and MMS22-DDSA8. All 23 carry the locus (100%, 95% Wilson CI 85.7–100), and no assembly of the species remains unscreened (Table S7). Strain bja295 is the twenty-fourth genome of the species and also a carrier, but it is excluded from this frequency: it entered the study by selection for production, and counting it would bias the denominator by the very trait being measured. The split therefore marks the boundary of the genomospecies rather than a loss inside the lineage: by the ANI criterion used here, every genome that falls within the species carries the locus, and every genome that lacks it falls outside the species.

Two effects bound the interpretation of this frequency. The scan counts assemblies rather than independent genomes: in *S. rimosus*, where carriage is highest, the 90 carrier assemblies collapse to seven representatives at 99.9% ANI (Section 2.7), so the contribution of a heavily resequenced industrial producer to the genus-wide figure reflects sequencing effort and not biology. In the opposite direction, every species re-examined with a dedicated search yielded more carriers than the genus-wide probe. What the figure supports is therefore an order of magnitude—the locus is present in about one per cent of published *Streptomyces* assemblies—rather than a point estimate, and the informative part of the result is not the percentage but its structure: carriage is uniform within lineages and absent between them, which is the pattern expected of descent with loss rather than of dissemination among unrelated strains [7,10].

### 2.6. The Chromosomal Location of the Locus and the Occupancy of Its Site

The position of the locus in the chromosome is defined by a pair of 20 kb flanking regions adjoining its core in bja295; the definition was verified on bja295 itself, where the inter-anchor interval amounted to 27,547 bp. In carriers, the interval falls within 25,259–29,796 bp, that is, the spread does not exceed five per cent over almost 30 kilobases, which is consistent with a single point of insertion within the lineage. In a subset of the assemblies lacking the cluster, both flanking zones are recognised over 96–97% of their length and lie on one contig without breaks, while 1909–3421 bp remain between the anchors. This residual sequence is not a trace of excision of the cluster: it shares no 24-mers with the locus, gives no blastn hits at a word length of 11, contains neither IS elements nor terminal inverted repeats, and its GC content (70.2–71.7%) does not differ from that of the genome.

Of the 100 inter-anchor intervals excised across the genus, 32 contain biosynthetic clusters of no fewer than eight types—homologues of cetoniacytone A, bosamycin, toyocamycin, A54145, malleilactone, lavendiol, salinosporamide A and ethylenediamine-disuccinic acid—the OTC cluster proving to be merely the most frequent among them. The state of the site coincides with the boundaries of the genomospecies without exception. The definition of the site itself, however, holds within the *S. lydicamycinicus* lineage: in *S. platensis*, the flanking pair of bja295 is recognised, but the cluster lies 160 kb away from it, and when the site is defined by the species’ own flanks, the carriers give an interval of 29,165 bp with all 24 genes inside it and six non-carriers give 247 bp and nothing. The identical length of the residual interval in all six is consistent with a single deletion event inherited by descent rather than with six independent ones; nucleotide-level alignment of the residual intervals would be required to establish this directly.

The site is therefore better described as a recurrently occupied insertion point than as an address specific to the OTC cluster: in a third of the intervals excised across the genus, it holds a biosynthetic block of some kind, and which block it holds follows the lineage. Two consequences follow. The state of the site coincides with the boundaries of the genomospecies without exception, which places the turnover of its content at the level at which lineages diverge and not at the level of individual strains; and the absence of the cluster from a genome cannot be read as evidence that its lineage never carried it, since the residual interval left behind retains no trace of the excision.

### 2.7. Relationship Between the Cluster and Genome Topologies, and the History of the Locus

The trees of the cluster (maximum likelihood over the concatenated core proteins, LG + G4, 23 single-copy genes) and of the genome (neighbour joining over the ANI matrix) were compared over 42 de-duplicated profiles. Judged against an appropriate null distribution, the two topologies do not agree. One hundred trees inferred from random subsets of the same core genes on the same taxa differ from the concatenated genome tree by a normalised Robinson–Foulds distance [49] of 0.410 in the median (95th and 99th percentiles 0.487 and 0.513); this is the dispersion produced by sampling alone at this amount of data. The cluster tree lies beyond it, at 0.564 (*p* = 0.01, z = 3.07), and the discrepancy is not an artefact of information content: the locus alignment carries 2260 parsimony-informative columns against a median of 1148 in the null, and none of the 100 null subsets was more informative than the locus, so a better resolved rather than a worse matching tree was to be expected. An approximately unbiased test rejects the genome topology as an explanation of the locus data: constraining the locus to that topology costs 1052.6 log-likelihood units (*p*-AU = 6.3 × 10^−5^, and 2.1 × 10^−4^ for the genome topology itself), against *p*-AU = 1.000 for the unconstrained cluster tree. Reconciliation of the 23 gene families with the species tree points the same way: forbidding transfer requires 63 duplications and 342 losses, whereas allowing it requires 63 transfers and 44 losses, an improvement of 500.6 log-likelihood units for a single additional parameter. Of the 63 inferred transfers, 13 fall on one and the same donor–recipient pair (GCF_044330965.1, *S. halstedii* → GCF_055595265.1, *Streptomyces* sp.), which is the signature of the movement of a block rather than of individual genes; the remaining pairs are scattered over one to four genes each and, at the divergence of the genomes concerned (97.5–99.7% ANI), are not distinguishable from homologous recombination between close relatives. Global cophylogenetic statistics computed on the core-genome tree of 325 genomes of the genus—42 carriers and 283 non-carriers, 138 single-copy markers—place the two topologies closer to each other than to random expectation (congruence index I_cong = 1.688, Mantel r = 0.841 on patristic distances, Procrustean superimposition PACo m^2^ = 0.341, *p* ≤ 0.005 in each case against its own permutation null), which is a weaker statement than congruence and is not contradicted by the tests above. That baseline is the one rejected above for the Robinson–Foulds comparison: permuting the labels measures departure from randomness and not from the dispersion expected at this amount of data, so these three statistics establish only that the two topologies are not unrelated. They are reported for completeness and carry no weight in the conclusions drawn here, all of which rest on the null distribution built from random gene subsets, on the approximately unbiased test and on the reconciliation. We therefore do not claim that the cluster has descended with the genome throughout the genus: at least one transfer of the locus between carrier lineages is supported by all three lines of evidence. One qualification bounds this result—the reconciliation panel contains carrier genomes only, so it cannot address whether a lineage that now lacks the locus ever acquired one.

A Markov k-state (Mk) model with separate rates gives a ratio of the rate of loss to the rate of gain of 7.3 (χ^2^ = 40.9, df = 1, *p* = 1.6 × 10^−10^). The carriers are at the same time polyphyletic: they form 22 maximal clades, the largest of which includes 12 genomes together with bja295, while 14 are represented by single genomes. Carriage mapped onto the genus tree is shown in Figure S3. The minimum number of events on the tree at equal costs of gain and loss amounts to 11 gains and eight losses and is robust to the composition of the sample; at the cost ratio that follows from the estimated rates, the same parsimony gives two gains and 48 losses, that is, the particular number depends on the model, whereas the polyphyly of the carriers and the predominance of losses do not. The ancestral state was reconstructed on the species-level tree of the 66-genome panel, in which 17 taxa carry the locus. The minimum number of state changes under Fitch parsimony is three, and the model with separate rates is preferred over the model with a single rate (LR = 4.56, *p* = 0.033); averaging over 1000 stochastic character maps gives 7.9 gains and 7.1 losses. The ancestor of the *S. lydicamycinicus* clade, all 13 representatives of which are carriers, is reconstructed as a carrier with a probability of 0.9999, and the ancestor of the clade of three *S. platensis* carriers with a probability of 1.00. The clade uniting all 17 carriers of the panel is reconstructed as a carrier one with a probability of only 0.62, and at the next node 0.15, that is, deeper down the reconstruction converges on the equilibrium value of the model and does not resolve the question. The fourth carrier, *Streptomyces platensis* NPDC053371, lies outside the clade of the other three and gives an ANI of 94.95% to the type strain of the species, that is, it lies at the very boundary of the species; the carriers in this lineage therefore do not form a single group. Within these two clades, consequently, the locus is inherited vertically, and it was lost by the majority of the representatives of *S. platensis*, which is consistent both with the deletion scar and with the predominance of losses over gains. The statement is made at the level of these clades and is not extended to the genus as a whole.

Pangenome openness α—the exponent of Heaps’ law, which measures how fast the pangenome grows with each added genome—and genome fluidity φ—the mean fraction of genes not shared by a pair of genomes—were calculated for the principal carrier species: in *S. lydicamycinicus*, α = 0.52 (95% CI 0.39–0.66) and φ = 0.067 ± 0.019 (24 genomes); in *S. platensis*, α = 0.58 (0.37–0.79) and φ = 0.090 ± 0.036 (15 genomes); and in *S. rimosus*, α = 0.56 (0.48–0.63) and φ = 0.100 ± 0.010 (seven genomes after de-replication of 90 carrier assemblies at 99.9% ANI). No relationship between fluidity and carriage frequency is claimed here: the comparison rests on three species, no correlation was tested, and, as Table 1 shows, the species with the highest carriage frequency of the three (*S. rimosus*, 93.8%) also has the highest fluidity. Fluidity is used in this work for two related purposes. Since it is the mean fraction of genes not shared by a pair of genomes of one species, it is also the expectation for any new pair, and therefore states how much of its gene content a newly isolated member of the species may be expected to differ in: at φ = 0.067, the genomes of *S. lydicamycinicus* differ from one another by some seven per cent of their genes, which is the background against which the presence of the same 24-gene locus in every genome of the species has to be read. The second purpose is to gauge how much of each panel is clonal redundancy rather than diversity. Redundancy of the species panel was checked directly: collapsing genomes at 99.9% ANI leaves 18 representatives, and the pangenome recomputed on them together with bja295 (19 genomes) changes fluidity only in the third decimal (φ = 0.063 ± 0.018 against 0.067 ± 0.019 on the full panel), so the estimate for *S. lydicamycinicus* is not inflated by clonal redundancy. This is the opposite of the situation in *S. rimosus*, where the same procedure raises φ from 0.034 to 0.100 because 83 of 90 assemblies collapse into a single group. The values published for *S. rimosus*—α = 0.83 and φ = 0.12 [40]—were obtained on a panel of 32 predominantly draft assemblies with Roary; on re-analysis of that same panel with the present pipeline, the pangenome size (22,363 against “more than 22,000”) and the number of strain-specific genes (9779 against 9646) are reproduced, whereas φ = 0.172 and α = 0.46. Pangenome openness is therefore not comparable across tools, and only φ computed within one pipeline is used here.

### 2.8. Resistome, Defence Systems and the Meaning of the otr Genes

Screening of the predicted protein complement of bja295—the open reading frames (ORFs) called by Prokka—against CARD with RGI and, independently, with AMRFinderPlus v4.2.7 [50,51] returned 40 loci (Figure 3, ring 1). Nine reach curated confirmation and only four the Strict level: *otrA* (7.13 Mb, 72.6% identity), the rifamycin monooxygenase Rox, the rifamycin helicase HelR and the efflux protein QacJ; AMRFinderPlus additionally confirms two chloramphenicol efflux genes, a macrolide glycosyltransferase, a class A β-lactamase and the aminoglycoside phosphotransferase APH(3″)-Ia (7.82 Mb, 77.3% identity), the last accounting for the absence of a streptomycin zone observed in the susceptibility panel (Section 2.1, Table S2), although antiSMASH finds no streptomycin cluster. Aminoglycoside phosphotransferases unrelated to the strain’s own biosynthesis are a documented feature of the *S. rimosus* resistome [52,53], and APH(3″)-Id has been characterised in the type strain ATCC 10970^T^ [54], which represents *S. rimosus* in the present work as well. Six homologues fall in the tetracycline class, but only *otrA* is Strict: *otrB*, at 75.8% identity to the *S. rimosus* model, remains Loose, and the other four lie at background identity (4.3–16.0%) to the locus genes. The gap between 40 homologues and four Strict hits is consistent with a general expectation rather than being a demonstrated result: in actinomycetes, whose secondary metabolism is built on enzymes related to resistance determinants, homology-based resistome lists are expected to overcall, and the data presented here neither establish nor quantify the size of that effect.

Four defence systems were recovered, three of them by both DefenseFinder and PADLOC [55,56]: type II restriction–modification (4.27 Mb), the phosphorothioation system SspBCDE (5.58 Mb) and type IV restriction (6.93 Mb); a CARD_NLR-family endonuclease (0.56 Mb) is complete by DefenseFinder only. Two of them lie inside the bja295-specific blocks identified above, which suggests that those blocks are defence islands rather than annotation gaps. The CRISPR array of the genome was typed with CRISPRCasTyper [57]. The OTC locus itself lies in the part of the chromosome shared by all 23 genomes of the species examined here (Section 2.3) and not within these blocks, so the defence systems cannot be read as a strain-specific protection of the cluster; they mark the portion of the chromosome by which bja295 differs from its closest relatives.

Carriage of *otr* genes, finally, is not evidence of biosynthesis. In the sister lineage (ANI 91.8–93.2% to bja295), the biosynthetic core has been lost while *otrA* and *otrB* are retained; the complete locus is present in 1.04% of the assemblies of the genus, whereas tetracycline resistance determinants—both ribosomal protection and efflux—are among the most abundant classes in environmental resistomes and are regularly recovered from soils, sediments, manure and gut microbiota of bacteria for which tetracycline production has never been described [16,58,59,60]. The two scales differ accordingly: among 217 tetracycline-resistant streptomycete isolates from diverse habitats, *otrA* was detected in three (1.4%) and *otrB* in 13 (6.0%) [61], whereas the complete locus is present in 1.04% of the assemblies of the genus. Detection of *otrA* or *otrB* in a genome or a metagenome therefore says nothing about the capacity for biosynthesis—a distinction that matters for surveillance data, oxytetracycline remaining a first-line veterinary drug.

### 2.9. Summary of the Evidence

Three results of this work bear on how a biosynthetic locus is to be read from a genome. Carriage, structural integrity and demonstrated production are separable statements, and they are separated here by three different kinds of evidence. The locus is not scattered across the genus at the level of individual strains: within a lineage, it is either present in all genomes or absent from all of them, and the boundary between the two states coincides with the boundary of the genomospecies. And the chromosomal site that carries it is not specific to this cluster—a third of the intervals excised at the same address in other genomes hold biosynthetic blocks of at least eight other types.

Two paradigms are usually contrasted for the evolution of biosynthetic gene clusters: dissemination by horizontal transfer, which produces a mosaic distribution at the level of strains, and descent with loss, which produces a lineage-structured one. The distribution observed here is of the second kind, and the rate estimates make loss about seven times more probable than gain. The comparison of topologies, however, does not permit the stronger reading in which the cluster has simply followed the chromosome throughout the genus: at least one block-level transfer between carrier lineages is required by the data. The two statements are compatible—a locus may be lineage-structured overall and still move occasionally between related carriers—and together they place the OTC cluster between the two paradigms rather than at either of them. This agrees with what has been reported for the genus as a whole: recombination in *Streptomyces* is constrained by phylogeny rather than by geography, and the majority of secondary metabolite clusters are shared across all strains of a species and were therefore present in their common ancestor [42].

These estimates can be placed against what is known of gene transfer in the genus as a whole. Calibrating transfer against a dated phylogeny, McDonald and Currie found Streptomyces to be some 380 million years old and estimated that, averaged over that span, only about 10 genes per million years were acquired and subsequently retained, that is, on the order of one fixed acquisition per lineage every hundred thousand years [62]. A locus of the size treated here—24 genes inherited as one block—therefore amounts to roughly two and a half million years of ordinary accretion arriving at once, and the infrequency of such events is what the present data show directly: loss is estimated to be about seven times more probable than gain, and the topological evidence requires at least one transfer rather than many. The comparison is qualitative, since that work dates acquisitions across whole genomes, whereas the estimates here concern the fate of a single locus on an undated tree; but both point the same way, and neither is compatible with the promiscuous exchange often assumed for biosynthetic gene clusters. The framework applied here is also not particular to antibiotic biosynthesis: co-inheritance boundaries and tree comparison have long been used to describe genomic islands, of which the 500 kb symbiosis island of *Mesorhizobium loti*, integrating into a phe-tRNA gene and transmissible to non-symbiotic mesorhizobia, is the classical case [63]. The contrast with that case is as informative as the parallel. The symbiosis island carries its own integrase and transfer machinery, whereas the locus described here contains neither, nor is it framed by insertion elements (Section 2.4), and its site is not reconstructed on excision. The same contrast can be drawn within these genomes against the defence systems of Section 2.8, a class catalogued across the genus precisely because it moves readily between lineages [55]: in bja295, those systems lie in strain-specific blocks, whereas the OTC locus lies in the chromosomal region shared by all genomes of the species. Carriage of the locus is thus bound to lineage in a way that neither self-mobile islands nor defence systems are, and that is the sense in which it falls between the two paradigms. The locus treated here is a single example, and the pattern described for it is not put forward as a general rule for biosynthetic gene clusters; whether it holds more widely would have to be established on many loci rather than one.

### 2.10. Limitations

Seven limitations bound the conclusions above. The three-gene probe scores carriage but not integrity, and it fails on assemblies in which the locus is split between contigs, so all carriage frequencies are lower bounds. The set of published genomes is taxonomically uneven, and species represented by a single assembly cannot be distinguished from species in which the locus is genuinely rare. Production has been demonstrated for one strain of *S. lydicamycinicus*, and the silence of the locus in the remaining genomes of the species is inferred from the absence of reports rather than tested. Fermentation conditions were not optimised, so the titre reported here is a property of the medium as much as of the strain. Transfer between carrier lineages is inferred from tree comparison and from reconciliation, not from functional genetics: no transfer experiment was performed, and the reconciliation panel contains carrier genomes only, so the origin of the locus in the genus as a whole is not addressed. The absence of an oxyO ortholog is established for the locus, and a genome-wide search would be required to exclude a diverged copy elsewhere in the chromosome. Finally, the phenotypic measurements—the titre, the inhibition zones of Table S2 and the carbon-source utilisation of Table S1—rest on three independent replicates each, which is enough to establish the readings reported here but not to support comparisons between media or between strains.

## 3. Materials and Methods

### 3.1. Microbial Isolation and Cultivation

Strain bja295 was isolated from subtropical soil of the Ornithological Park, Sirius, Russia (43°23′53.14″ N, 39°58′34.67″ E; the site is documented in Figure S4). Sample collection was performed according to a previously described methodology [2,64]: a specimen from the A1 humus layer (10–15 cm depth) was collected using a sterile spatula and placed in a sterile sample container [65]. Actinobacteria were isolated through surface plating of ten-fold diluted soil suspensions spread over oatmeal agar (ISP3) [66] supplemented with nystatin (250 μg/mL; Biosintez, Penza, Russia) and nalidixic acid (10 μg/mL; Dia-M, Moscow, Russia) to suppress fungal and Gram-negative bacterial growth, respectively. Prior to inoculation, the soil sample was heat-treated at 100 °C for 2 min in a dry block thermostat. The plates were incubated for 14 days at 28 °C.

### 3.2. Phenotypic Characterisation

Cultural characteristics of strain bja295 (including aerial mycelium coloration and soluble pigment excretion) were observed on solid media recommended by the International *Streptomyces* Project after cultivation for up to 14 days at 28 °C [66], in three independent replicates.

Carbon-source utilisation (mono- and oligosaccharides, alcohols) was assessed via the disc-diffusion method (HiMedia Laboratories Pvt. Ltd., Thane, India) on ISP9 mineral basal medium with 0.04% bromcresol purple (Dia-M, Moscow, Russia) as a pH indicator, at 28 °C for 14 days, in three independent replicates. Hydrolysis of starch, of cellulose and of casein was tested on the corresponding solid media according to published protocols, by looking for a zone of clearing around the colony [2]; the results are given in Section 2.1.

Antibiotic susceptibility was determined by the disc-diffusion method. A spore suspension of strain bja295 (0.5 McFarland standard; HiMedia Laboratories Pvt. Ltd., Thane, India) was spread on ISP3 solid medium. After drying, antibiotic-impregnated discs (HiMedia Laboratories Pvt. Ltd., Thane, India) were applied, and the plates were incubated for 7 days at 28 °C, followed by measurement of inhibition zones. The assay was performed in three independent replicates, and Table S2 reports the means.

### 3.3. Optimisation of Cultivation Conditions

Actinomycetes obtained from primary plating were used for subsequent cultivation on solid media recommended by the International *Streptomyces* Project (ISP) at 28 °C for 14 days [66]; 28 °C is the temperature recommended by the ISP for the cultivation and characterisation of streptomycetes and is also the growth optimum of this strain; it was used throughout the work for both reasons. ISP2 was selected as the optimal medium for the production capacity of strain bja295. Which component of ISP2 accounts for the difference was not established: the media compared differ in both their carbon and their nitrogen sources, and no single-factor comparison was performed. Subsequent clonal resolution of bja295 was performed on five variants of solid nutrient media differing in carbon and nitrogen sources, as well as in the presence or absence of mineral salts: V1 (1% maltose (CDH, New Delhi, India), 0.5% beef extract (BD, Franklin Lakes, NJ, USA) 0.5% peptone (Angel Yeast Co., Ltd., Yichang, Hubei, China), 0.5% NaCl (CDH, New Delhi, India), 2.0% agar (Dia-M, Moscow, Russia), pH 7.0), V2 (1% glucose (CDH, New Delhi, India), 0.5% beef extract (BD, Franklin Lakes, NJ, USA), 0.5% peptone (Angel Yeast Co., Ltd., Yichang, Hubei, China), 0.3% NaCl (CDH, New Delhi, India), 2.0% agar (Dia-M, Moscow, Russia), pH 7.0) [67], V3 (1.5% malt extract (CDH, New Delhi, India), 0.5% yeast extract (Angel Yeast Co., Ltd., Yichang, Hubei, China), 0.5% soluble starch (Chimreaktivsnab, Ufa, Russia), 2.0% agar (Dia-M, Moscow, Russia), pH 7.5) [66], V4 (2.0% soluble starch (Chimreaktivsnab, Ufa, Russia), 0.1% KNO_3_ (CDH, New Delhi, India), 0.05% K_2_HPO_4_ (neoFroxx, Einhausen, Germany), 0.05% MgSO_4_ (CDH, New Delhi, India), 0.05% NaCl (CDH, New Delhi, India), 0.001% FeSO_4_ (CDH, New Delhi, India), 2.0% agar (Dia-M, Moscow, Russia) [68], and V5 (0.05% K_2_HPO_4_ (neoFroxx, Einhausen, Germany), 0.03% casein (CDH, New Delhi, India), 0.1% yeast extract (Angel Yeast Co., Ltd., Yichang, Hubei, China), 0.1% peptone (Angel Yeast Co., Ltd., Yichang, Hubei, China), 1% maltose (CDH, New Delhi, India), 2.0% agar (Dia-M, Moscow, Russia), pH 7.0) [69]. Cultivation of bja295 clones was carried out at 28 °C for 14 days, in three independent replicates. The composition and pH of every medium used in this work are summarised in Table S8. A component-by-component comparison of the media of defined composition is given in Table S9.

For submerged cultivation, V3 and V5 were selected as the nutrient medium variants. Production of secondary metabolites by bja295 clones was performed over 14 days at 28 °C under continuous agitation on an orbital shaker (New Brunswick Innova, Eppendorf, Hamburg, Germany) at 200 rpm.

### 3.4. Test Microorganisms

Antibacterial activity of the bioactive compound was tested against the bacterial strain *E. coli* JW5503 Δ*tolC*, characterised by a Δ*tolC* gene deletion that disturbs the efflux pump, and the strain *E. coli* BW25113 *lptD*, featuring a deletion in the *lptD* gene (codons 330–352) leading to impaired lipopolysaccharide layer synthesis and consequently increased permeability to low-molecular-weight compounds [11]. For simplicity, these strains are hereinafter referred to as Δ*tolC* and *lptD*, respectively.

These strains carry the plasmid vector pDualrep2, which encodes the fluorescent proteins TurboRFP and Katushka2S [11]. Expression of these reporter proteins is induced by compounds at sublethal concentrations that either trigger the SOS response or inhibit translation, respectively. Fluorescence signals were quantified 24 h post treatment using a ChemiDoc MP imaging system (Bio-Rad, Hercules, CA, USA) with Image Lab v6.0.1 and predefined optical channels (Cy3 and Cy5 filters), enabling selective detection of TurboRFP and Katushka2S emission spectra.

*Staphylococcus aureus* ATCC 29213 was used as the test organism for minimum inhibitory concentration (MIC) determination.

### 3.5. Analysis of Bioactive Compounds

The reporter strain screening was performed using an agar diffusion assay, as previously described [2]. Strain bja295 was cultured on ISP2 medium, with testing conducted on days 3, 6, 9 and 12 of growth. Agar blocks (5 mm diameter) were aseptically excised from sporulated colonial lawns and placed onto LB agar plates pre-inoculated with the appropriate test organism. Each combination of medium, growth day and test strain was assayed in three independent replicates.

### 3.6. Purification of Active Compounds

For detailed bioactivity studies, strain bja295 was cultured in CSPY-ME medium (V5, Table S8) [69] at 28 °C for 14 days under continuous agitation (200 rpm) using a New Brunswick Innova 44 orbital shaker (Eppendorf, Hamburg, Germany). The culture broth was separated from the biomass by centrifugation at 4000× *g*. The resulting cell-free supernatant was acidified with 1% formic acid (SPANLAB, New Delhi, India) to pH 4.0 and subsequently clarified with activated charcoal (1% *w*/*v* of the total volume). The clarified supernatant was then loaded onto a Poly-Prep Econo-Pac column (Bio-Rad, Hercules, CA, USA) packed with 1 mL of LPS-500H sorbent (Technosorbent, Moscow, Russia). After complete binding of the sample volume, stepwise elution was performed using a water–acetonitrile gradient, with fraction collection, and the antagonistic activity of the collected fractions was assessed by agar diffusion assay as described above. The two chromatographic steps serve different purposes and are not alternatives: solid-phase extraction and RP-HPLC separate the active compound from the rest of the culture fluid, whereas mass spectrometry identifies what has been separated. The active fractions were subjected to further purification by reversed-phase high-performance liquid chromatography (RP-HPLC) on an Agilent 1260 series system (Waldbronn, Germany) equipped with a diode array detector (DAD) and an automated fraction collector. Chromatographic separation was achieved on a Luna C18(2) column (5 μm, 100 Å, 250 × 4.6 mm; Phenomenex, Torrance, CA, USA) using the water–acetonitrile gradient programme given in Table S10.

### 3.7. Mass Spectrometry and Dereplication

The active fractions obtained from the bja295 sample were analysed using a chromatographic–mass spectrometric system comprising an UltiMate 3000 UHPLC system (Thermo Fisher Scientific, Waltham, MA, USA) equipped with an Acclaim RSLC 120 C18 column (2.2 μm, 2.1 × 100 mm; Thermo Fisher Scientific) coupled to an ESI-qTOF mass spectrometer (maXis II 4G ETD; Bruker Daltonics, Bremen, Germany). Data acquisition was performed in full-scan mode over a mass range of *m*/*z* 100–1500, with MS/MS fragmentation of the three most intense precursor ions per cycle using collision-induced dissociation (CID) at 10–40 eV. Mass spectra were processed using OpenChrom Lablicate Edition (version 1.4.0.202201211106) and TOPPView v2.3.0 [70]. Putative chemical structures were identified by database matching against the Global Natural Products Social Molecular Networking (GNPS) platform [71], NPAtlas [72,73], and the Dictionary of Natural Products (version 31.1).

### 3.8. Microbiological Broth Microdilution Assay

Oxytetracycline quantification was performed by a serial two-fold broth microdilution assay in 96-well plates, according to the British Pharmacopoeia [74]. A veterinary OTC powder (1 g, OTC hydrochloride, powder for solution for injections), used as a reference standard, was dissolved in deionised water, sterilized by filtration through a 0.45 μm membrane filter, and serially diluted in Mueller–Hinton broth over the concentration range of 0.125–256 µg/mL. *Staphylococcus aureus* was used as the test organism at a final density of 5 × 10^5^ CFU/mL. Following incubation at 37 °C for 18–24 h, growth inhibition was monitored by OD_600_ readings. The concentration of OTC in the samples was determined by comparison with the reference standard calibration curve. All experiments were carried out in triplicate.

### 3.9. Genome Sequencing, Assembly, Annotation and Taxonomic Assignment

Genomic DNA of strain bja295 was isolated as described previously [75] and sequenced on Illumina HiSeq 4000 (Illumina, San Diego, CA, USA) and Oxford Nanopore MinION (Oxford Nanopore Technologies, Oxford, UK) platforms. Long reads were assembled with Flye v2.9.6 [76] and polished with Illumina reads using Polypolish v0.6.0 [77,78]. All coordinates in this work refer to the deposited assembly. Completeness was assessed with BUSCO v5.8.2 [79] against actinobacteria_class_odb10 (*n* = 356), annotation was performed with Bakta v1.12.0 [80] with database v6.0 (light) and with Prokka v1.14.6 [81], and small replicons were classified with geNomad v1.12.0 (database v1.9) [82]. Biosynthetic gene clusters were predicted with antiSMASH v8.0.4 [6] and compared with MIBiG v3.1 [83] through KnownClusterBlast; clusters are reported by protocluster core coordinates rather than by region coordinates, which include 20–35 kb of flanking sequence.

The genome was analysed on the Type (Strain) Genome Server [84,85], which computes Genome BLAST Distance Phylogeny distances, derives digital DNA–DNA hybridisation values by formula d4 [86] and infers balanced minimum-evolution trees by formula d5 with FastME v2.1.6.1 [87] with 100 pseudo-bootstrap replicates; the genome was automatically compared against all type strain genomes available in the TYGS database using the MASH algorithm [88], and 16S rRNA gene and whole-genome trees were inferred over the same panel of type strains and are given as Figure S2. The trees of Figure 2 were inferred from alignments over a panel of 24 genomes: 16S rRNA gene sequences were extracted with barrnap v1.10.6 [89], which wraps Infernal v1.1.5 with Rfam covariance models, taking the single longest complete copy per genome; sequences shorter than 900 bp were discarded with SeqKit v2.10.0, which left 22 of the 24 assemblies—one yielded no 16S call at all and one only a 53 bp fragment. The retained sequences were aligned with MAFFT v7.520 [90] under --auto, which selected the L-INS-i strategy, and analysed in IQ-TREE v2.3.6 [91,92] under TN + F + I + G4; the core-genome tree was inferred from a concatenate of 138 single-copy markers of the Actinobacteria set identified and aligned with GToTree v1.8.10 [93] and analysed in the same program under Q.insect + F + R4. Both models were selected by ModelFinder as implemented in IQ-TREE v2.3.6 [94] under BIC, and both runs used 1000 ultrafast bootstrap and 1000 SH-aLRT replicates. Both trees were built on the assembly of bja295 obtained before the terminal duplication described in Section 2.3 was resolved; the markers and the 16S genes lie in the conserved part of the chromosome and are identical in the deposited record. Average nucleotide identity was computed with skani v0.3.2 [95] (-s 70 --min-af 5 --median) and, independently, with FastANI [96]. Genomospecies within the complex were delimited from digital DNA–DNA hybridisation values over a panel of 46 genomes at the 70% threshold, and the corresponding ANI threshold was determined from the distribution of 1035 pairwise comparisons over the same panel, which is given in full in Table S4. Values within and between genomospecies do not overlap (97.77–100.00% and 95.68–97.52%, respectively), so that any threshold between 97.5% and 97.8% separates them without error; FastANI on the same panel gives a comparably clean separation at approximately 96.8%, that is, the value of the threshold depends on the implementation and is reported here together with it.

### 3.10. Comparative Genomic Analysis

Three panels are used throughout and are not interchangeable. The genus scan comprises 13,198 genomes: 13,171 of the 13,172 unique GenBank and RefSeq assemblies described below, one having been found by ANI to lie outside the genus and excluded, together with the 27 genomes of the NPDC collection that carry no INSDC accession. The species panel comprises the 24 unique strains of *S. lydicamycinicus*—bja295, 13 GenBank assemblies and 30 assemblies of the NPDC collection, of which 22 fall within the species by ANI and 11 duplicate GenBank strains; strain identity between the two sources was established from the NCBI strain qualifier and verified by ANI (all eleven pairs at 100%). One of the 22, NPDC029466, falls outside the species by dDDH and is not part of the panel, for the reason given in Section 2.5. Carriage frequency is reported over the 23 strains other than bja295. The de-replicated species panel is obtained from it by single-linkage collapsing at 99.9% ANI, which leaves 18 representatives. Pangenome statistics are reported both on the full-species panel together with strain bja295 (24 genomes) and on these representatives together with bja295 (19 genomes), since four pairs lie within 0.05 percentage points of the collapsing threshold, and the number of representatives is therefore not stable to the choice of ANI implementation. Each result states the panel on which it was obtained; the panels used throughout the work, their sizes and their purposes are summarised in Table S11.

Genomes of all panels were annotated uniformly with Prokka v1.14.6 [81] (–genus *Streptomyces*, –kingdom Bacteria, genetic code 11, minimum contig length 200 bp). The pangenome was built with Panaroo v1.8.0 in –clean-mode strict with a core-genome threshold of 95%; for comparability with published data, the same set was run with Roary v3.13.0 at an identity threshold of 95%. Pangenome openness α (Heaps’ law, *n* = κ·N^(−α)) and genome fluidity φ were computed with micropan v2.1; φ was computed exactly over all C(N,2) pairs of genomes and α over 1000 random permutations of genome order, with confidence intervals obtained by leave-one-genome-out jackknife. Rarefaction to a common sample size was performed at N = 8 and N = 13 with 200 replicates. Assembly quality was assessed with CheckM2 v1.1.0, and the calculations were duplicated on the subset with completeness ≥ 98%, contamination ≤ 2% and no more than 100 contigs. Panel redundancy was assessed with skani [95]; the *S. rimosus* panel was de-replicated at an ANI threshold of 99.9%.

The screen covered every *Streptomyces* assembly of scaffold level or higher available in GenBank and RefSeq: 25,546 accessions were queried, of which 25,542 were retrievable at the time of the scan (11 May 2026). These correspond to 13,172 unique assemblies, 12,370 of which are deposited under both a GCA and a GCF accession; all frequencies are reported over the unique set. The genomes of the NPDC collection that carry no INSDC accession were screened with the same probe and are included in the totals reported for the genus, which therefore cover 13,198 genomes; over the NCBI assemblies alone, the carriage frequency is 0.95%. The OTC locus was detected by tblastn [97], with OxyA, OtcR and OtrA as queries at a minimum identity of 30% and a minimum query coverage of 50%; an assembly was scored as a carrier only when all three hits lay on one contig within 40 kb. Because the probe requires all three genes above these thresholds and in co-localisation, degraded and fragmented loci are missed, and carriage frequencies are therefore reported as lower bounds.

Locus boundaries were defined by co-inheritance. The ±40 kb window around *otrA*/*otrB* of *S. rimosus* subsp. *rimosus* ATCC 10970^T^ (87 proteins) was searched by tblastn against all carriers, the fraction of carriers retaining each homologue within the same locus was computed, and the boundaries were placed at the two abrupt steps of the resulting profile; the procedure was repeated with bja295 as the reference. The chromosomal address of the locus was defined by two 20 kb anchors flanking its core in bja295: anchors were located by exact 24-mer matching on both strands, and the inter-anchor interval was measured. Intervals were excised where both anchors lay on one contig, characterised with antiSMASH v8.0.4, and compared by the fraction of shared canonical 24-mers and by blastn (-word_size 11 -evalue 1 × 10^−3^); protein domains within them were assigned with HMMER v3.4 [98] against Pfam-A v38.2 [99]. Residual intervals in non-carriers were additionally tested for shared 24-mers with the locus, insertion sequences, terminal inverted repeats and deviation of G + C content from the genome mean. For assemblies deposited under paired GCA and GCF accessions, both members returned identical anchor coordinates, inter-anchor intervals and site classes, confirming that the procedure is deterministic.

The chromosome was compared with related genomes without alignment, following the FracMinHash framework [100,101]: canonical 21-mers, a 1/32 hash subsample, and non-overlapping 5 kb windows of the reference chromosome, with a window scored as present at a shared fraction of 0.20 or above. The threshold was taken from the bimodal distribution of the statistic itself rather than set a priori. Since the shared fraction *p* relates to nucleotide identity id as *p* ≈ id^21^ [102], this corresponds to approximately 93% identity; robustness was verified by scanning the threshold from 0.02 to 0.80, and adjacent windows in the same state were merged into blocks.

Cluster phylogeny was inferred by maximum likelihood from concatenated core locus proteins aligned with MAFFT v7.520 (L-INS-i) [90] under LG + G4 with 1000 ultrafast bootstrap replicates in IQ-TREE v2.3.6 [91,92]; the genome phylogeny was inferred by neighbour joining from the skani ANI matrix over the same set of profiles, obtained by collapsing carriers with identical presence/absence and identity patterns across the 24 locus genes. Agreement between the two topologies was assessed in three ways. A null distribution for the normalised Robinson–Foulds distance [49] was built from 100 trees inferred from random subsets of the 23 single-copy core genes on the same 42 taxa (LG + G4, IQ-TREE v2.3.6, fast search, seeds 1–100), and the observed distance was referred to that distribution; leaf-label permutation was not used as a baseline, since it measures departure from randomness rather than from the dispersion expected at this amount of data. Competing topologies were compared by the approximately unbiased test in IQ-TREE v2.3.6, with the alignment partitioned by gene and substitution models selected separately for each partition. Reconciliation of the gene families with the species tree was performed with GeneRax v2.0.4 [103] (Software v2.0.4 available online: https://github.com/BenoitMorel/GeneRax (accessed on 14 September 2026)) under the UndatedDL and UndatedDTL models over the 23 families, the species tree being rooted with S. aureofaciens as outgroup before pruning to the 42 carriers. The congruence index I_cong [104], a Mantel test on patristic distances [105] and Procrustean superimposition [106] were computed on the core-genome phylogeny of 325 genomes (42 carriers and 283 non-carriers, 138 single-copy markers, IQ-TREE v2.3.6), each against its own permutation null; that baseline is subject to the same objection as leaf-label permutation above, and the three statistics are therefore reported without being used in support of any conclusion.

The core-genome phylogeny of the genus was inferred over a panel of 325 genomes: the 42 de-replicated carrier profiles; up to seven nearest non-carrier neighbours of each carrier by ANI; three non-carriers drawn from each of the ANI intervals 92–95, 90–92, 88–90, 85–88 and 80–85%; the complete assembly sets of *S. platensis*, *S. nigrescens*, *S. libani* and *S. halstedii*; and four outgroups. A concatenate of 138 single-copy markers of the Actinobacteria set was built with GToTree v1.8.10 [93] (31,050 amino acid positions; marker completeness 95.7–100% in every genome) and analysed in IQ-TREE v2.3.6 [91,92] under Q.insect + F + I + R6 selected by ModelFinder as implemented in IQ-TREE v2.3.6 [94], with 1000 ultrafast bootstrap and 1000 SH-aLRT replicates; the tree was rooted on *S. aureofaciens* and is shown in Figure S3. Presence of the locus was treated as a binary character on this phylogeny. Gain and loss rates were estimated under the Mk model [107] with equal and with separate rates using *ace* in *ape* [108] and compared by likelihood ratio test; event counts were obtained by Fitch parsimony under equal costs and under costs derived from the estimated rate ratio, and ancestral states were reconstructed on a species-level tree of 66 genomes by marginal likelihood and by averaging over 1000 stochastic character maps in phytools v1.9.16 [109].

Similarity between biosynthetic gene clusters was computed with BiG-SCAPE v2.0.3 [46,110] in cluster mode with –legacy-weights and a cut-off of 0.30, with MIBiG entries supplied as ordinary inputs; insertion sequences were predicted with ISEScan v1.7.2.3 [111] and tested against 176 control windows equal in length to the locus. Resistance determinants were identified with the Resistance Gene Identifier (RGI) v6.0.8 against CARD v4.0.1 [50], retaining the Perfect, Strict and Loose levels, and independently with AMRFinderPlus v4.2.7 [51]; both are reported. Defence systems were predicted with DefenseFinder v3.0.0 [55] and PADLOC v2.0.0 [56], retaining systems recovered by both, and CRISPR arrays with CRISPRCasTyper v1.8.0 [57]. Proportions are reported with numerator and denominator, with Wilson score confidence intervals [112]; enrichment was assessed against the empirical null distributions described above.

### 3.11. Statistical Analysis

Phenotypic assays—carbon-source utilisation, antibiotic susceptibility, the reporter-based activity screen, the comparison of cultivation media and the determination of the OTC titre—were performed in three independent replicates (*n* = 3). The read-outs differ in kind and are reported accordingly. Carbon-source utilisation and the reporter-based screen are qualitative, scored as growth or no growth and as the presence or absence of an induced signal, and gave identical results in all three replicates, so no measure of dispersion applies to them. Inhibition zone diameters are reported as the mean and the standard deviation of three independent biological replicates, with the individual readings given in Table S2. The titre was determined against a pharmacopoeial standard by a two-fold dilution series, which resolves concentration only to the nearest doubling; it is therefore reported as a single value, and a dispersion estimate would not be meaningful at that resolution. Genome quality is expressed as BUSCO completeness and as CheckM2 completeness and contamination. Branch support in the maximum-likelihood trees is given as SH-aLRT and ultrafast bootstrap values from 1000 replicates each, and in the trees inferred from GBDP distances as pseudo-bootstrap support from 100 replications. Every proportion is reported with its numerator, its denominator and a 95% Wilson score confidence interval [112]. Confidence intervals for genome fluidity and for pangenome openness were obtained by leave-one-genome-out jackknife, and the ratio of the rate of loss to the rate of gain was tested by likelihood ratio. Wherever a statistic referred to a null distribution—the normalised Robinson–Foulds distance, the congruence index, the Mantel and Procrustes tests and the enrichment of insertion sequences—the null was generated by permutation or by resampling, as described in the corresponding section, and the number of replicates is stated together with the result. No statistical test was applied to the comparison of fluidity between the three species, and none is claimed: the comparison rests on three panels of unequal size.

## 4. Conclusions

Across the known producers and their relatives, the OTC cluster is defined by synteny and by the homology of its genes rather than by the mere presence of individual genes. In every genome examined here in which the locus is intact, the same 24 genes occur in the same order, without rearrangements, gene replacements or insertions within the core; wherever this order is disturbed—the loss of *oxyM*, *oxyP* and *otcG* and the transfer of *otcR* in *S. corynorhini*; the IS110 element inside the core of *S. varsoviensis*; the 34.8 kb block with an altered gene order in the most distant carrier—production has not been described. The identity of the locus proteins, in contrast, varies continuously from 99.6 to 53.3% and separates neither producers from non-producers nor carriers from one another. Carriage, integrity and production are therefore three distinct statements about a genome: the first is read off a database scan, the second requires gene-by-gene comparison of the locus, and the third requires chemistry. Documented production and sequenced assemblies do not always refer to the same strain: for *S. varsoviensis*, production is reported in the earlier literature [27], whereas the assemblies examined here carry an IS110 element within the core, and it could not be established whether the two refer to one strain. Statements about production are therefore made at the level of the strain wherever the strain is identifiable.

Within each carrier lineage, the locus occupies a single synteny site, its position reproduced to within five per cent over almost 30 kilobases, which corresponds to a single insertion event per lineage; the acceptor lineages then diverged, and the site was either retained, or lost with a scar-free residual interval, or occupied by unrelated biosynthetic blocks. Losses outnumber gains by an estimated factor of about seven. Whether the locus arose once in an ancestor common to all carriers is not resolved by these data: the carriers are polyphyletic, ancestral-state reconstruction converges on the equilibrium value of the model at the deeper nodes, and parsimony under equal costs requires 11 independent gains. Comparison of the cluster and genome topologies against an explicit null distribution, moreover, rejects strict co-descent and supports at least one transfer of the locus between carrier lineages. Vertical inheritance is therefore established within clades—*S. lydicamycinicus* and *S. platensis*—and not for the genus as a whole. In strain bja295, all three properties coincide: the locus is present, its structure is intact, and the product is identified chemically—the first such demonstration for *S. lydicamycinicus*.

In bja295, the intact locus comprises 24 genes and does not include an ortholog of oxyO, a gene present in the canonical cluster of *S. rimosus*. Since this strain produces OTC, the gene is not required for the trait in the genetic background examined here, provided that it is indeed absent from the chromosome as a whole; a genome-wide search, and ultimately inactivation and complementation in a reference producer, would be required before dispensability could be stated formally.

Two lines of work follow directly. A panel that includes non-carriers is required before the origin of the locus in the genus can be addressed, since reconciliation over carriers alone cannot ask whether a lineage that now lacks the cluster ever had one. And if the reduced gene composition is confirmed at the level of the chromosome, it defines a smaller cassette for heterologous expression than the canonical cluster, which is of practical interest for the engineering of tetracycline biosynthesis.

## Data Availability

The nucleotide sequence of the oxytetracycline biosynthetic gene cluster from *Streptomyces lydicamycinicus* bja295 has been deposited in GenBank under accession number PZ777511 (BioProject PRJNA1499510, BioSample SAMN61881016). The genome assembly of strain bja295 has been deposited under BioProject PRJNA1499510 (BioSample SAMN61881016) and will be publicly available on release; the assembly and its annotation are also provided as Supplementary Materials accompanying this paper.

## Supplementary Materials

The following supporting information can be downloaded at: https://www.mdpi.com/article/10.3390/ijms27188403/s1.

## Abbreviations

The following abbreviations are used in this manuscript:

SPE	solid-phase extraction
CFS	cell-free supernatant
BGC	biosynthetic gene cluster
OTC	oxytetracycline
OSMAC	One Strain Many Compounds
NPDC	Natural Products Discovery Center
ISP	International *Streptomyces* Project
ANI	Average Nucleotide Identity
NGS	next-generation sequencing
ORF	open reading frame
dDDH	digital DNA–DNA hybridisation
MIC	minimum inhibitory concentration
HPLC	high-performance liquid chromatography
RP-HPLC	reversed-phase high-performance liquid chromatography
LC–MS	liquid chromatography–mass spectrometry
MS/MS	tandem mass spectrometry
UV	ultraviolet
CI	confidence interval
IS	insertion sequence
CARD	Comprehensive Antibiotic Resistance Database
RGI	Resistance Gene Identifier
GBDP	Genome BLAST Distance Phylogeny
TYGS	Type (Strain) Genome Server
SH-aLRT	Shimodaira–Hasegawa-like approximate likelihood ratio test
AU	approximately unbiased (test)
SARP	*Streptomyces* antibiotic regulatory protein
LAL	large ATP-binding regulator of the LuxR family
CRISPR	clustered regularly interspaced short palindromic repeats
INSDC	International Nucleotide Sequence Database Collaboration
