# Supplementary material for: The Oxytetracycline Producer Streptomyces lydicamycinicus bja295 and Its Genomic Context
Source: Int J Mol Sci. 2026 Sep 21;27(18):8403. doi: 10.3390/ijms27188403 (PMC13607094; doi:10.3390/ijms27188403)
Supplement: Supplementary file 1 [file ijms-27-08403-s001.zip › ijms-4518041-supplementary.pdf]

Supplementary Materials

Figure S1: LC–MS analysis of the active fraction of strain bja295. Top: base peak chromatogram of the active peak. Bottom, from top to bottom: the mass spectrum recorded at the apex of the peak (scan 322) and the MS/MS spectra of the ions at m/z 461.1517 (scan 323), 443.1407 (scan 324) and 283.1885 (scan 325), with the collision energy ramp given for each. Panels are reproduced from the acquisition software.

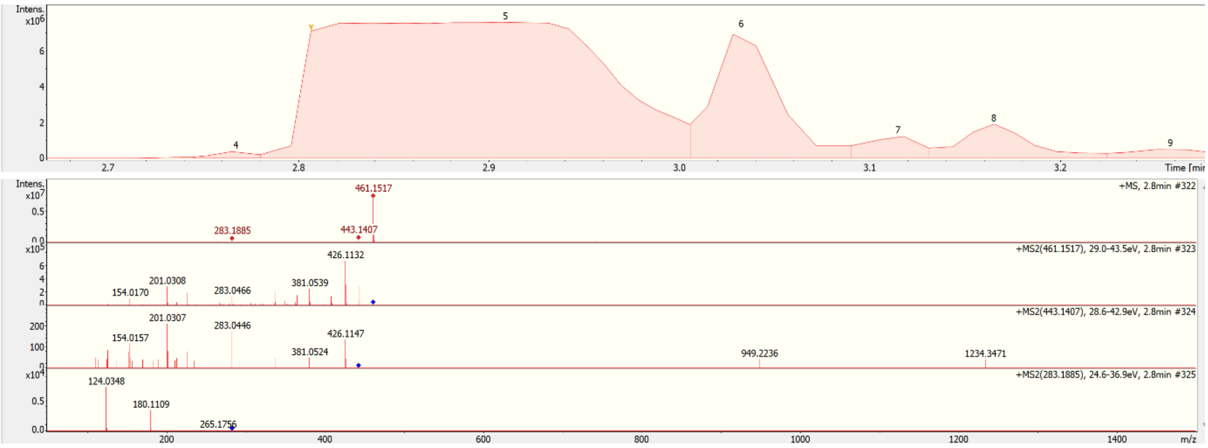

Figure S2: Sampling site of the soil from which strain bja295 was isolated. (a) Location of the ornithological park of the Imeretinskaya Lowland on the north-eastern coast of the Black Sea (left) and a satellite view of the park and its surroundings (right, red frame); the red star marks the sampling point, 43°23'53.14" N, 39°58'34.67" E, federal territory of Sirius, Sochi, Russia. (b) The *Yucca recurvifolia* individual beneath which the sample was taken. The sample was collected from the A1 humus horizon at a depth of 10–15 cm. The 100 km scale bar on the overview map applies at the latitude of the sampling point; satellite imagery © 2026 Google.

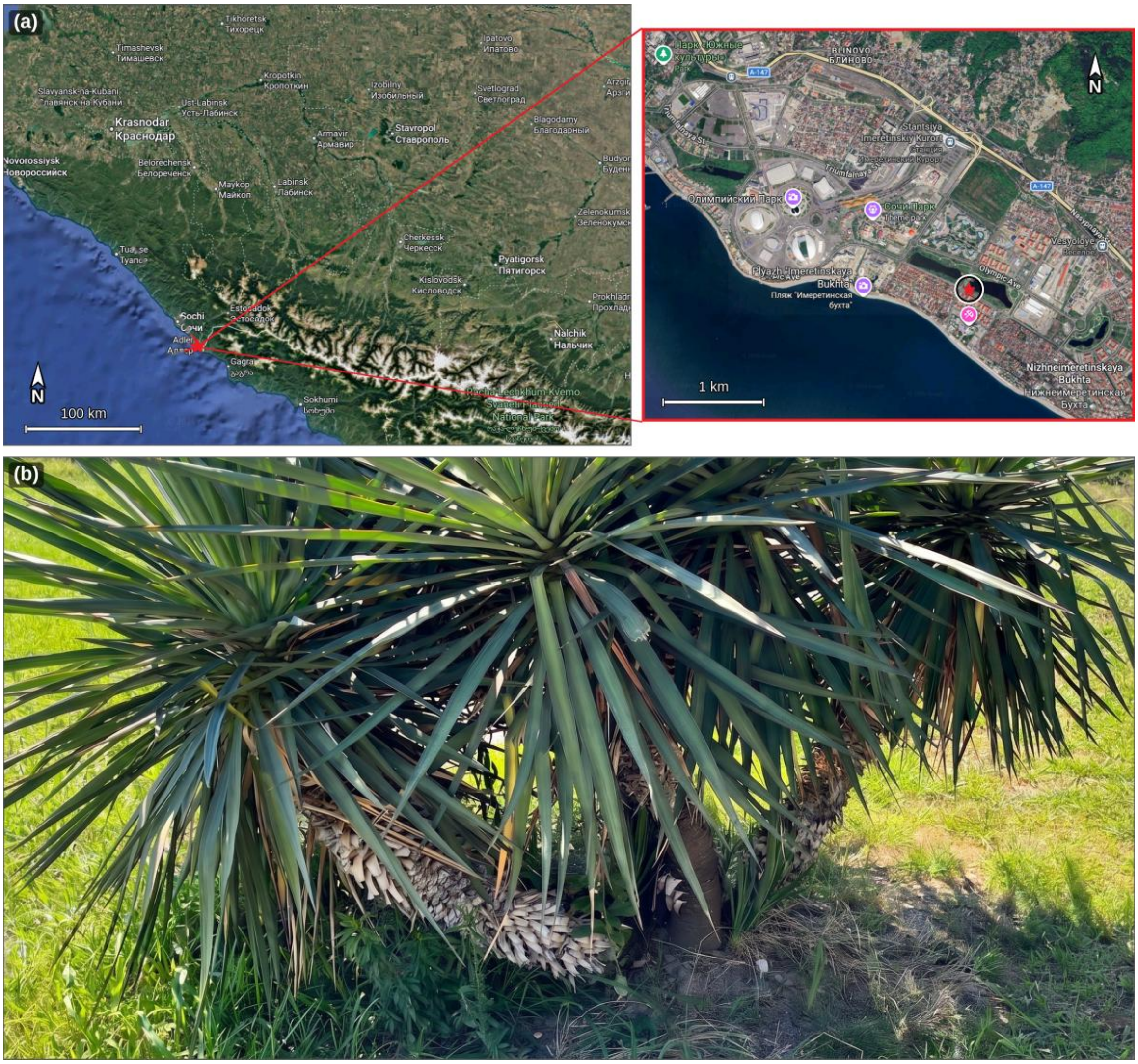

Figure S3: Phylogenetic position of strain bja295 from Genome BLAST Distance Phylogeny (GBDP) distances, shown for comparison with the maximum-likelihood trees of Figure 2. (a) Tree inferred from GBDP distances (formula d5) calculated from 16S rRNA gene sequences; 21 taxa. (b) Tree inferred from GBDP distances (formula d5) calculated from whole genome sequences; 23 taxa. Both trees were inferred with FastME 2.1.6.1 including SPR postprocessing on the Type (Strain) Genome Server and were rooted at the midpoint; numbers at the branches are GBDP pseudo-bootstrap support values from 100 replications, values below 50 are not shown. Type strains are marked with a T; strain bja295 is shown in red. The placement of bja295 as sister to the type strain of S. lydicamycinicus is the same as in Figure 2.

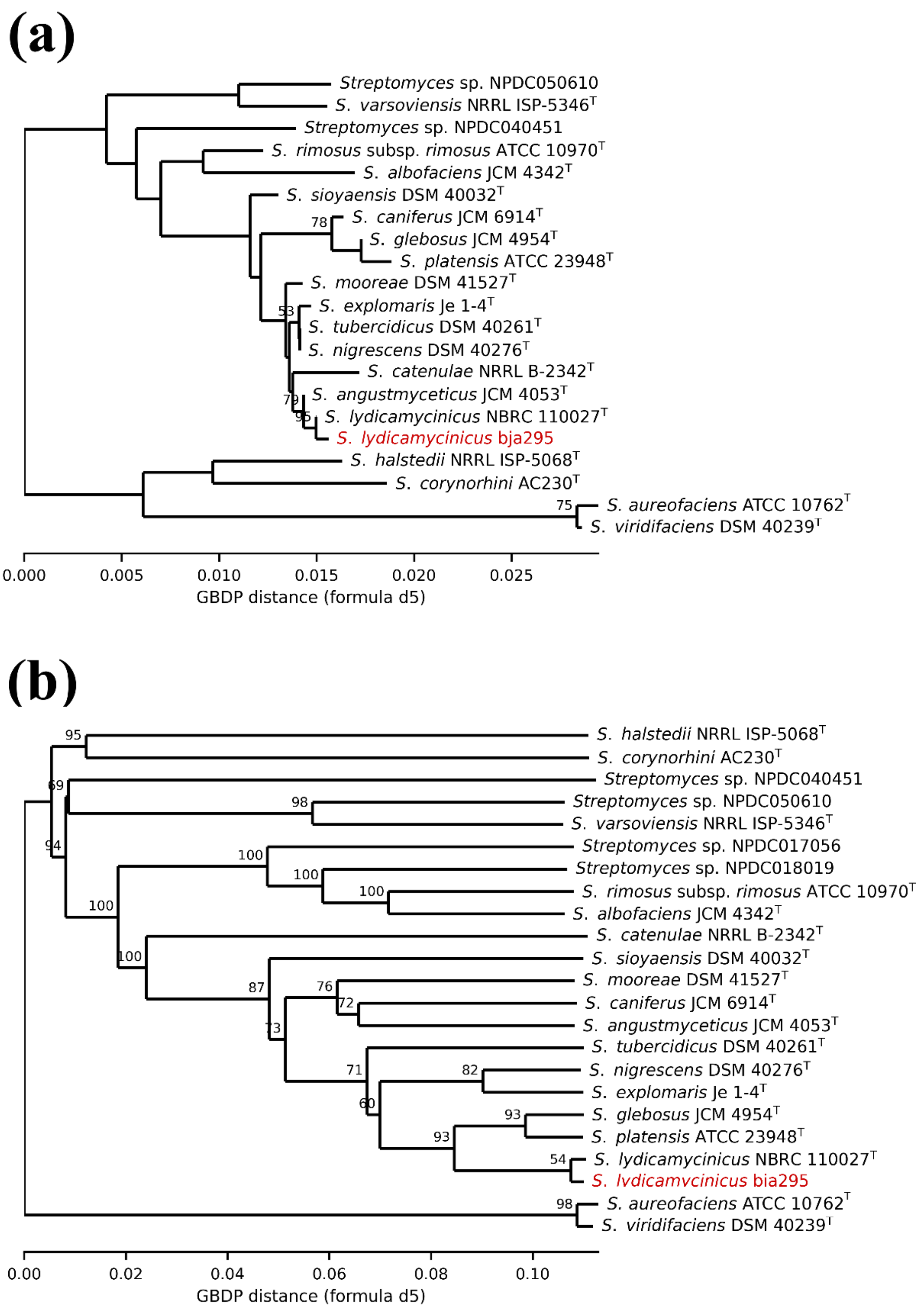

Figure S4: Carriage of the oxytetracycline locus on the core-genome tree of the genus Streptomyces. Maximum-likelihood tree of 325 genomes inferred from a concatenate of 138 single-copy markers of the Actinobacteria set (31,050 amino-acid positions; IQ-TREE v2.3.6, model Q.insect+F+I+R6) and rooted on S. aureofaciens; the topology is drawn as a cladogram, so branch lengths carry no information. The inner ring marks the 42 carriers of the locus; the arcs outside it delimit the 22 maximal clades that consist of carriers only, the

largest of which contains 12 genomes together with bja295, and the number beside an arc gives the size of the clade where it exceeds two. The outer ring gives the state of the syntenic site of the locus in each genome, classified as in Section 2.6. The composition of the panel and the inference are described in Section 3.10, and the carriage pattern is discussed in Section 2.7.

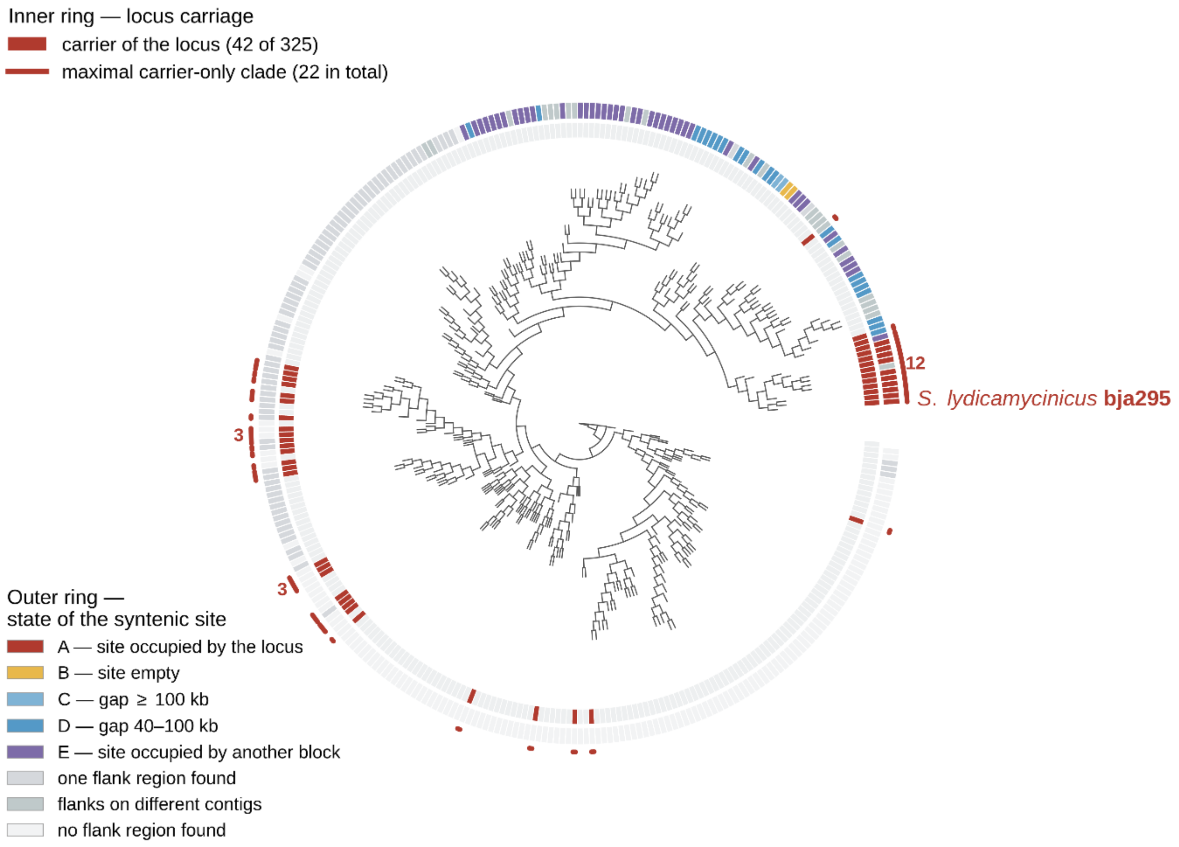

Table S1: Utilization of carbon sources by strain bja295 on ISP9 basal medium. The assay was performed in three independent replicates; the reading is qualitative, scored as growth or no growth, and the three replicates agreed on every substrate, so no measure of dispersion is given.

Carbon source	bja295	Carbon source	bja295
Adonitol	+	Mannose	+
L-Arabinose	+	Melibiose	+
Galactose	+	Rhamnose	+
Glucose	+	Raffinose	+
Dulcitol	+	Salicin	+
Inulin	+	Sucrose	+

Xylose	+	Sorbitol	+
Lactose	+	Trehalose	+
Maltose	+	Fructose	+
Mannitol	+	Cellobiose	+
L-Lysine	+	L-Ornithine	+

Table S2: Antibiotic susceptibility of strain bja295 (disc diffusion). Each disc was assayed in three independent biological replicates; values are the mean ± standard deviation of the three measurements, with the individual readings given in parentheses. Diameters were read to the nearest millimetre. A dash marks the absence of a detectable zone. No statistical comparison between discs was performed: the assay was run to place the strain within the described phenotypic variation of the species and to provide a reference for the resistance determinants identified in the genome, not to compare agents.

Antibiotic (disc content)	Class	Cellular target	Zone diameter, mm
Levofloxacin (Le 5)	Fluoroquinolones	DNA gyrase	16.3 ± 0.6 (16, 16, 17)
Ciprofloxacin (CIP 5)	Fluoroquinolones	DNA gyrase	19.0 ± 1.0 (18, 19, 20)
Norfloxacin (NOR 10)	Fluoroquinolones	DNA gyrase	–
Oxacillin (Ox 5)	β-Lactams	Cell wall synthesis	–
Tazobactam (TPZ 36)	β-Lactams	Cell wall synthesis	–
Tazobactam (TPZ 110)	β-Lactams	Cell wall synthesis	12.7 ± 0.6 (12, 13, 13)
Amoxicillin–clavulanic acid (AMC 30)	β-Lactams	Cell wall synthesis	–
Cefepime (FEP 30)	β-Lactams	Cell wall synthesis	–
Ceftazidime (CAZ 30)	Cephalosporins	Cell wall synthesis	–
Cefoxitin (FOX 30)	Cephalosporins	Cell wall synthesis	18.0 ± 0.0 (18, 18, 18)

Trimethoprim–sulfamethoxazole (SXT 25)	Sulfonamides	Nucleic acid synthesis	39.7 ± 1.5 (38, 40, 41)
Trimethoprim (TMP 25)	Sulfonamides	Nucleic acid synthesis	–
Vancomycin (Va 5)	Glycopeptides	Cell wall synthesis	27.7 ± 0.6 (27, 28, 28)
Erythromycin (E 15)	Macrolides	50S, peptidyl transferase centre	35.0 ± 1.0 (34, 35, 36)
Azithromycin (ATM 30)	Macrolides	50S, peptidyl transferase centre	40.3 ± 1.5 (39, 40, 42)
Tobramycin (TOB 10)	Aminoglycosides	30S, A site	28.7 ± 0.6 (28, 29, 29)
Amikacin (AK 30)	Aminoglycosides	30S, A site	40.3 ± 0.6 (40, 40, 41)
Streptomycin (S 50)	Aminoglycosides	30S, A site	–
Streptothricin F (500)	Streptothricins	30S	–
Gentamicin (CN 10)	Aminoglycosides	30S, A site	24.0 ± 1.0 (23, 24, 25)
Clindamycin (Da 2)	Lincosamides	50S	28.7 ± 1.5 (27, 29, 30)
Tigecycline (TGC 15)	Glycylcyclines	30S, A site	16.0 ± 0.0 (16, 16, 16)

Table S3: Digital DNA–DNA hybridisation values over the 46-genome panel of the *S. lydicamycinicus* complex.

Genome	NPDC panel	Genomospecies	Species	dDDH to *lydicamycinicus* type, %	dDDH to *platensis* type, %
CW25	—	I	*S. lydicamycinicus*	95.5	59.4
MMS22-DDSA8	—	I	*S. lydicamycinicus*	95.1	59.3

NPDC001531	lyd	I	*S. lydicamycinicus*	95.5	59.6
NPDC001772	lyd	I	*S. lydicamycinicus*	95.2	59.5
NPDC006927	lyd	I	*S. lydicamycinicus*	95.0	59.4
NPDC008293	lyd	I	*S. lydicamycinicus*	95.2	59.5
NPDC020847	lyd	I	*S. lydicamycinicus*	95.6	59.4
NPDC023103	lyd	I	*S. lydicamycinicus*	95.2	59.4
NPDC023112	lyd	I	*S. lydicamycinicus*	92.8	59.6
NPDC024095	lyd	I	*S. lydicamycinicus*	85.8	59.5
NPDC024428	lyd	I	*S. lydicamycinicus*	94.8	59.4
NPDC025141	lyd	I	*S. lydicamycinicus*	94.9	59.4
NPDC025511	lyd	I	*S. lydicamycinicus*	95.3	59.5
NPDC029235	lyd	I	*S. lydicamycinicus*	92.4	59.6
NPDC030177	lyd	I	*S. lydicamycinicus*	95.4	59.6
NPDC030806	lyd	I	*S. lydicamycinicus*	95.7	59.6
NPDC032166	lyd	I	*S. lydicamycinicus*	94.9	59.5
NPDC032947	lyd	I	*S. lydicamycinicus*	92.4	59.5

NPDC033460	lyd	I	*S. lydicamycinicus*	92.1	59.4
NPDC033730	lyd	I	*S. lydicamycinicus*	92.0	59.5
NPDC035741	lyd	I	*S. lydicamycinicus*	92.0	59.3
NPDC039428	lyd	I	*S. lydicamycinicus*	92.3	59.4
NPDC088830	lyd	I	*S. lydicamycinicus*	94.9	59.4
TP-A0598	—	I	*S. lydicamycinicus*	100.0	59.5
NPDC025256	plat	II	*S. platensis*	59.8	85.9
NPDC025659	plat	II	*S. platensis*	59.7	88.5
NPDC032794	plat	II	*S. platensis*	60.0	86.0
NPDC032847	plat	II	*S. platensis*	60.1	85.7
NPDC034584	plat	II	*S. platensis*	60.0	85.5
NPDC042329	plat	II	*S. platensis*	60.0	85.7
NPDC044766	plat	II	*S. platensis*	60.0	85.6
NPDC046625	plat	II	*S. platensis*	60.2	85.7
NPDC033267	lyd	III	—	59.1	56.9
NPDC057006	lyd	III	—	59.4	56.9
NPDC101287	lyd	III	—	59.1	57.0
NPDC127097	lyd	III	—	59.5	57.0
NPDC002484	lyd	IV	—	62.6	60.5
NPDC020741	lyd	IV	—	62.7	60.5
NPDC020760	—	IV	—	62.7	60.5
NPDC041037	plat	V	—	58.5	69.1

NPDC046888	plat	V	—	57.9	67.6
NPDC047015	plat	V	—	58.0	67.5
NPDC029498	plat	VI	—	57.8	60.2
NPDC052111	plat	VI	—	58.1	60.1
NPDC029466	lyd	VII	—	68.8	57.5
NPDC033176	lyd	VIII	—	64.8	61.8

Table S4: Inventory of the biosynthetic gene clusters of strain bja295 predicted by antiSMASH.

Contig	Region	Start	End	Product class	Closest known cluster	MIBiG	Similar genes
chromosome	1	441,487	491,126	T3PKS; lanthipeptide-class-iii	SapB	BGC0000551.5	4
chromosome	2	578,892	615,002	NAPAA	stenothricin	BGC0000431.5	3
chromosome	3	631,432	656,856	RiPP-like; terpene	hexacosalactone A	BGC0002497.3	3
chromosome	4	684,463	756,978	T2PKS	spore pigment	BGC0000271.5	10
chromosome	5	759,435	905,017	NRPS; T1PKS; melanin	lydicamycin	BGC0001477.5	24
chromosome	6	1,045,029	1,077,922	NI-siderophore	peucechelin	BGC0002466.3	5
chromosome	7	1,709,897	1,739,714	NI-siderophore	legonoxamine A/desferrioxamine B/legonoxamine B	BGC0002305.2	6
chromosome	8	1,811,250	1,821,666	ectoine	ectoine	BGC0002052.3	4
chromosome	9	2,732,420	2,778,761	T1PKS	skyllamycin D/skyllamycin E	BGC0002676.2	6
chromosome	10	2,833,820	2,854,881	terpene	ebelactone	BGC0001580.3	2
chromosome	11	3,242,378	3,286,787	NRPS	—	—	—
chromosome	12	4,234,992	4,257,226	terpene	salinomycin	BGC0000144.5	3
chromosome	13	4,384,550	4,457,752	NRPS; lanthipeptide-class-iii; lassopeptide	glycinocin A	BGC0000379.5	7

chromosome	14	4,985,610	5,006,668	atropopeptide	dudomycin A	BGC0002359.3	11
chromosome	15	5,384,631	5,406,163	triceptide	—	—	—
chromosome	16	5,964,888	5,997,594	NI-siderophore	schizokinen	BGC0002683.2	2
chromosome	17	6,076,855	6,099,467	lassopeptide	citrulassin D	BGC0001550.3	5
chromosome	18	6,129,019	6,149,903	terpene-precursor	FD-594	BGC0000222.5	4
chromosome	19	6,158,554	6,169,534	butyrolactone	—	—	—
chromosome	20	6,221,449	6,232,291	RiPP-like	14-hydroxyisochainin	BGC0002788.2	5
chromosome	21	6,415,335	6,469,155	NRPS; NRPS-like; T1PKS	incarnatapeptin A/incarnatapeptin B/dentigerumycin F/dentigerumycin G	BGC0002735.2	4
chromosome	22	6,841,265	6,867,883	terpene	hopene	BGC0000663.5	8
chromosome	23	6,978,150	7,030,540	T1PKS; hglE-KS	hexacosalactone A	BGC0002497.3	3
chromosome	24	7,078,336	7,150,890	T2PKS	oxytetracycline	BGC0000254.5	19
chromosome	25	7,156,798	7,181,347	lanthipeptide-class-i	—	—	—
chromosome	26	7,257,490	7,269,424	RiPP-like	—	—	—
chromosome	27	7,495,660	7,506,631	butyrolactone	coelimycin P1	BGC0000038.5	3
chromosome	28	7,568,131	7,625,844	NRPS; NRPS-like; other	antipain	BGC0002051.2	6
chromosome	29	7,636,590	7,665,005	terpene	isorenieratene	BGC0000664.5	7
chromosome	30	7,776,709	7,789,604	hydrogen-cyanide	aborycin	BGC0002285.2	3
chromosome	31	7,841,393	7,864,697	terpene	pulvomycin B/pulvomycin C/pulvomycin D	BGC0002374.2	2
chromosome	32	7,892,732	7,903,652	butyrolactone	—	—	—
contig 2	1	25,926	59,837	NAPAA	ε-Poly-L-lysine	BGC0002174.2	1

Table S5: The 137 carrier assemblies of the oxytetracycline locus. Assemblies from NCBI (125) were scored as carriers when all three probe proteins lay on one contig within 40 kb; genomes of the NPDC collection without an INSDC accession (12) were scored when that condition was met or when each of the three proteins reached at least 60 % identity, the column "Criterion met" giving which of the two applies to each row. For rows accepted on identity the locus is split between contigs, and the contig and span refer to the contig carrying the larger part of the 24-gene locus. The column "Table 1 row" gives the group of Table 1 to which each assembly belongs. Species names for NPDC genomes are taken from the collection portal rather than from the headers of the downloaded files, which are outdated.

Accession	Three genes	Colocalised	Identity >= 60 %	Contig	Span, bp	OtcR (06232)	OxyA (06235)	OtrA (06254)	Species as deposited	Source	Criterion met	Table 1 row (ANI group)	Note
GCF_038250085.1	1	1	1	NZ_CP138630.1	25622	73.6/99	88.2/99	75.0/100	Mutant Streptomyces	NCBI	colocalisation	Streptomyces rimosus	Organism name in NCBI is literally "Mutant Streptomyces"; R6-500 lineage, ANI group S. rimosus.
GCF_038252105.1	1	1	1	NZ_CP138628.1	25621	73.6/99	88.2/99	75.0/100	Mutant Streptomyces	NCBI	colocalisation	Streptomyces rimosus	Organism name in NCBI is literally "Mutant Streptomyces"; R6-500 lineage, ANI group S. rimosus.
GCF_055570715.1	1	1	1	NZ_JBVIGR010000004.1	25704	72.4/99	88.2/99	75.0/100	Streptomyces albofaciens	NCBI	colocalisation	(below the species threshold)	
GCF_055572615.1	1	1	1	NZ_JBVIDA010000001.1	25710	73.2/99	88.2/99	74.7/100	Streptomyces albofaciens	NCBI	colocalisation	Streptomyces albofaciens	
GCF_055583965.1	1	1	1	NZ_JBVJMY010000011.1	21150	67.6/99	85.8/99	72.6/100	Streptomyces corynorhini	NCBI	colocalisation	Streptomyces corynorhini	
GCF_055584005.1	1	1	1	NZ_JBVJMX010000012.1	21150	67.6/99	85.8/99	72.6/100	Streptomyces corynorhini	NCBI	colocalisation	Streptomyces corynorhini	
GCA_055566155.1	1	1	1	JBVHSX010000010.1	24608	70.4/99	86.1/100	72.9/100	Streptomyces halstedii	NCBI	colocalisation	Streptomyces griseolus / Streptomyces halstedii	
GCF_040301765.1	1	1	1	NZ_JBEPGE010000007.1	24608	70.4/99	86.1/100	72.9/100	Streptomyces halstedii	NCBI	colocalisation	Streptomyces griseolus / Streptomyces halstedii	
GCF_044330965.1	1	1	1	NZ_JBIRMF010000001.1	24607	70.4/99	86.1/100	72.9/100	Streptomyces halstedii	NCBI	colocalisation	Streptomyces griseolus / Streptomyces halstedii	
GCF_000829715.2	1	1	1	NZ_BBNO01000007.1	25262	100.0/100	99.5/100	99.5/100	Streptomyces lydicamycinicus	NCBI	colocalisation	Streptomyces lydicamycinicus	
GCF_023702105.1	1	1	1	NZ_CP098437.1	25665	100.0/100	99.1/100	99.7/59	Streptomyces lydicamycinicus	NCBI	colocalisation	Streptomyces lydicamycinicus	
GCF_049538515.1	1	1	1	NZ_JBMUZZ010000002.1	25264	100.0/100	99.3/100	99.5/100	Streptomyces lydicamycinicus	NCBI	colocalisation	Streptomyces lydicamycinicus	
GCF_049538975.1	1	1	1	NZ_JBMVAV010000006.1	25261	100.0/100	99.5/100	99.5/100	Streptomyces lydicamycinicus	NCBI	colocalisation	Streptomyces lydicamycinicus	
GCF_049540535.1	1	1	1	NZ_JBMVCO010000001.1	25264	100.0/100	99.5/100	99.5/100	Streptomyces lydicamycinicus	NCBI	colocalisation	Streptomyces lydicamycinicus	
GCF_049543175.1	1	1	1	NZ_JBMVEF010000007.1	25262	100.0/100	99.5/100	99.5/100	Streptomyces lydicamycinicus	NCBI	colocalisation	Streptomyces lydicamycinicus	
GCF_053596115.1	1	1	1	NZ_JBSFFM010000001.1	25265	100.0/100	99.5/100	100.0/100	Streptomyces lydicamycinicus	NCBI	colocalisation	Streptomyces lydicamycinicus	
GCF_055664545.1	1	1	1	NZ_JBVOPC010000007.1	25422	100.0/100	99.5/100	99.5/100	Streptomyces lydicamycinicus	NCBI	colocalisation	Streptomyces lydicamycinicus	
GCF_055664925.1	1	1	1	NZ_JBVORA010000001.1	25261	100.0/100	99.5/100	99.5/100	Streptomyces lydicamycinicus	NCBI	colocalisation	Streptomyces lydicamycinicus	

GCF_055665105.1	1	1	1	NZ_JBVOQR010000001.1	25263	100.0/100	99.3/100	99.7/100	Streptomyces lydicamycinicus	NCBI	colocalisation	Streptomyces lydicamycinicus	
GCF_055668245.1	1	1	1	NZ_JBVOPK010000012.1	25264	100.0/100	99.3/100	99.8/100	Streptomyces lydicamycinicus	NCBI	colocalisation	Streptomyces lydicamycinicus	
GCF_055668365.1	1	1	1	NZ_JBVOPE010000008.1	25248	100.0/100	99.5/100	99.7/100	Streptomyces lydicamycinicus	NCBI	colocalisation	Streptomyces lydicamycinicus	
GCF_000725565.1	1	1	1	NZ_JOCK01000001.1	25615	73.6/99	88.2/99	74.9/100	Streptomyces peucetius	NCBI	colocalisation	Streptomyces rimosus	Deposited as S. peucetius; ANI 98.0 % to S. rimosus - the NCBI label is wrong (this is the RefSeq reference genome of the species).
GCF_008704855.1	1	1	1	NZ_CP023691.1	25449	83.3/100	92.7/100	87.5/100	Streptomyces platensis	NCBI	colocalisation	Streptomyces glebosus / Streptomyces libani / Streptomyces platensis	
GCF_049541835.1	1	1	1	NZ_JBMVDN010000004.1	25677	83.3/100	92.5/100	87.7/100	Streptomyces platensis	NCBI	colocalisation	(below the species threshold)	
GCF_000331185.2	1	1	1	NZ_CP048261.1	25621	73.6/99	88.2/99	75.0/100	Streptomyces rimosus	NCBI	colocalisation	Streptomyces rimosus	
GCF_000707925.2	1	1	1	NZ_CP045803.1	25621	73.6/99	88.2/99	75.0/100	Streptomyces rimosus	NCBI	colocalisation	Streptomyces rimosus	
GCF_006229535.1	1	1	1	NZ_CP025551.1	25622	73.6/99	88.2/99	75.0/100	Streptomyces rimosus	NCBI	colocalisation	Streptomyces rimosus	
GCF_008704655.1	1	1	1	NZ_CP023688.1	25622	73.6/99	88.2/99	75.0/100	Streptomyces rimosus	NCBI	colocalisation	Streptomyces rimosus	
GCF_017751265.1	1	1	1	NZ_CP043497.1	25622	73.6/99	88.2/99	75.0/100	Streptomyces rimosus	NCBI	colocalisation	Streptomyces rimosus	
GCF_022760195.1	1	1	1	NZ_CP094298.1	25622	73.6/99	88.2/99	75.0/100	Streptomyces rimosus	NCBI	colocalisation	Streptomyces rimosus	
GCF_024218735.1	1	1	1	NZ_CP100397.1	25622	73.6/99	88.2/99	75.0/100	Streptomyces rimosus	NCBI	colocalisation	Streptomyces rimosus	
GCF_039581505.1	1	1	1	NZ_JBCPSI010000001.1	25620	73.6/99	88.2/99	74.7/100	Streptomyces rimosus	NCBI	colocalisation	Streptomyces rimosus	
GCF_040302265.1	1	1	1	NZ_JBEORM010000016.1	25622	73.6/99	88.2/99	75.0/100	Streptomyces rimosus	NCBI	colocalisation	Streptomyces rimosus	
GCF_040631695.1	1	1	1	NZ_JBEYAK010000026.1	25622	73.6/99	88.2/99	75.0/100	Streptomyces rimosus	NCBI	colocalisation	Streptomyces rimosus	
GCF_040631895.1	1	1	1	NZ_JBEYAY010000030.1	25621	73.6/99	88.2/99	75.0/100	Streptomyces rimosus	NCBI	colocalisation	Streptomyces rimosus	
GCF_040632885.1	1	1	1	NZ_JBEYCT010000048.1	25622	73.6/99	88.2/99	75.0/100	Streptomyces rimosus	NCBI	colocalisation	Streptomyces rimosus	
GCF_040636605.1	1	1	1	NZ_JBEZUW010000023.1	25622	73.6/99	88.2/99	75.0/100	Streptomyces rimosus	NCBI	colocalisation	Streptomyces rimosus	
GCF_040640435.1	1	1	1	NZ_JBFABT010000028.1	25622	73.6/99	88.2/99	75.0/100	Streptomyces rimosus	NCBI	colocalisation	Streptomyces rimosus	
GCF_040640455.1	1	1	1	NZ_JBFABU010000024.1	25621	73.6/99	88.2/99	75.0/100	Streptomyces rimosus	NCBI	colocalisation	Streptomyces rimosus	
GCF_040640495.1	1	1	1	NZ_JBFABV010000024.1	25622	73.6/99	88.2/99	75.0/100	Streptomyces rimosus	NCBI	colocalisation	Streptomyces rimosus	
GCF_040640515.1	1	1	1	NZ_JBFABW010000021.1	25621	73.6/99	88.2/99	75.0/100	Streptomyces rimosus	NCBI	colocalisation	Streptomyces rimosus	
GCF_040646795.1	1	1	1	NZ_JBEZAX010000004.1	25621	73.6/99	88.2/99	74.7/100	Streptomyces rimosus	NCBI	colocalisation	Streptomyces rimosus	

GCF_040703375.1	1	1	1	NZ_JBEYCS010000026.1	25621	73.6/99	88.2/99	75.0/100	Streptomyces rimosus	NCBI	colocalisation	Streptomyces rimosus	
GCF_043075755.1	1	1	1	NZ_JBIBQT010000016.1	25621	73.6/99	88.2/99	75.0/100	Streptomyces rimosus	NCBI	colocalisation	Streptomyces rimosus	
GCF_043075795.1	1	1	1	NZ_JBIBQX010000017.1	25622	73.6/99	88.2/99	75.0/100	Streptomyces rimosus	NCBI	colocalisation	Streptomyces rimosus	
GCF_043075855.1	1	1	1	NZ_JBIBQV010000016.1	25622	73.6/99	88.2/99	75.0/100	Streptomyces rimosus	NCBI	colocalisation	Streptomyces rimosus	
GCF_043075955.1	1	1	1	NZ_JBIBRC010000012.1	25622	73.6/99	88.2/99	75.0/100	Streptomyces rimosus	NCBI	colocalisation	Streptomyces rimosus	
GCF_043075985.1	1	1	1	NZ_JBIBRA010000012.1	25622	73.6/99	88.2/99	75.0/100	Streptomyces rimosus	NCBI	colocalisation	Streptomyces rimosus	
GCF_043076015.1	1	1	1	NZ_JBIBQZ010000012.1	25621	73.6/99	88.2/99	75.0/100	Streptomyces rimosus	NCBI	colocalisation	Streptomyces rimosus	
GCF_043076035.1	1	1	1	NZ_JBIBQY010000016.1	25622	73.6/99	88.2/99	75.0/100	Streptomyces rimosus	NCBI	colocalisation	Streptomyces rimosus	
GCF_043076055.1	1	1	1	NZ_JBIBRB010000010.1	25621	73.6/99	88.2/99	75.0/100	Streptomyces rimosus	NCBI	colocalisation	Streptomyces rimosus	
GCF_043076155.1	1	1	1	NZ_JBIBRF010000025.1	25622	73.6/99	88.2/99	75.0/100	Streptomyces rimosus	NCBI	colocalisation	Streptomyces rimosus	
GCF_043076175.1	1	1	1	NZ_JBIBRD010000024.1	25621	73.6/99	88.2/99	75.0/100	Streptomyces rimosus	NCBI	colocalisation	Streptomyces rimosus	
GCF_043076195.1	1	1	1	NZ_JBIBRE010000015.1	25622	73.6/99	88.2/99	75.0/100	Streptomyces rimosus	NCBI	colocalisation	Streptomyces rimosus	
GCF_043076375.1	1	1	1	NZ_JBIBRJ010000017.1	25622	73.6/99	88.2/99	75.0/100	Streptomyces rimosus	NCBI	colocalisation	Streptomyces rimosus	
GCF_043076435.1	1	1	1	NZ_JBIBRK010000013.1	25621	73.6/99	88.2/99	75.0/100	Streptomyces rimosus	NCBI	colocalisation	Streptomyces rimosus	
GCF_043076475.1	1	1	1	NZ_JBIBRN010000016.1	25622	73.6/99	88.2/99	75.0/100	Streptomyces rimosus	NCBI	colocalisation	Streptomyces rimosus	
GCF_043076535.1	1	1	1	NZ_JBIBRR010000013.1	25621	73.6/99	88.2/99	75.0/100	Streptomyces rimosus	NCBI	colocalisation	Streptomyces rimosus	
GCF_043076655.1	1	1	1	NZ_JBIBRS010000011.1	25621	73.6/99	88.2/99	75.0/100	Streptomyces rimosus	NCBI	colocalisation	Streptomyces rimosus	
GCF_043076815.1	1	1	1	NZ_JBIBRW010000011.1	25621	73.6/99	88.2/99	75.0/100	Streptomyces rimosus	NCBI	colocalisation	Streptomyces rimosus	
GCF_043076915.1	1	1	1	NZ_JBIBRY010000016.1	25622	73.6/99	88.2/99	75.0/100	Streptomyces rimosus	NCBI	colocalisation	Streptomyces rimosus	
GCF_043077005.1	1	1	1	NZ_JBIBSA010000020.1	25621	73.6/99	88.2/99	75.0/100	Streptomyces rimosus	NCBI	colocalisation	Streptomyces rimosus	
GCF_043079505.1	1	1	1	NZ_JBIBUP010000025.1	25622	73.6/99	88.2/99	75.0/100	Streptomyces rimosus	NCBI	colocalisation	Streptomyces rimosus	
GCF_043079565.1	1	1	1	NZ_JBIBUQ010000023.1	25621	73.6/99	88.2/99	75.0/100	Streptomyces rimosus	NCBI	colocalisation	Streptomyces rimosus	
GCF_043080145.1	1	1	1	NZ_JBIBVG010000013.1	25622	73.6/99	88.2/99	75.0/100	Streptomyces rimosus	NCBI	colocalisation	Streptomyces rimosus	
GCF_043080595.1	1	1	1	NZ_JBIBVR010000015.1	25621	73.6/99	88.2/99	75.0/100	Streptomyces rimosus	NCBI	colocalisation	Streptomyces rimosus	
GCF_043088805.1	1	1	1	NZ_JBICAG010000012.1	25622	73.6/99	88.2/99	75.0/100	Streptomyces rimosus	NCBI	colocalisation	Streptomyces rimosus	
GCF_044327725.1	1	1	1	NZ_JBIRGA010000001.1	25613	73.6/99	88.4/99	74.9/100	Streptomyces rimosus	NCBI	colocalisation	Streptomyces rimosus	
GCF_044328145.1	1	1	1	NZ_JBIRGT010000019.1	25621	73.6/99	88.2/99	75.0/100	Streptomyces rimosus	NCBI	colocalisation	Streptomyces rimosus	
GCF_044328715.1	1	1	1	NZ_JBIRHW010000002.1	25633	73.6/99	88.2/99	74.3/100	Streptomyces rimosus	NCBI	colocalisation	Streptomyces rimosus	
GCF_044328745.1	1	1	1	NZ_JBIRHX010000009.1	25634	73.6/99	88.2/99	74.3/100	Streptomyces rimosus	NCBI	colocalisation	Streptomyces rimosus	

GCF_044328905.1	1	1	1	NZ_JBIRIC010000038.1	25621	73.6/99	88.2/99	75.0/100	Streptomyces rimosus	NCBI	colocalisation	Streptomyces rimosus	
GCF_044329005.1	1	1	1	NZ_JBIRIL010000021.1	25622	73.6/99	88.2/99	75.0/100	Streptomyces rimosus	NCBI	colocalisation	Streptomyces rimosus	
GCF_044329065.1	1	1	1	NZ_JBIRIN010000012.1	25621	73.6/99	88.2/99	75.0/100	Streptomyces rimosus	NCBI	colocalisation	Streptomyces rimosus	
GCF_044329105.1	1	1	1	NZ_JBIRIQ010000022.1	25621	73.6/99	88.2/99	75.0/100	Streptomyces rimosus	NCBI	colocalisation	Streptomyces rimosus	
GCF_044329135.1	1	1	1	NZ_JBIRIR010000023.1	25621	73.6/99	88.2/99	75.0/100	Streptomyces rimosus	NCBI	colocalisation	Streptomyces rimosus	
GCF_044329175.1	1	1	1	NZ_JBIRIT010000023.1	25622	73.6/99	88.2/99	75.0/100	Streptomyces rimosus	NCBI	colocalisation	Streptomyces rimosus	
GCF_044329205.1	1	1	1	NZ_JBIRIU010000026.1	25621	73.6/99	88.2/99	75.0/100	Streptomyces rimosus	NCBI	colocalisation	Streptomyces rimosus	
GCF_044329225.1	1	1	1	NZ_JBIRIW010000024.1	25621	73.6/99	88.2/99	75.0/100	Streptomyces rimosus	NCBI	colocalisation	Streptomyces rimosus	
GCF_044329235.1	1	1	1	NZ_JBIRIV010000010.1	25621	73.6/99	88.2/99	75.0/100	Streptomyces rimosus	NCBI	colocalisation	Streptomyces rimosus	
GCF_044329265.1	1	1	1	NZ_JBIRIX010000013.1	25622	73.6/99	88.2/99	75.0/100	Streptomyces rimosus	NCBI	colocalisation	Streptomyces rimosus	
GCF_044329845.1	1	1	1	NZ_JBIRKA010000021.1	25621	73.6/99	88.2/99	75.0/100	Streptomyces rimosus	NCBI	colocalisation	Streptomyces rimosus	
GCF_044329945.1	1	1	1	NZ_JBIRKF010000014.1	25621	73.6/99	88.2/99	75.0/100	Streptomyces rimosus	NCBI	colocalisation	Streptomyces rimosus	
GCF_044330115.1	1	1	1	NZ_JBIRKO010000022.1	25622	73.6/99	88.2/99	75.0/100	Streptomyces rimosus	NCBI	colocalisation	Streptomyces rimosus	
GCF_044330135.1	1	1	1	NZ_JBIRKP010000019.1	25622	73.6/99	88.2/99	75.0/100	Streptomyces rimosus	NCBI	colocalisation	Streptomyces rimosus	
GCF_044330235.1	1	1	1	NZ_JBIRKV010000009.1	25621	73.6/99	88.2/99	75.0/100	Streptomyces rimosus	NCBI	colocalisation	Streptomyces rimosus	
GCF_044330245.1	1	1	1	NZ_JBIRKW010000022.1	25622	73.6/99	88.2/99	75.0/100	Streptomyces rimosus	NCBI	colocalisation	Streptomyces rimosus	
GCF_044330275.1	1	1	1	NZ_JBIRKX010000011.1	25622	73.6/99	88.2/99	75.0/100	Streptomyces rimosus	NCBI	colocalisation	Streptomyces rimosus	
GCF_044330325.1	1	1	1	NZ_JBIRKY010000010.1	25621	73.6/99	88.2/99	75.0/100	Streptomyces rimosus	NCBI	colocalisation	Streptomyces rimosus	
GCF_044330345.1	1	1	1	NZ_JBIRKZ010000013.1	25621	73.6/99	88.2/99	75.0/100	Streptomyces rimosus	NCBI	colocalisation	Streptomyces rimosus	
GCF_044330355.1	1	1	1	NZ_JBIRLB010000013.1	25622	73.6/99	88.2/99	75.0/100	Streptomyces rimosus	NCBI	colocalisation	Streptomyces rimosus	
GCF_044330365.1	1	1	1	NZ_JBIRLC010000029.1	25622	73.6/99	88.2/99	75.0/100	Streptomyces rimosus	NCBI	colocalisation	Streptomyces rimosus	
GCF_044330375.1	1	1	1	NZ_JBIRLA010000011.1	25622	73.6/99	88.2/99	75.0/100	Streptomyces rimosus	NCBI	colocalisation	Streptomyces rimosus	
GCF_044330425.1	1	1	1	NZ_JBIRLD010000017.1	25622	73.6/99	88.2/99	75.0/100	Streptomyces rimosus	NCBI	colocalisation	Streptomyces rimosus	
GCF_044330615.1	1	1	1	NZ_JBIRLN010000023.1	25621	73.6/99	88.2/99	75.0/100	Streptomyces rimosus	NCBI	colocalisation	Streptomyces rimosus	
GCF_044330645.1	1	1	1	NZ_JBIRLO010000022.1	25621	73.6/99	88.2/99	75.0/100	Streptomyces rimosus	NCBI	colocalisation	Streptomyces rimosus	
GCF_044332575.1	1	1	1	NZ_JBIRPJ010000005.1	25622	73.6/99	88.2/99	75.0/100	Streptomyces rimosus	NCBI	colocalisation	Streptomyces rimosus	
GCF_044332605.1	1	1	1	NZ_JBIRPK010000013.1	25622	73.6/99	88.2/99	75.0/100	Streptomyces rimosus	NCBI	colocalisation	Streptomyces rimosus	
GCF_044332665.1	1	1	1	NZ_JBIRPN010000017.1	25622	73.6/99	88.2/99	75.0/100	Streptomyces rimosus	NCBI	colocalisation	Streptomyces rimosus	
GCF_044332685.1	1	1	1	NZ_JBIRPO010000009.1	25621	73.6/99	88.2/99	75.0/100	Streptomyces rimosus	NCBI	colocalisation	Streptomyces rimosus	

GCF_044332695.1	1	1	1	NZ_JBIRPP010000016.1	25621	73.6/99	88.2/99	75.0/100	Streptomyces rimosus	NCBI	colocalisation	Streptomyces rimosus	
GCF_044332705.1	1	1	1	NZ_JBIRPL010000013.1	25621	73.6/99	88.2/99	75.0/100	Streptomyces rimosus	NCBI	colocalisation	Streptomyces rimosus	
GCF_044332785.1	1	1	1	NZ_JBIRPQ010000015.1	25622	73.6/99	88.2/99	75.0/100	Streptomyces rimosus	NCBI	colocalisation	Streptomyces rimosus	
GCF_044332795.1	1	1	1	NZ_JBIRPS010000026.1	25622	73.6/99	88.2/99	75.0/100	Streptomyces rimosus	NCBI	colocalisation	Streptomyces rimosus	
GCF_044332815.1	1	1	1	NZ_JBIRPR010000018.1	25622	73.6/99	88.2/99	75.0/100	Streptomyces rimosus	NCBI	colocalisation	Streptomyces rimosus	
GCF_044390975.1	1	1	1	NZ_JBIRZR010000025.1	25621	73.6/99	88.2/99	75.0/100	Streptomyces rimosus	NCBI	colocalisation	Streptomyces rimosus	
GCF_044391155.1	1	1	1	NZ_JBIRZW010000019.1	25622	73.6/99	88.2/99	75.0/100	Streptomyces rimosus	NCBI	colocalisation	Streptomyces rimosus	
GCF_044391195.1	1	1	1	NZ_JBIRZU010000015.1	25621	73.6/99	88.2/99	75.0/100	Streptomyces rimosus	NCBI	colocalisation	Streptomyces rimosus	
GCF_044546885.1	1	1	1	NZ_JBITZO010000001.1	25621	73.6/99	88.2/99	74.9/100	Streptomyces rimosus	NCBI	colocalisation	Streptomyces rimosus	
GCF_049539135.1	1	1	1	NZ_JBMVBC010000012.1	25621	73.6/99	88.2/99	75.0/100	Streptomyces rimosus	NCBI	colocalisation	Streptomyces rimosus	
GCF_055562835.1	1	1	1	NZ_JBVHEN010000015.1	25622	73.6/99	88.2/99	75.0/100	Streptomyces rimosus	NCBI	colocalisation	Streptomyces rimosus	
GCF_040634485.1	1	1	1	NZ_JBEYFQ010000020.1	25613	72.8/99	87.0/99	74.8/99	Streptomyces sp.	NCBI	colocalisation	(below the species threshold)	
GCF_040654875.1	1	1	1	NZ_JBFAIV010000007.1	25261	70.3/99	82.8/99	69.9/99	Streptomyces sp.	NCBI	colocalisation	(below the species threshold)	
GCF_044327805.1	1	1	1	NZ_JBIRGD010000023.1	25669	73.6/99	87.9/99	74.7/99	Streptomyces sp.	NCBI	colocalisation	(below the species threshold)	
GCF_044329785.1	1	1	1	NZ_JBIRJY010000024.1	25603	73.2/99	87.0/99	75.1/99	Streptomyces sp.	NCBI	colocalisation	(below the species threshold)	
GCF_044332485.1	1	1	1	NZ_JBIRPD010000031.1	25586	72.8/99	87.2/99	74.8/99	Streptomyces sp.	NCBI	colocalisation	(below the species threshold)	
GCF_055574515.1	1	1	1	NZ_JBVHZH010000027.1	25125	97.2/100	98.3/99	96.1/100	Streptomyces sp.	NCBI	colocalisation	(below the species threshold)	Deposited as Streptomyces sp.; same strain as NPDC029466; ANI 96.8 % to the type strain of S. lydicamycinicus, counted in the S. lydicamycinicus group of Table 1.
GCF_055595265.1	1	1	1	NZ_JBVIVV010000018.1	24607	72.0/99	87.2/99	74.6/100	Streptomyces sp.	NCBI	colocalisation	(below the species threshold)	
GCF_055664445.1	1	1	1	NZ_JBVOOS010000020.1	25653	73.6/99	87.9/99	74.5/99	Streptomyces sp.	NCBI	colocalisation	(below the species threshold)	
GCF_056434425.1	1	1	1	NZ_JBWKFF010000023.1	25530	70.0/99	82.7/99	68.9/99	Streptomyces sp.	NCBI	colocalisation	(below the species threshold)	
GCF_040646475.1	1	1	1	NZ_JBEZAH010000003.1	26056	71.1/99	83.3/99	69.6/99	Streptomyces varsoviensis	NCBI	colocalisation	Streptomyces varsoviensis	
GCF_056430105.1	1	1	1	NZ_JBWKLN010000004.1	26260	71.1/99	83.1/99	69.6/99	Streptomyces varsoviensis	NCBI	colocalisation	Streptomyces varsoviensis	
GCF_056827125.1	1	1	1	NZ_CP162614.1	27456	71.1/99	83.1/99	69.6/99	Streptomyces varsoviensis	NCBI	colocalisation	Streptomyces varsoviensis	

GCA_001888885.2	1	1	0	CP090841.1	23598	45.2/99	80.0/97	56.6/99	Streptomyces viridifaciens	NCBI	colocalisation	Streptomyces viridifaciens	
GCF_008704475.1	1	1	0	NZ_CP023698.1	23598	45.2/99	80.0/97	56.6/99	Streptomyces viridifaciens	NCBI	colocalisation	Streptomyces aureofaciens	
NPDC001531	1	0	1	NPDC001531.ctg-0048	23495	100.0/100	99.5/100	99.8/100	Streptomyces lydicamycinicus	NPDC	identity >= 60 %	Streptomyces lydicamycinicus	Locus split across 2 contigs; contig and span refer to the contig carrying the largest part of the 24-gene locus, and span is measured over those genes, not over the three probe genes.
NPDC023103	1	1	1	NPDC023103.ctg-0001	25261	100.0/100	99.5/100	99.5/100	Streptomyces lydicamycinicus	NPDC	colocalisation	Streptomyces lydicamycinicus	
NPDC023112	1	0	1	NPDC023112.ctg-0165	13546	100.0/100	99.5/100	99.5/100	Streptomyces lydicamycinicus	NPDC	identity >= 60 %	Streptomyces lydicamycinicus	Species name taken from the NPDC download query; the strain is absent from the 2026-08-08 portal export (no oxytetracycline BGC call, 248 contigs); ANI 99.2 % to the type strain of S. lydicamycinicus. Locus split across 3 contigs; contig and span refer to the contig carrying the largest part of the 24-gene locus, and span is measured over those genes, not over the three probe genes.
NPDC024095	1	1	1	NPDC024095.ctg-0001	25285	99.6/100	99.5/100	98.9/100	Streptomyces lydicamycinicus	NPDC	colocalisation	Streptomyces lydicamycinicus	
NPDC024428	1	0	1	NPDC024428.ctg-0054	23503	100.0/100	99.5/100	99.5/100	Streptomyces lydicamycinicus	NPDC	identity >= 60 %	Streptomyces lydicamycinicus	Locus split across 2 contigs; contig and span refer to the contig carrying the largest part of the 24-gene locus, and span is measured over those genes, not over the three probe genes.
NPDC025141	1	1	1	NPDC025141.ctg-0002	25263	100.0/100	99.3/100	99.5/100	Streptomyces lydicamycinicus	NPDC	colocalisation	Streptomyces lydicamycinicus	
NPDC025511	1	1	1	NPDC025511.ctg-0005	25262	100.0/100	99.5/100	99.5/100	Streptomyces lydicamycinicus	NPDC	colocalisation	Streptomyces lydicamycinicus	
NPDC032166	1	0	1	NPDC032166.ctg-0056	23502	100.0/100	99.3/100	99.5/100	Streptomyces lydicamycinicus	NPDC	identity >= 60 %	Streptomyces lydicamycinicus	Locus split across 2 contigs; contig and span refer to the contig carrying the

													largest part of the 24-gene locus, and span is measured over those genes, not over the three probe genes.
NPDC032947	1	0	1	NPDC032947.ctg-0048	23504	100.0/100	99.5/100	99.5/100	Streptomyces lydicamycinicus	NPDC	identity >= 60 %	Streptomyces lydicamycinicus	Locus split across 2 contigs; contig and span refer to the contig carrying the largest part of the 24-gene locus, and span is measured over those genes, not over the three probe genes.
NPDC033460	1	0	1	NPDC033460.ctg-0015	23504	100.0/100	99.5/100	99.7/100	Streptomyces lydicamycinicus	NPDC	identity >= 60 %	Streptomyces lydicamycinicus	Locus split across 2 contigs; contig and span refer to the contig carrying the largest part of the 24-gene locus, and span is measured over those genes, not over the three probe genes.
NPDC033730	1	1	1	NPDC033730.ctg-0002	25256	100.0/100	99.3/100	99.5/100	Streptomyces lydicamycinicus	NPDC	colocalisation	Streptomyces lydicamycinicus	
NPDC025659	1	0	1	NPDC025659.ctg-0021	13674	83.3/100	92.5/100	88.0/100	Streptomyces platensis	NPDC	identity >= 60 %	Streptomyces glebosus / Streptomyces libani / Streptomyces platensis	Locus split across 3 contigs; contig and span refer to the contig carrying the largest part of the 24-gene locus, and span is measured over those genes, not over the three probe genes.

Table S6: The 30 collection assemblies of *S. lydicamycinicus* screened for the locus, with ANI to bja295, aligned fraction, number of locus genes recovered and the correspondence between collection and GenBank accessions.

Collection accession	GenBank accession	ANI to bja295, %	Aligned fraction, %	Locus genes recovered (of 22)	Span, bp	Median protein identity, %	Locus
NPDC032166	—	99.66	91.1	22	23379	99.7	+

NPDC008293	GCA_049538975.1	99.65	95.5	22	25614	99.7	+
NPDC020847	GCA_049540535.1	99.65	95.1	22	25617	99.6	+
NPDC025511	—	99.65	92.0	22	25614	99.7	+
NPDC088830	GCA_049543175.1	99.65	93.9	22	25614	99.7	+
NPDC006927	GCA_049538515.1	99.64	94.9	22	25616	99.4	+
NPDC023103	—	99.64	93.6	22	25614	99.7	+
NPDC025141	—	99.64	94.2	22	25615	99.7	+
NPDC030177	GCA_055665105.1	99.64	97.4	22	25615	99.6	+
NPDC030806	GCA_055664925.1	99.64	96.2	22	25614	99.7	+
NPDC001531	—	99.63	90.2	22	23372	99.7	+
NPDC001772	GCA_053596115.1	99.61	94.9	22	25618	99.7	+
NPDC024428	—	99.61	95.0	22	23380	99.7	+
NPDC023112	—	99.45	92.1	22	14592	99.4	+
NPDC035741	GCA_055668365.1	99.45	89.8	22	25600	99.6	+
NPDC032947	—	99.42	94.5	22	23381	99.5	+
NPDC033730	—	99.42	91.3	22	25609	99.5	+

NPDC039428	GCA_055668245.1	99.42	92.4	22	25616	99.6	+
NPDC029235	GCA_055664545.1	99.41	92.5	22	25774	99.6	+
NPDC033460	—	99.40	94.2	22	23381	99.5	+
NPDC024095	—	98.88	90.2	22	25638	98.9	+
NPDC029466	GCA_055574515.1	97.48	83.3	22	25474	95.8	+
NPDC033176	GCA_055577735.1	96.82	81.5	0	—	45.1	–
NPDC002484	GCA_042889705.1	96.52	78.2	0	—	46.6	–
NPDC020741	GCA_044334665.1	96.48	79.8	0	—	46.6	–
NPDC020760	GCA_044334605.1	96.48	79.9	0	—	46.6	–
NPDC101287	—	96.35	76.1	0	—	46.1	–
NPDC127097	GCA_042724075.1	96.28	78.1	0	—	46.8	–
NPDC033267	GCA_055576615.1	96.27	75.1	0	—	46.1	–
NPDC057006	—	96.24	76.4	0	—	47.3	–

Table S7: The 1,035 pairwise ANI comparisons over the 46-genome panel of the S. lydicamycinicus complex, from which the genomospecies threshold was derived. ANI was computed with skani v0.3.2 (-s 70 --min-af 5 --median) and, independently, with FastANI v1.34; AF is the aligned fraction reported by skani for each member of the pair. Genomospecies assignments and dDDH values are those of Table S3. Values within genomospecies (97.77-100.00 %) and between them (95.68-97.52 %) do not overlap, so that any threshold between 97.5 % and 97.8 % separates them without error. The full 46 x 46 matrix is provided as a separate data file.

Genome 1	Genome 2	ANI, % (skani --median)	AF 1, %	AF 2, %	ANI, % (FastANI)	Genomospecies 1	Genomospecies 2	Same genomospecies	dDDH d4, %
CW25	MMS22-DDSA8	99.64	96.00	95.70	99.42	I	I	yes	94.8
CW25	NPDC001531	99.63	95.07	90.24	99.38	I	I	yes	95.0
CW25	NPDC001772	99.61	95.51	94.90	99.40	I	I	yes	94.7
CW25	NPDC002484	96.52	82.48	78.16	95.67	I	IV	no	62.7
CW25	NPDC006927	99.64	95.51	94.89	99.44	I	I	yes	94.6
CW25	NPDC008293	99.65	95.94	95.45	99.48	I	I	yes	95.1
CW25	NPDC020741	96.48	82.12	79.84	95.68	I	IV	no	62.7
CW25	NPDC020760	96.48	82.14	79.86	95.68	I	IV	no	62.7
CW25	NPDC020847	99.65	95.80	95.11	99.45	I	I	yes	95.1
CW25	NPDC023103	99.64	97.25	93.56	99.48	I	I	yes	95.5
CW25	NPDC023112	99.45	93.73	92.12	99.09	I	I	yes	92.3
CW25	NPDC024095	98.88	91.95	90.19	98.41	I	I	yes	85.4
CW25	NPDC024428	99.61	95.41	94.98	99.39	I	I	yes	94.5
CW25	NPDC025141	99.64	96.28	94.18	99.44	I	I	yes	94.7
CW25	NPDC025256	96.31	79.44	74.40	95.25	I	II	no	59.8
CW25	NPDC025511	99.65	97.15	92.05	99.48	I	I	yes	95.5
CW25	NPDC025659	96.32	77.42	76.21	95.15	I	II	no	59.7
CW25	NPDC029235	99.41	93.92	92.53	99.06	I	I	yes	92.1
CW25	NPDC029466	97.48	86.56	83.32	96.62	I	VII	no	68.8
CW25	NPDC029498	96.13	78.75	74.94	95.08	I	VI	no	58.1
CW25	NPDC030177	99.64	95.32	97.37	99.47	I	I	yes	95.2
CW25	NPDC030806	99.64	96.01	96.23	99.47	I	I	yes	95.4
CW25	NPDC032166	99.66	95.89	91.08	99.45	I	I	yes	95.0
CW25	NPDC032794	96.22	79.19	74.35	95.28	I	II	no	59.9
CW25	NPDC032847	96.30	79.57	78.36	95.33	I	II	no	60.0
CW25	NPDC032947	99.42	93.53	94.50	99.14	I	I	yes	92.0
CW25	NPDC033176	96.82	80.93	81.50	95.89	I	VIII	no	64.5
CW25	NPDC033267	96.27	81.84	75.11	95.34	I	III	no	58.8
CW25	NPDC033460	99.40	94.14	94.23	99.07	I	I	yes	91.7
CW25	NPDC033730	99.42	93.72	91.26	99.11	I	I	yes	92.0
CW25	NPDC034584	96.23	79.50	75.54	95.27	I	II	no	59.8
CW25	NPDC035741	99.45	94.20	89.76	99.10	I	I	yes	91.8
CW25	NPDC039428	99.42	93.86	92.44	99.09	I	I	yes	92.0
CW25	NPDC041037	96.11	79.14	75.02	95.02	I	V	no	58.1
CW25	NPDC042329	96.28	79.17	77.90	95.31	I	II	no	59.8
CW25	NPDC044766	96.27	78.94	77.74	95.27	I	II	no	60.1
CW25	NPDC046625	96.34	78.44	75.55	95.23	I	II	no	60.0
CW25	NPDC046888	95.92	77.97	73.48	95.03	I	V	no	58.0
CW25	NPDC047015	95.94	77.77	75.99	95.07	I	V	no	58.0
CW25	NPDC052111	96.11	78.94	76.45	95.12	I	VI	no	58.0
CW25	NPDC057006	96.24	82.44	76.44	95.32	I	III	no	59.2
CW25	NPDC088830	99.65	96.31	93.92	99.43	I	I	yes	94.8

CW25	NPDC101287	96.35	81.83	76.11	95.26	I	III	no	59.2
CW25	NPDC127097	96.28	81.32	78.11	95.32	I	III	no	59.4
CW25	TP-A0598	99.68	95.59	94.19	99.44	I	I	yes	95.5
MMS22-DDSA8	NPDC001531	99.63	97.35	92.70	99.44	I	I	yes	95.4
MMS22-DDSA8	NPDC001772	99.62	97.05	96.75	99.45	I	I	yes	95.2
MMS22-DDSA8	NPDC002484	96.49	81.86	77.82	95.68	I	IV	no	62.5
MMS22-DDSA8	NPDC006927	99.65	96.83	96.49	99.48	I	I	yes	95.0
MMS22-DDSA8	NPDC008293	99.64	95.56	95.37	99.42	I	I	yes	95.1
MMS22-DDSA8	NPDC020741	96.44	81.79	79.77	95.67	I	IV	no	62.5
MMS22-DDSA8	NPDC020760	96.44	81.75	79.73	95.69	I	IV	no	62.5
MMS22-DDSA8	NPDC020847	99.64	96.76	96.37	99.48	I	I	yes	95.4
MMS22-DDSA8	NPDC023103	99.63	96.14	92.79	99.43	I	I	yes	95.0
MMS22-DDSA8	NPDC023112	99.43	93.16	91.85	99.09	I	I	yes	92.2
MMS22-DDSA8	NPDC024095	98.84	91.62	90.16	98.33	I	I	yes	85.2
MMS22-DDSA8	NPDC024428	99.65	95.44	95.31	99.42	I	I	yes	95.3
MMS22-DDSA8	NPDC025141	99.62	96.86	95.04	99.43	I	I	yes	94.6
MMS22-DDSA8	NPDC025256	96.26	79.03	74.26	95.18	I	II	no	
MMS22-DDSA8	NPDC025511	99.63	95.92	91.17	99.45	I	I	yes	95.3
MMS22-DDSA8	NPDC025659	96.26	77.38	76.41	95.09	I	II	no	
MMS22-DDSA8	NPDC029235	99.40	93.46	92.36	99.05	I	I	yes	91.9
MMS22-DDSA8	NPDC029466	97.45	86.09	83.13	96.62	I	VII	no	68.7
MMS22-DDSA8	NPDC029498	96.15	78.00	74.46	95.08	I	VI	no	
MMS22-DDSA8	NPDC030177	99.62	95.12	97.47	99.46	I	I	yes	95.3
MMS22-DDSA8	NPDC030806	99.64	96.02	96.55	99.47	I	I	yes	95.5
MMS22-DDSA8	NPDC032166	99.62	95.70	91.19	99.36	I	I	yes	94.6
MMS22-DDSA8	NPDC032794	96.30	78.72	74.14	95.22	I	II	no	
MMS22-DDSA8	NPDC032847	96.23	78.88	77.93	95.28	I	II	no	
MMS22-DDSA8	NPDC032947	99.42	92.99	94.26	99.09	I	I	yes	91.9
MMS22-DDSA8	NPDC033176	96.84	80.62	81.45	95.86	I	VIII	no	64.5
MMS22-DDSA8	NPDC033267	96.25	81.37	74.92	95.30	I	III	no	59.1
MMS22-DDSA8	NPDC033460	99.41	93.28	93.67	99.08	I	I	yes	92.0
MMS22-DDSA8	NPDC033730	99.42	93.29	91.12	99.07	I	I	yes	92.0
MMS22-DDSA8	NPDC034584	96.19	79.02	75.33	95.23	I	II	no	
MMS22-DDSA8	NPDC035741	99.42	93.88	89.73	99.01	I	I	yes	91.3
MMS22-DDSA8	NPDC039428	99.42	93.63	92.50	99.05	I	I	yes	91.8
MMS22-DDSA8	NPDC041037	96.04	78.10	74.26	95.06	I	V	no	
MMS22-DDSA8	NPDC042329	96.26	78.46	77.45	95.23	I	II	no	
MMS22-DDSA8	NPDC044766	96.26	78.31	77.36	95.24	I	II	no	
MMS22-DDSA8	NPDC046625	96.30	78.11	75.47	95.20	I	II	no	
MMS22-DDSA8	NPDC046888	95.90	77.59	73.36	94.97	I	V	no	
MMS22-DDSA8	NPDC047015	95.95	77.51	75.96	94.94	I	V	no	
MMS22-DDSA8	NPDC052111	96.09	78.28	76.05	95.08	I	VI	no	
MMS22-DDSA8	NPDC057006	96.23	82.32	76.56	95.31	I	III	no	59.0

MMS22-DDSA8	NPDC088830	99.64	96.10	94.01	99.44	I	I	yes	94.9
MMS22-DDSA8	NPDC101287	96.35	81.18	75.75	95.24	I	III	no	59.1
MMS22-DDSA8	NPDC127097	96.24	80.80	77.86	95.32	I	III	no	59.2
MMS22-DDSA8	TP-A0598	99.61	96.54	95.43	99.43	I	I	yes	95.1
NPDC001531	NPDC001772	99.61	91.93	96.24	99.41	I	I	yes	95.3
NPDC001531	NPDC002484	96.48	77.53	77.40	95.67	I	IV	no	62.7
NPDC001531	NPDC006927	99.63	92.13	96.42	99.36	I	I	yes	95.1
NPDC001531	NPDC008293	99.65	90.76	95.14	99.44	I	I	yes	95.4
NPDC001531	NPDC020741	96.43	77.45	79.33	95.64	I	IV	no	62.6
NPDC001531	NPDC020760	96.43	77.43	79.31	95.65	I	IV	no	62.6
NPDC001531	NPDC020847	99.65	92.00	96.23	99.42	I	I	yes	95.5
NPDC001531	NPDC023103	99.64	91.30	92.54	99.40	I	I	yes	95.2
NPDC001531	NPDC023112	99.47	88.59	91.74	99.11	I	I	yes	92.6
NPDC001531	NPDC024095	98.83	86.87	89.77	98.34	I	I	yes	85.5
NPDC001531	NPDC024428	99.64	90.52	94.93	99.38	I	I	yes	95.3
NPDC001531	NPDC025141	99.65	91.78	94.58	99.40	I	I	yes	95.1
NPDC001531	NPDC025256	96.34	74.91	73.92	95.12	I	II	no	59.5
NPDC001531	NPDC025511	99.64	91.46	91.30	99.45	I	I	yes	95.3
NPDC001531	NPDC025659	96.31	73.40	76.13	95.13	I	II	no	59.7
NPDC001531	NPDC029235	99.44	88.83	92.19	99.09	I	I	yes	92.3
NPDC001531	NPDC029466	97.49	81.50	82.65	96.64	I	VII	no	69.0
NPDC001531	NPDC029498	96.12	73.67	73.86	95.04	I	VI	no	58.2
NPDC001531	NPDC030177	99.63	90.24	97.11	99.44	I	I	yes	95.3
NPDC001531	NPDC030806	99.65	91.19	96.30	99.46	I	I	yes	95.6
NPDC001531	NPDC032166	99.65	90.75	90.82	99.34	I	I	yes	95.0
NPDC001531	NPDC032794	96.36	74.68	73.87	95.27	I	II	no	60.1
NPDC001531	NPDC032847	96.31	74.56	77.36	95.27	I	II	no	60.1
NPDC001531	NPDC032947	99.43	88.65	94.37	99.10	I	I	yes	92.1
NPDC001531	NPDC033176	96.79	76.33	80.99	95.92	I	VIII	no	64.8
NPDC001531	NPDC033267	96.30	76.34	73.81	95.28	I	III	no	59.2
NPDC001531	NPDC033460	99.42	88.90	93.76	99.10	I	I	yes	92.3
NPDC001531	NPDC033730	99.43	88.98	91.28	99.05	I	I	yes	91.9
NPDC001531	NPDC034584	96.36	74.68	74.77	95.19	I	II	no	60.0
NPDC001531	NPDC035741	99.43	88.89	89.23	99.03	I	I	yes	91.9
NPDC001531	NPDC039428	99.43	88.96	92.30	99.00	I	I	yes	91.7
NPDC001531	NPDC041037	96.21	74.43	74.33	95.03	I	V	no	58.4
NPDC001531	NPDC042329	96.33	74.60	77.34	95.22	I	II	no	59.8
NPDC001531	NPDC044766	96.24	74.28	77.07	95.24	I	II	no	60.0
NPDC001531	NPDC046625	96.34	73.76	74.85	95.17	I	II	no	60.2
NPDC001531	NPDC046888	96.11	73.48	72.96	95.00	I	V	no	58.1
NPDC001531	NPDC047015	96.06	73.65	75.81	94.98	I	V	no	58.1
NPDC001531	NPDC052111	96.13	73.88	75.39	95.05	I	VI	no	58.2
NPDC001531	NPDC057006	96.43	77.71	75.90	95.20	I	III	no	59.1

NPDC001531	NPDC088830	99.65	91.31	93.81	99.42	I	I	yes	95.2
NPDC001531	NPDC101287	96.37	76.83	75.28	95.11	I	III	no	58.9
NPDC001531	NPDC127097	96.33	76.60	77.52	95.30	I	III	no	59.6
NPDC001531	TP-A0598	99.64	91.87	95.37	99.43	I	I	yes	95.5
NPDC001772	NPDC002484	96.48	81.14	77.39	95.68	I	IV	no	62.7
NPDC001772	NPDC006927	99.65	96.99	96.96	99.49	I	I	yes	95.2
NPDC001772	NPDC008293	99.64	95.10	95.22	99.35	I	I	yes	94.5
NPDC001772	NPDC020741	96.49	81.41	79.65	95.70	I	IV	no	62.6
NPDC001772	NPDC020760	96.49	81.43	79.67	95.70	I	IV	no	62.6
NPDC001772	NPDC020847	99.64	96.41	96.33	99.44	I	I	yes	95.1
NPDC001772	NPDC023103	99.64	95.48	92.45	99.39	I	I	yes	94.9
NPDC001772	NPDC023112	99.45	92.85	91.84	99.11	I	I	yes	92.4
NPDC001772	NPDC024095	98.86	91.32	90.14	98.40	I	I	yes	85.6
NPDC001772	NPDC024428	99.65	95.45	95.62	99.41	I	I	yes	95.1
NPDC001772	NPDC025141	99.64	96.70	95.19	99.46	I	I	yes	94.9
NPDC001772	NPDC025256	96.27	78.81	74.29	95.26	I	II	no	
NPDC001772	NPDC025511	99.62	95.46	91.02	99.39	I	I	yes	95.0
NPDC001772	NPDC025659	96.31	77.31	76.59	95.17	I	II	no	
NPDC001772	NPDC029235	99.41	92.99	92.19	99.10	I	I	yes	92.3
NPDC001772	NPDC029466	97.48	85.76	83.08	96.62	I	VII	no	68.7
NPDC001772	NPDC029498	96.15	78.18	74.88	95.09	I	VI	no	
NPDC001772	NPDC030177	99.64	94.99	97.65	99.45	I	I	yes	95.3
NPDC001772	NPDC030806	99.64	95.29	96.12	99.45	I	I	yes	95.3
NPDC001772	NPDC032166	99.63	95.64	91.42	99.41	I	I	yes	94.7
NPDC001772	NPDC032794	96.27	78.25	73.94	95.30	I	II	no	
NPDC001772	NPDC032847	96.29	78.57	77.87	95.31	I	II	no	
NPDC001772	NPDC032947	99.43	92.82	94.38	99.12	I	I	yes	91.9
NPDC001772	NPDC033176	96.84	79.98	81.07	95.91	I	VIII	no	64.6
NPDC001772	NPDC033267	96.30	80.68	74.52	95.30	I	III	no	59.1
NPDC001772	NPDC033460	99.42	93.02	93.70	99.12	I	I	yes	92.3
NPDC001772	NPDC033730	99.43	92.96	91.09	99.10	I	I	yes	92.1
NPDC001772	NPDC034584	96.24	78.73	75.29	95.20	I	II	no	
NPDC001772	NPDC035741	99.43	93.27	89.44	99.07	I	I	yes	91.8
NPDC001772	NPDC039428	99.44	93.06	92.23	99.09	I	I	yes	92.1
NPDC001772	NPDC041037	96.14	77.91	74.32	95.08	I	V	no	
NPDC001772	NPDC042329	96.24	78.60	77.83	95.27	I	II	no	
NPDC001772	NPDC044766	96.29	77.93	77.23	95.24	I	II	no	
NPDC001772	NPDC046625	96.36	77.91	75.51	95.19	I	II	no	
NPDC001772	NPDC046888	96.05	77.51	73.52	94.98	I	V	no	
NPDC001772	NPDC047015	96.06	77.06	75.76	95.02	I	V	no	
NPDC001772	NPDC052111	96.12	78.17	76.19	95.15	I	VI	no	
NPDC001772	NPDC057006	96.34	81.21	75.77	95.35	I	III	no	59.3
NPDC001772	NPDC088830	99.63	95.47	93.69	99.42	I	I	yes	94.7

NPDC001772	NPDC101287	96.36	80.88	75.71	95.24	I	III	no	59.1
NPDC001772	NPDC127097	96.28	80.34	77.67	95.36	I	III	no	59.4
NPDC001772	TP-A0598	99.63	96.03	95.22	99.41	I	I	yes	95.2
NPDC002484	NPDC006927	96.52	77.61	81.36	95.66	IV	I	no	62.6
NPDC002484	NPDC008293	96.52	77.48	81.34	95.68	IV	I	no	62.8
NPDC002484	NPDC020741	99.76	94.07	96.51	99.58	IV	IV	yes	96.8
NPDC002484	NPDC020760	99.76	94.10	96.54	99.60	IV	IV	yes	96.8
NPDC002484	NPDC020847	96.46	77.63	81.33	95.72	IV	I	no	62.6
NPDC002484	NPDC023103	96.47	77.77	78.96	95.63	IV	I	no	62.7
NPDC002484	NPDC023112	96.45	76.95	79.80	95.46	IV	I	no	62.0
NPDC002484	NPDC024095	96.47	77.18	79.88	95.59	IV	I	no	62.1
NPDC002484	NPDC024428	96.48	77.17	81.06	95.69	IV	I	no	62.6
NPDC002484	NPDC025141	96.44	78.05	80.56	95.66	IV	I	no	62.5
NPDC002484	NPDC025256	96.29	76.10	75.21	95.36	IV	II	no	
NPDC002484	NPDC025511	96.53	77.27	77.25	95.70	IV	I	no	62.7
NPDC002484	NPDC025659	96.42	74.81	77.71	95.32	IV	II	no	
NPDC002484	NPDC029235	96.49	77.28	80.34	95.64	IV	I	no	62.3
NPDC002484	NPDC029466	96.27	76.37	77.57	95.32	IV	VII	no	59.4
NPDC002484	NPDC029498	96.17	74.63	74.94	95.00	IV	VI	no	
NPDC002484	NPDC030177	96.51	77.81	83.87	95.71	IV	I	no	62.8
NPDC002484	NPDC030806	96.49	77.52	81.99	95.69	IV	I	no	62.8
NPDC002484	NPDC032166	96.53	77.70	77.88	95.54	IV	I	no	62.3
NPDC002484	NPDC032794	96.36	76.23	75.52	95.30	IV	II	no	
NPDC002484	NPDC032847	96.40	76.38	79.37	95.41	IV	II	no	
NPDC002484	NPDC032947	96.39	77.24	82.36	95.68	IV	I	no	62.4
NPDC002484	NPDC033176	96.51	74.94	79.64	95.54	IV	VIII	no	62.7
NPDC002484	NPDC033267	96.06	77.24	74.80	95.09	IV	III	no	58.0
NPDC002484	NPDC033460	96.43	77.57	81.93	95.62	IV	I	no	62.2
NPDC002484	NPDC033730	96.48	77.10	79.21	95.61	IV	I	no	62.3
NPDC002484	NPDC034584	96.39	75.93	76.14	95.37	IV	II	no	
NPDC002484	NPDC035741	96.44	77.25	77.67	95.61	IV	I	no	62.0
NPDC002484	NPDC039428	96.50	77.55	80.58	95.63	IV	I	no	62.2
NPDC002484	NPDC041037	96.19	77.18	77.19	95.19	IV	V	no	
NPDC002484	NPDC042329	96.33	76.54	79.46	95.40	IV	II	no	
NPDC002484	NPDC044766	96.40	76.09	79.07	95.37	IV	II	no	
NPDC002484	NPDC046625	96.34	76.09	77.33	95.28	IV	II	no	
NPDC002484	NPDC046888	96.08	76.43	76.01	95.13	IV	V	no	
NPDC002484	NPDC047015	96.13	76.16	78.52	95.12	IV	V	no	
NPDC002484	NPDC052111	96.04	75.35	77.00	95.12	IV	VI	no	
NPDC002484	NPDC057006	96.00	77.66	75.98	95.11	IV	III	no	58.2
NPDC002484	NPDC088830	96.49	77.94	80.20	95.65	IV	I	no	62.4
NPDC002484	NPDC101287	96.07	76.69	75.27	95.04	IV	III	no	58.1
NPDC002484	NPDC127097	96.00	76.63	77.67	95.12	IV	III	no	58.2

NPDC002484	TP-A0598	96.50	77.94	81.04	95.71	IV	I	no	62.6
NPDC006927	NPDC008293	99.65	94.99	95.14	99.46	I	I	yes	95.3
NPDC006927	NPDC020741	96.53	81.40	79.67	95.69	I	IV	no	62.7
NPDC006927	NPDC020760	96.51	81.42	79.68	95.72	I	IV	no	62.7
NPDC006927	NPDC020847	99.67	95.97	95.91	99.48	I	I	yes	95.4
NPDC006927	NPDC023103	99.67	95.22	92.22	99.46	I	I	yes	95.5
NPDC006927	NPDC023112	99.44	92.89	91.90	99.14	I	I	yes	92.4
NPDC006927	NPDC024095	98.88	91.36	90.20	98.42	I	I	yes	85.7
NPDC006927	NPDC024428	99.65	95.44	95.64	99.44	I	I	yes	95.3
NPDC006927	NPDC025141	99.68	96.32	94.83	99.49	I	I	yes	95.3
NPDC006927	NPDC025256	96.26	78.90	74.39	95.17	I	II	no	
NPDC006927	NPDC025511	99.65	95.15	90.75	99.45	I	I	yes	95.1
NPDC006927	NPDC025659	96.29	77.30	76.60	95.15	I	II	no	
NPDC006927	NPDC029235	99.40	93.04	92.26	99.11	I	I	yes	92.4
NPDC006927	NPDC029466	97.52	85.49	82.83	96.64	I	VII	no	68.8
NPDC006927	NPDC029498	96.16	78.31	75.01	95.11	I	VI	no	
NPDC006927	NPDC030177	99.64	95.11	97.79	99.49	I	I	yes	95.6
NPDC006927	NPDC030806	99.66	95.26	96.11	99.48	I	I	yes	95.7
NPDC006927	NPDC032166	99.68	95.58	91.39	99.42	I	I	yes	95.3
NPDC006927	NPDC032794	96.28	78.53	74.22	95.34	I	II	no	
NPDC006927	NPDC032847	96.33	78.71	78.03	95.29	I	II	no	
NPDC006927	NPDC032947	99.41	92.80	94.39	99.13	I	I	yes	91.8
NPDC006927	NPDC033176	96.83	80.31	81.42	95.91	I	VIII	no	64.6
NPDC006927	NPDC033267	96.29	80.81	74.66	95.33	I	III	no	59.0
NPDC006927	NPDC033460	99.45	93.10	93.81	99.14	I	I	yes	92.4
NPDC006927	NPDC033730	99.42	92.79	90.95	99.09	I	I	yes	92.0
NPDC006927	NPDC034584	96.25	78.50	75.09	95.23	I	II	no	
NPDC006927	NPDC035741	99.41	93.65	89.83	99.06	I	I	yes	91.2
NPDC006927	NPDC039428	99.42	93.11	92.30	99.09	I	I	yes	92.0
NPDC006927	NPDC041037	96.13	77.99	74.41	95.10	I	V	no	
NPDC006927	NPDC042329	96.30	78.35	77.60	95.29	I	II	no	
NPDC006927	NPDC044766	96.32	78.11	77.43	95.29	I	II	no	
NPDC006927	NPDC046625	96.35	77.74	75.38	95.26	I	II	no	
NPDC006927	NPDC046888	95.92	77.42	73.45	94.99	I	V	no	
NPDC006927	NPDC047015	95.96	77.37	76.09	95.05	I	V	no	
NPDC006927	NPDC052111	96.03	78.25	76.28	95.14	I	VI	no	
NPDC006927	NPDC057006	96.29	81.98	76.51	95.31	I	III	no	59.1
NPDC006927	NPDC088830	99.65	95.14	93.39	99.45	I	I	yes	95.1
NPDC006927	NPDC101287	96.35	80.86	75.71	95.21	I	III	no	58.8
NPDC006927	NPDC127097	96.27	80.64	77.97	95.36	I	III	no	59.5
NPDC006927	TP-A0598	99.65	95.72	94.94	99.42	I	I	yes	95.0
NPDC008293	NPDC020741	96.52	81.71	79.85	95.73	I	IV	no	62.6
NPDC008293	NPDC020760	96.52	81.72	79.85	95.74	I	IV	no	62.6

NPDC008293	NPDC020847	99.67	95.68	95.48	99.51	I	I	yes	95.7
NPDC008293	NPDC023103	99.91	97.81	94.59	99.77	I	I	yes	98.3
NPDC008293	NPDC023112	99.46	93.05	91.92	99.10	I	I	yes	92.1
NPDC008293	NPDC024095	98.86	91.34	90.05	98.40	I	I	yes	86.0
NPDC008293	NPDC024428	99.66	95.24	95.30	99.45	I	I	yes	95.6
NPDC008293	NPDC025141	99.68	95.48	93.87	99.47	I	I	yes	95.4
NPDC008293	NPDC025256	96.37	79.44	74.78	95.33	I	II	no	
NPDC008293	NPDC025511	100.00	97.78	93.11	99.93	I	I	yes	99.5
NPDC008293	NPDC025659	96.32	77.21	76.39	95.15	I	II	no	
NPDC008293	NPDC029235	99.42	93.29	92.37	99.08	I	I	yes	92.2
NPDC008293	NPDC029466	97.49	85.71	82.92	96.62	I	VII	no	69.0
NPDC008293	NPDC029498	96.15	78.17	74.77	95.13	I	VI	no	
NPDC008293	NPDC030177	99.65	94.77	97.30	99.48	I	I	yes	95.7
NPDC008293	NPDC030806	99.98	96.94	97.65	99.86	I	I	yes	99.0
NPDC008293	NPDC032166	99.68	95.28	90.96	99.44	I	I	yes	95.4
NPDC008293	NPDC032794	96.30	78.39	73.97	95.32	I	II	no	
NPDC008293	NPDC032847	96.28	78.67	77.87	95.33	I	II	no	
NPDC008293	NPDC032947	99.44	92.96	94.41	99.13	I	I	yes	92.0
NPDC008293	NPDC033176	96.84	80.23	81.21	95.94	I	VIII	no	64.7
NPDC008293	NPDC033267	96.27	80.76	74.49	95.37	I	III	no	59.3
NPDC008293	NPDC033460	99.42	93.37	93.94	99.14	I	I	yes	92.4
NPDC008293	NPDC033730	99.44	93.19	91.20	99.10	I	I	yes	92.0
NPDC008293	NPDC034584	96.26	79.08	75.53	95.25	I	II	no	
NPDC008293	NPDC035741	99.44	93.59	89.63	99.10	I	I	yes	91.8
NPDC008293	NPDC039428	99.44	93.33	92.38	99.09	I	I	yes	92.0
NPDC008293	NPDC041037	96.09	78.30	74.60	95.08	I	V	no	
NPDC008293	NPDC042329	96.31	78.46	77.59	95.31	I	II	no	
NPDC008293	NPDC044766	96.33	78.17	77.37	95.27	I	II	no	
NPDC008293	NPDC046625	96.32	77.39	74.92	95.26	I	II	no	
NPDC008293	NPDC046888	95.96	77.90	73.79	95.03	I	V	no	
NPDC008293	NPDC047015	95.96	77.85	76.45	95.09	I	V	no	
NPDC008293	NPDC052111	96.10	78.20	76.12	95.09	I	VI	no	
NPDC008293	NPDC057006	96.27	81.81	76.23	95.40	I	III	no	59.4
NPDC008293	NPDC088830	100.00	97.87	95.92	99.97	I	I	yes	99.8
NPDC008293	NPDC101287	96.39	80.81	75.54	95.27	I	III	no	59.3
NPDC008293	NPDC127097	96.28	81.10	78.30	95.35	I	III	no	59.3
NPDC008293	TP-A0598	99.65	94.88	93.96	99.44	I	I	yes	95.2
NPDC020741	NPDC020760	100.00	100.00	100.00	100.00	IV	IV	yes	100.0
NPDC020741	NPDC020847	96.53	79.67	81.35	95.69	IV	I	no	62.6
NPDC020741	NPDC023103	96.47	80.02	79.18	95.67	IV	I	no	62.5
NPDC020741	NPDC023112	96.50	79.23	80.09	95.59	IV	I	no	62.2
NPDC020741	NPDC024095	96.46	79.24	79.94	95.54	IV	I	no	62.1
NPDC020741	NPDC024428	96.57	79.13	81.02	95.67	IV	I	no	62.5

NPDC020741	NPDC025141	96.50	79.54	80.02	95.74	IV	I	no	62.6
NPDC020741	NPDC025256	96.31	77.95	75.09	95.36	IV	II	no	
NPDC020741	NPDC025511	96.55	79.49	77.47	95.69	IV	I	no	62.7
NPDC020741	NPDC025659	96.43	76.85	77.81	95.32	IV	II	no	
NPDC020741	NPDC029235	96.51	79.54	80.59	95.64	IV	I	no	62.2
NPDC020741	NPDC029466	96.28	77.85	77.07	95.34	IV	VII	no	59.6
NPDC020741	NPDC029498	96.15	76.39	74.77	95.14	IV	VI	no	
NPDC020741	NPDC030177	96.52	79.53	83.55	95.78	IV	I	no	62.8
NPDC020741	NPDC030806	96.54	79.63	82.09	95.77	IV	I	no	62.7
NPDC020741	NPDC032166	96.58	79.58	77.75	95.70	IV	I	no	62.7
NPDC020741	NPDC032794	96.34	78.31	75.63	95.37	IV	II	no	
NPDC020741	NPDC032847	96.38	78.25	79.26	95.40	IV	II	no	
NPDC020741	NPDC032947	96.41	79.34	82.46	95.71	IV	I	no	62.5
NPDC020741	NPDC033176	96.57	76.80	79.55	95.64	IV	VIII	no	63.1
NPDC020741	NPDC033267	96.05	78.75	74.34	95.15	IV	III	no	58.1
NPDC020741	NPDC033460	96.46	79.29	81.64	95.66	IV	I	no	62.2
NPDC020741	NPDC033730	96.48	79.42	79.53	95.69	IV	I	no	62.5
NPDC020741	NPDC034584	96.39	77.96	76.20	95.43	IV	II	no	
NPDC020741	NPDC035741	96.45	79.23	77.65	95.69	IV	I	no	62.3
NPDC020741	NPDC039428	96.41	79.74	80.76	95.70	IV	I	no	62.4
NPDC020741	NPDC041037	96.17	78.93	76.95	95.26	IV	V	no	
NPDC020741	NPDC042329	96.32	78.57	79.52	95.43	IV	II	no	
NPDC020741	NPDC044766	96.41	77.79	78.79	95.41	IV	II	no	
NPDC020741	NPDC046625	96.34	77.72	77.00	95.27	IV	II	no	
NPDC020741	NPDC046888	96.09	77.71	75.33	95.16	IV	V	no	
NPDC020741	NPDC047015	96.12	77.71	78.09	95.13	IV	V	no	
NPDC020741	NPDC052111	96.05	76.92	76.62	95.16	IV	VI	no	
NPDC020741	NPDC057006	96.04	79.51	75.82	95.12	IV	III	no	58.3
NPDC020741	NPDC088830	96.50	79.81	80.05	95.68	IV	I	no	62.6
NPDC020741	NPDC101287	96.11	78.21	74.82	95.10	IV	III	no	58.3
NPDC020741	NPDC127097	96.05	78.67	77.72	95.11	IV	III	no	58.1
NPDC020741	TP-A0598	96.47	80.13	81.20	95.73	IV	I	no	62.7
NPDC020760	NPDC020847	96.53	79.64	81.33	95.71	IV	I	no	62.6
NPDC020760	NPDC023103	96.47	80.04	79.21	95.66	IV	I	no	62.5
NPDC020760	NPDC023112	96.50	79.23	80.10	95.60	IV	I	no	62.2
NPDC020760	NPDC024095	96.46	79.25	79.95	95.57	IV	I	no	62.1
NPDC020760	NPDC024428	96.57	79.16	81.06	95.69	IV	I	no	62.5
NPDC020760	NPDC025141	96.50	79.58	80.07	95.73	IV	I	no	62.6
NPDC020760	NPDC025256	96.31	77.94	75.09	95.36	IV	II	no	
NPDC020760	NPDC025511	96.55	79.49	77.47	95.69	IV	I	no	62.7
NPDC020760	NPDC025659	96.43	76.84	77.81	95.32	IV	II	no	
NPDC020760	NPDC029235	96.50	79.55	80.61	95.67	IV	I	no	62.2
NPDC020760	NPDC029466	96.28	77.90	77.13	95.31	IV	VII	no	59.6

NPDC020760	NPDC029498	96.15	76.39	74.78	95.14	IV	VI	no	
NPDC020760	NPDC030177	96.52	79.50	83.53	95.79	IV	I	no	62.8
NPDC020760	NPDC030806	96.54	79.61	82.07	95.72	IV	I	no	62.7
NPDC020760	NPDC032166	96.58	79.60	77.77	95.70	IV	I	no	62.7
NPDC020760	NPDC032794	96.34	78.29	75.61	95.36	IV	II	no	
NPDC020760	NPDC032847	96.38	78.26	79.27	95.42	IV	II	no	
NPDC020760	NPDC032947	96.41	79.35	82.47	95.70	IV	I	no	62.5
NPDC020760	NPDC033176	96.58	76.81	79.57	95.62	IV	VIII	no	63.1
NPDC020760	NPDC033267	96.05	78.73	74.32	95.15	IV	III	no	58.1
NPDC020760	NPDC033460	96.46	79.35	81.70	95.66	IV	I	no	62.2
NPDC020760	NPDC033730	96.48	79.45	79.57	95.69	IV	I	no	62.5
NPDC020760	NPDC034584	96.39	77.95	76.19	95.41	IV	II	no	
NPDC020760	NPDC035741	96.45	79.22	77.65	95.67	IV	I	no	62.3
NPDC020760	NPDC039428	96.41	79.76	80.79	95.67	IV	I	no	62.4
NPDC020760	NPDC041037	96.17	78.94	76.96	95.22	IV	V	no	
NPDC020760	NPDC042329	96.32	78.56	79.51	95.43	IV	II	no	
NPDC020760	NPDC044766	96.41	77.78	78.79	95.41	IV	II	no	
NPDC020760	NPDC046625	96.34	77.72	77.00	95.27	IV	II	no	
NPDC020760	NPDC046888	96.09	77.68	75.31	95.16	IV	V	no	
NPDC020760	NPDC047015	96.12	77.68	78.07	95.16	IV	V	no	
NPDC020760	NPDC052111	96.05	76.92	76.62	95.16	IV	VI	no	
NPDC020760	NPDC057006	96.04	79.52	75.83	95.11	IV	III	no	58.3
NPDC020760	NPDC088830	96.50	79.78	80.02	95.70	IV	I	no	62.7
NPDC020760	NPDC101287	96.11	78.20	74.81	95.09	IV	III	no	58.3
NPDC020760	NPDC127097	96.05	78.66	77.72	95.14	IV	III	no	58.1
NPDC020760	TP-A0598	96.46	80.12	81.20	95.74	IV	I	no	62.7
NPDC020847	NPDC023103	99.66	95.97	93.00	99.49	I	I	yes	95.5
NPDC020847	NPDC023112	99.45	92.80	91.87	99.13	I	I	yes	92.5
NPDC020847	NPDC024095	98.88	91.19	90.10	98.40	I	I	yes	85.7
NPDC020847	NPDC024428	99.64	95.76	96.02	99.46	I	I	yes	95.5
NPDC020847	NPDC025141	99.66	96.71	95.28	99.45	I	I	yes	95.0
NPDC020847	NPDC025256	96.33	78.57	74.13	95.22	I	II	no	
NPDC020847	NPDC025511	99.67	96.04	91.65	99.44	I	I	yes	95.2
NPDC020847	NPDC025659	96.33	77.19	76.53	95.14	I	II	no	
NPDC020847	NPDC029235	99.43	93.25	92.53	99.05	I	I	yes	92.1
NPDC020847	NPDC029466	97.49	85.86	83.24	96.71	I	VII	no	68.8
NPDC020847	NPDC029498	96.19	78.03	74.80	95.12	I	VI	no	
NPDC020847	NPDC030177	99.65	95.33	98.08	99.50	I	I	yes	95.5
NPDC020847	NPDC030806	99.67	95.79	96.71	99.51	I	I	yes	95.9
NPDC020847	NPDC032166	99.66	95.87	91.72	99.40	I	I	yes	94.8
NPDC020847	NPDC032794	96.28	78.51	74.24	95.28	I	II	no	
NPDC020847	NPDC032847	96.28	78.53	77.89	95.28	I	II	no	
NPDC020847	NPDC032947	99.44	92.53	94.18	99.12	I	I	yes	92.5

NPDC020847	NPDC033176	96.83	80.23	81.38	95.95	I	VIII	no	64.7
NPDC020847	NPDC033267	96.27	80.81	74.71	95.33	I	III	no	59.1
NPDC020847	NPDC033460	99.45	93.28	94.05	99.12	I	I	yes	92.3
NPDC020847	NPDC033730	99.44	93.23	91.43	99.06	I	I	yes	92.0
NPDC020847	NPDC034584	96.26	78.85	75.47	95.25	I	II	no	
NPDC020847	NPDC035741	99.46	93.45	89.69	99.10	I	I	yes	92.2
NPDC020847	NPDC039428	99.46	93.23	92.47	99.06	I	I	yes	92.1
NPDC020847	NPDC041037	96.08	78.23	74.69	95.03	I	V	no	
NPDC020847	NPDC042329	96.35	78.52	77.82	95.29	I	II	no	
NPDC020847	NPDC044766	96.29	78.18	77.54	95.24	I	II	no	
NPDC020847	NPDC046625	96.37	77.80	75.48	95.20	I	II	no	
NPDC020847	NPDC046888	95.91	77.55	73.62	94.98	I	V	no	
NPDC020847	NPDC047015	95.95	77.55	76.32	95.01	I	V	no	
NPDC020847	NPDC052111	96.14	78.14	76.22	95.15	I	VI	no	
NPDC020847	NPDC057006	96.29	82.45	76.99	95.35	I	III	no	59.2
NPDC020847	NPDC088830	99.67	95.93	94.22	99.48	I	I	yes	95.4
NPDC020847	NPDC101287	96.41	80.79	75.69	95.21	I	III	no	59.0
NPDC020847	NPDC127097	96.28	80.70	78.08	95.37	I	III	no	59.3
NPDC020847	TP-A0598	99.66	95.75	95.03	99.44	I	I	yes	95.6
NPDC023103	NPDC023112	99.48	90.32	92.27	98.97	I	I	yes	91.8
NPDC023103	NPDC024095	98.85	88.55	90.27	98.31	I	I	yes	85.3
NPDC023103	NPDC024428	99.65	92.28	95.49	99.41	I	I	yes	95.3
NPDC023103	NPDC025141	99.82	92.98	94.53	99.58	I	I	yes	96.8
NPDC023103	NPDC025256	96.36	77.39	75.34	95.12	I	II	no	59.3
NPDC023103	NPDC025511	99.91	96.51	95.05	99.77	I	I	yes	98.4
NPDC023103	NPDC025659	96.31	74.78	76.52	95.13	I	II	no	59.6
NPDC023103	NPDC029235	99.41	90.35	92.51	99.01	I	I	yes	91.8
NPDC023103	NPDC029466	97.49	83.07	83.11	96.59	I	VII	no	68.9
NPDC023103	NPDC029498	96.14	75.71	74.88	95.05	I	VI	no	58.2
NPDC023103	NPDC030177	99.65	91.89	97.56	99.45	I	I	yes	95.4
NPDC023103	NPDC030806	99.87	94.00	97.93	99.76	I	I	yes	98.0
NPDC023103	NPDC032166	99.82	92.77	91.58	99.52	I	I	yes	96.2
NPDC023103	NPDC032794	96.27	76.12	74.29	95.17	I	II	no	59.7
NPDC023103	NPDC032847	96.27	76.30	78.10	95.26	I	II	no	59.9
NPDC023103	NPDC032947	99.44	89.94	94.46	99.10	I	I	yes	92.1
NPDC023103	NPDC033176	96.83	78.01	81.65	95.93	I	VIII	no	64.5
NPDC023103	NPDC033267	96.30	78.21	74.61	95.31	I	III	no	59.2
NPDC023103	NPDC033460	99.43	90.48	94.14	99.05	I	I	yes	91.9
NPDC023103	NPDC033730	99.43	90.16	91.25	99.06	I	I	yes	92.0
NPDC023103	NPDC034584	96.24	76.43	75.49	95.12	I	II	no	59.6
NPDC023103	NPDC035741	99.45	90.66	89.79	99.05	I	I	yes	91.5
NPDC023103	NPDC039428	99.44	90.38	92.51	99.01	I	I	yes	91.6
NPDC023103	NPDC041037	96.11	75.96	74.83	94.92	I	V	no	58.2

NPDC023103	NPDC042329	96.31	75.99	77.72	95.14	I	II	no	59.6
NPDC023103	NPDC044766	96.30	75.81	77.60	95.13	I	II	no	59.8
NPDC023103	NPDC046625	96.32	75.40	75.48	95.09	I	II	no	59.8
NPDC023103	NPDC046888	95.93	75.90	74.35	94.92	I	V	no	57.5
NPDC023103	NPDC047015	95.95	75.40	76.57	95.02	I	V	no	58.0
NPDC023103	NPDC052111	96.08	75.70	76.20	95.07	I	VI	no	57.8
NPDC023103	NPDC057006	96.26	79.91	77.00	95.30	I	III	no	58.9
NPDC023103	NPDC088830	99.91	95.08	96.37	99.79	I	I	yes	98.3
NPDC023103	NPDC101287	96.38	78.52	75.90	95.20	I	III	no	59.1
NPDC023103	NPDC127097	96.25	78.84	78.71	95.29	I	III	no	59.2
NPDC023103	TP-A0598	99.65	92.17	94.39	99.42	I	I	yes	95.2
NPDC023112	NPDC024095	98.89	90.21	90.02	98.34	I	I	yes	86.1
NPDC023112	NPDC024428	99.45	91.90	93.08	99.14	I	I	yes	92.4
NPDC023112	NPDC025141	99.49	91.75	91.31	99.19	I	I	yes	93.0
NPDC023112	NPDC025256	96.36	77.30	73.67	95.23	I	II	no	60.0
NPDC023112	NPDC025511	99.46	91.88	88.57	99.08	I	I	yes	92.2
NPDC023112	NPDC025659	96.38	75.68	75.80	95.17	I	II	no	59.8
NPDC023112	NPDC029235	99.70	95.81	96.03	99.50	I	I	yes	96.0
NPDC023112	NPDC029466	97.47	84.17	82.43	96.59	I	VII	no	68.7
NPDC023112	NPDC029498	96.08	77.41	74.95	94.98	I	VI	no	57.7
NPDC023112	NPDC030177	99.45	91.83	95.44	99.14	I	I	yes	92.7
NPDC023112	NPDC030806	99.47	91.77	93.58	99.16	I	I	yes	92.9
NPDC023112	NPDC032166	99.50	92.24	89.14	99.08	I	I	yes	92.3
NPDC023112	NPDC032794	96.33	77.15	73.69	95.25	I	II	no	60.1
NPDC023112	NPDC032847	96.32	77.52	77.67	95.32	I	II	no	60.3
NPDC023112	NPDC032947	99.66	94.57	97.22	99.46	I	I	yes	95.4
NPDC023112	NPDC033176	96.85	79.59	81.56	95.80	I	VIII	no	64.2
NPDC023112	NPDC033267	96.31	78.81	73.59	95.24	I	III	no	59.1
NPDC023112	NPDC033460	99.66	94.12	95.86	99.42	I	I	yes	95.2
NPDC023112	NPDC033730	99.65	94.33	93.45	99.43	I	I	yes	95.3
NPDC023112	NPDC034584	96.31	77.71	75.13	95.16	I	II	no	59.9
NPDC023112	NPDC035741	99.65	94.49	91.60	99.39	I	I	yes	94.8
NPDC023112	NPDC039428	99.66	94.01	94.19	99.41	I	I	yes	95.0
NPDC023112	NPDC041037	96.13	77.28	74.53	95.08	I	V	no	58.5
NPDC023112	NPDC042329	96.37	77.51	77.59	95.27	I	II	no	60.1
NPDC023112	NPDC044766	96.28	77.01	77.16	95.25	I	II	no	60.2
NPDC023112	NPDC046625	96.35	76.79	75.25	95.28	I	II	no	60.3
NPDC023112	NPDC046888	96.01	76.43	73.29	94.96	I	V	no	57.9
NPDC023112	NPDC047015	96.03	75.85	75.40	94.99	I	V	no	58.1
NPDC023112	NPDC052111	96.11	76.95	75.82	95.07	I	VI	no	58.0
NPDC023112	NPDC057006	96.32	79.56	75.05	95.24	I	III	no	59.5
NPDC023112	NPDC088830	99.47	91.89	91.17	99.10	I	I	yes	92.2
NPDC023112	NPDC101287	96.34	78.54	74.32	95.19	I	III	no	59.2

NPDC023112	NPDC127097	96.32	78.77	76.99	95.28	I	III	no	59.5
NPDC023112	TP-A0598	99.46	92.24	92.47	99.18	I	I	yes	92.8
NPDC024095	NPDC024428	98.85	90.05	91.40	98.35	I	I	yes	85.6
NPDC024095	NPDC025141	98.87	89.92	89.67	98.38	I	I	yes	85.7
NPDC024095	NPDC025256	96.29	77.94	74.43	95.28	I	II	no	59.7
NPDC024095	NPDC025511	98.85	89.92	86.86	98.31	I	I	yes	85.7
NPDC024095	NPDC025659	96.30	76.13	76.40	95.10	I	II	no	59.3
NPDC024095	NPDC029235	98.88	90.33	90.73	98.38	I	I	yes	86.0
NPDC024095	NPDC029466	97.39	83.61	82.05	96.57	I	VII	no	68.3
NPDC024095	NPDC029498	96.13	76.95	74.66	95.09	I	VI	no	58.0
NPDC024095	NPDC030177	98.88	89.64	93.36	98.46	I	I	yes	86.1
NPDC024095	NPDC030806	98.86	90.07	92.04	98.45	I	I	yes	86.2
NPDC024095	NPDC032166	98.85	89.98	87.14	98.36	I	I	yes	85.9
NPDC024095	NPDC032794	96.27	77.93	74.59	95.31	I	II	no	60.0
NPDC024095	NPDC032847	96.27	77.92	78.23	95.29	I	II	no	59.9
NPDC024095	NPDC032947	98.87	90.46	93.19	98.47	I	I	yes	86.5
NPDC024095	NPDC033176	96.80	79.74	81.87	95.96	I	VIII	no	64.4
NPDC024095	NPDC033267	96.23	79.03	73.95	95.25	I	III	no	58.7
NPDC024095	NPDC033460	98.89	90.41	92.27	98.43	I	I	yes	85.9
NPDC024095	NPDC033730	98.91	90.29	89.63	98.46	I	I	yes	86.6
NPDC024095	NPDC034584	96.27	77.79	75.37	95.30	I	II	no	59.8
NPDC024095	NPDC035741	98.92	91.53	88.92	98.41	I	I	yes	86.0
NPDC024095	NPDC039428	98.89	90.43	90.80	98.44	I	I	yes	86.4
NPDC024095	NPDC041037	96.04	77.32	74.72	95.05	I	V	no	58.2
NPDC024095	NPDC042329	96.20	77.94	78.19	95.32	I	II	no	59.8
NPDC024095	NPDC044766	96.30	77.10	77.41	95.30	I	II	no	60.0
NPDC024095	NPDC046625	96.29	77.13	75.74	95.22	I	II	no	60.0
NPDC024095	NPDC046888	95.90	76.29	73.30	94.88	I	V	no	57.8
NPDC024095	NPDC047015	95.99	75.93	75.63	94.92	I	V	no	57.8
NPDC024095	NPDC052111	96.07	77.21	76.23	95.08	I	VI	no	57.9
NPDC024095	NPDC057006	96.29	79.50	75.14	95.29	I	III	no	58.8
NPDC024095	NPDC088830	98.86	90.14	89.61	98.39	I	I	yes	85.9
NPDC024095	NPDC101287	96.31	79.73	75.60	95.19	I	III	no	58.7
NPDC024095	NPDC127097	96.27	78.69	77.07	95.24	I	III	no	58.8
NPDC024095	TP-A0598	98.84	90.26	90.67	98.40	I	I	yes	85.8
NPDC024428	NPDC025141	99.64	95.59	93.93	99.42	I	I	yes	95.1
NPDC024428	NPDC025256	96.32	78.20	73.58	95.22	I	II	no	59.9
NPDC024428	NPDC025511	99.66	95.64	91.03	99.35	I	I	yes	94.5
NPDC024428	NPDC025659	96.29	77.40	76.53	95.16	I	II	no	59.8
NPDC024428	NPDC029235	99.44	93.15	92.18	99.09	I	I	yes	92.3
NPDC024428	NPDC029466	97.44	85.85	83.01	96.67	I	VII	no	69.0
NPDC024428	NPDC029498	96.16	78.11	74.67	95.07	I	VI	no	58.1
NPDC024428	NPDC030177	99.66	94.87	97.34	99.47	I	I	yes	95.8

NPDC024428	NPDC030806	99.66	95.32	95.97	99.46	I	I	yes	95.5
NPDC024428	NPDC032166	99.64	95.58	91.20	99.41	I	I	yes	95.1
NPDC024428	NPDC032794	96.30	78.27	73.82	95.27	I	II	no	60.0
NPDC024428	NPDC032847	96.25	78.39	77.55	95.28	I	II	no	60.0
NPDC024428	NPDC032947	99.43	93.09	94.49	99.11	I	I	yes	92.0
NPDC024428	NPDC033176	96.83	80.26	81.19	95.88	I	VIII	no	64.5
NPDC024428	NPDC033267	96.33	80.40	74.12	95.32	I	III	no	59.3
NPDC024428	NPDC033460	99.43	93.36	93.87	99.12	I	I	yes	92.3
NPDC024428	NPDC033730	99.42	93.43	91.39	99.06	I	I	yes	91.7
NPDC024428	NPDC034584	96.33	78.20	74.65	95.20	I	II	no	60.0
NPDC024428	NPDC035741	99.44	93.53	89.53	99.08	I	I	yes	91.8
NPDC024428	NPDC039428	99.44	93.18	92.17	99.08	I	I	yes	92.2
NPDC024428	NPDC041037	96.13	77.83	74.11	95.00	I	V	no	58.5
NPDC024428	NPDC042329	96.26	78.36	77.45	95.24	I	II	no	60.0
NPDC024428	NPDC044766	96.31	78.14	77.30	95.24	I	II	no	60.1
NPDC024428	NPDC046625	96.32	77.89	75.36	95.21	I	II	no	60.2
NPDC024428	NPDC046888	96.08	77.36	73.24	95.02	I	V	no	58.1
NPDC024428	NPDC047015	96.03	77.23	75.79	95.01	I	V	no	58.0
NPDC024428	NPDC052111	96.02	78.12	76.00	95.08	I	VI	no	57.9
NPDC024428	NPDC057006	96.34	81.68	76.07	95.33	I	III	no	59.4
NPDC024428	NPDC088830	99.66	95.45	93.50	99.41	I	I	yes	95.1
NPDC024428	NPDC101287	96.33	80.75	75.45	95.23	I	III	no	59.2
NPDC024428	NPDC127097	96.30	80.18	77.36	95.33	I	III	no	59.4
NPDC024428	TP-A0598	99.64	94.62	93.65	99.34	I	I	yes	94.8
NPDC025141	NPDC025256	96.36	77.69	74.39	95.21	I	II	no	59.6
NPDC025141	NPDC025511	99.66	94.33	91.38	99.45	I	I	yes	95.0
NPDC025141	NPDC025659	96.29	75.92	76.41	95.17	I	II	no	59.6
NPDC025141	NPDC029235	99.41	91.87	92.53	99.08	I	I	yes	92.1
NPDC025141	NPDC029466	97.51	84.42	83.07	96.60	I	VII	no	68.6
NPDC025141	NPDC029498	96.18	76.52	74.45	95.03	I	VI	no	57.8
NPDC025141	NPDC030177	99.66	93.87	98.03	99.48	I	I	yes	95.4
NPDC025141	NPDC030806	99.75	94.26	96.59	99.58	I	I	yes	96.4
NPDC025141	NPDC032166	100.00	96.49	93.70	99.93	I	I	yes	99.6
NPDC025141	NPDC032794	96.31	76.93	73.84	95.23	I	II	no	60.0
NPDC025141	NPDC032847	96.28	77.20	77.73	95.30	I	II	no	60.0
NPDC025141	NPDC032947	99.43	91.37	94.39	99.14	I	I	yes	92.0
NPDC025141	NPDC033176	96.80	78.91	81.25	95.93	I	VIII	no	64.6
NPDC025141	NPDC033267	96.26	79.77	74.85	95.33	I	III	no	58.9
NPDC025141	NPDC033460	99.43	91.71	93.86	99.05	I	I	yes	91.9
NPDC025141	NPDC033730	99.42	91.81	91.39	99.06	I	I	yes	91.5
NPDC025141	NPDC034584	96.22	77.40	75.20	95.21	I	II	no	59.8
NPDC025141	NPDC035741	99.43	91.95	89.57	99.06	I	I	yes	91.5
NPDC025141	NPDC039428	99.41	91.93	92.56	99.07	I	I	yes	91.9

NPDC025141	NPDC041037	96.08	76.92	74.54	95.05	I	V	no	58.2
NPDC025141	NPDC042329	96.32	77.03	77.49	95.30	I	II	no	59.9
NPDC025141	NPDC044766	96.27	77.02	77.54	95.26	I	II	no	59.9
NPDC025141	NPDC046625	96.33	76.73	75.56	95.22	I	II	no	60.2
NPDC025141	NPDC046888	95.90	76.69	73.90	94.92	I	V	no	57.7
NPDC025141	NPDC047015	95.92	76.44	76.36	95.04	I	V	no	58.0
NPDC025141	NPDC052111	96.10	77.23	76.47	95.10	I	VI	no	58.0
NPDC025141	NPDC057006	96.27	81.24	77.01	95.34	I	III	no	59.2
NPDC025141	NPDC088830	99.66	95.28	94.99	99.34	I	I	yes	94.5
NPDC025141	NPDC101287	96.35	80.00	76.07	95.17	I	III	no	59.0
NPDC025141	NPDC127097	96.28	79.21	77.79	95.38	I	III	no	59.4
NPDC025141	TP-A0598	99.67	95.13	95.83	99.42	I	I	yes	94.9
NPDC025256	NPDC025511	96.26	74.97	75.84	95.23	II	I	no	59.6
NPDC025256	NPDC025659	98.85	84.09	88.38	98.32	II	II	yes	85.9
NPDC025256	NPDC029235	96.25	73.89	77.72	95.32	II	I	no	
NPDC025256	NPDC029466	96.11	71.92	73.91	94.99	II	VII	no	57.5
NPDC025256	NPDC029498	96.35	73.78	74.96	95.31	II	VI	no	59.9
NPDC025256	NPDC030177	96.37	73.99	80.69	95.26	II	I	no	
NPDC025256	NPDC030806	96.38	74.18	79.38	95.30	II	I	no	
NPDC025256	NPDC032166	96.23	74.29	75.33	95.20	II	I	no	59.6
NPDC025256	NPDC032794	99.68	92.29	92.51	99.50	II	II	yes	96.1
NPDC025256	NPDC032847	99.47	89.27	93.86	99.17	II	II	yes	93.1
NPDC025256	NPDC032947	96.30	74.13	79.97	95.32	II	I	no	60.0
NPDC025256	NPDC033176	96.46	73.99	79.56	95.55	II	VIII	no	
NPDC025256	NPDC033267	95.97	72.70	71.24	94.91	II	III	no	57.1
NPDC025256	NPDC033460	96.29	74.25	79.35	95.31	II	I	no	59.9
NPDC025256	NPDC033730	96.23	74.23	77.17	95.25	II	I	no	60.0
NPDC025256	NPDC034584	99.40	89.47	90.78	99.02	II	II	yes	92.1
NPDC025256	NPDC035741	96.24	74.16	75.45	95.26	II	I	no	
NPDC025256	NPDC039428	96.24	74.46	78.29	95.25	II	I	no	
NPDC025256	NPDC041037	97.40	80.77	81.74	96.54	II	V	no	69.3
NPDC025256	NPDC042329	99.38	89.40	93.92	99.09	II	II	yes	92.1
NPDC025256	NPDC044766	99.38	89.05	93.63	99.05	II	II	yes	92.1
NPDC025256	NPDC046625	99.65	91.92	94.53	99.39	II	II	yes	95.4
NPDC025256	NPDC046888	97.16	78.03	78.52	96.16	II	V	no	66.6
NPDC025256	NPDC047015	97.21	77.54	80.88	96.29	II	V	no	67.7
NPDC025256	NPDC052111	96.29	73.80	76.31	95.33	II	VI	no	59.8
NPDC025256	NPDC057006	95.97	73.71	72.96	94.77	II	III	no	56.8
NPDC025256	NPDC088830	96.36	74.53	77.60	95.21	II	I	no	
NPDC025256	NPDC101287	95.92	73.04	72.53	94.73	II	III	no	56.8
NPDC025256	NPDC127097	95.96	72.77	74.63	94.88	II	III	no	56.9
NPDC025256	TP-A0598	96.30	74.04	77.89	95.27	II	I	no	
NPDC025511	NPDC025659	96.32	73.45	76.31	95.18	I	II	no	59.7

NPDC025511	NPDC029235	99.44	90.06	93.64	99.04	I	I	yes	92.0
NPDC025511	NPDC029466	97.48	81.64	82.93	96.60	I	VII	no	68.9
NPDC025511	NPDC029498	96.15	74.40	74.73	95.07	I	VI	no	58.3
NPDC025511	NPDC030177	99.65	90.27	97.32	99.47	I	I	yes	95.6
NPDC025511	NPDC030806	100.00	92.66	98.02	99.87	I	I	yes	99.0
NPDC025511	NPDC032166	99.66	91.16	91.39	99.39	I	I	yes	94.7
NPDC025511	NPDC032794	96.29	75.00	74.32	95.18	I	II	no	59.8
NPDC025511	NPDC032847	96.25	75.08	78.03	95.28	I	II	no	60.0
NPDC025511	NPDC032947	99.44	88.49	94.37	99.11	I	I	yes	92.1
NPDC025511	NPDC033176	96.87	76.23	81.03	95.95	I	VIII	no	64.7
NPDC025511	NPDC033267	96.25	76.80	74.40	95.36	I	III	no	59.3
NPDC025511	NPDC033460	99.42	89.02	94.05	99.08	I	I	yes	91.8
NPDC025511	NPDC033730	99.42	89.97	92.46	99.08	I	I	yes	91.6
NPDC025511	NPDC034584	96.23	74.99	75.21	95.17	I	II	no	59.8
NPDC025511	NPDC035741	99.43	89.32	89.83	98.99	I	I	yes	91.0
NPDC025511	NPDC039428	99.43	89.02	92.53	98.96	I	I	yes	91.1
NPDC025511	NPDC041037	96.05	74.51	74.54	95.04	I	V	no	58.4
NPDC025511	NPDC042329	96.24	74.83	77.70	95.19	I	II	no	59.9
NPDC025511	NPDC044766	96.33	74.73	77.67	95.19	I	II	no	60.0
NPDC025511	NPDC046625	96.33	73.96	75.18	95.16	I	II	no	60.0
NPDC025511	NPDC046888	96.00	74.12	73.73	94.98	I	V	no	58.0
NPDC025511	NPDC047015	95.99	74.15	76.46	95.05	I	V	no	58.1
NPDC025511	NPDC052111	96.10	74.33	75.98	95.04	I	VI	no	57.9
NPDC025511	NPDC057006	96.28	77.84	76.17	95.31	I	III	no	59.1
NPDC025511	NPDC088830	100.00	93.62	96.35	99.97	I	I	yes	99.7
NPDC025511	NPDC101287	96.37	77.10	75.68	95.25	I	III	no	59.3
NPDC025511	NPDC127097	96.24	77.31	78.38	95.29	I	III	no	59.3
NPDC025511	TP-A0598	99.65	90.68	94.31	99.43	I	I	yes	95.3
NPDC025659	NPDC029235	96.32	76.16	76.22	95.15	II	I	no	
NPDC025659	NPDC029466	96.03	75.26	73.59	94.91	II	VII	no	57.4
NPDC025659	NPDC029498	96.29	76.36	73.82	95.16	II	VI	no	59.9
NPDC025659	NPDC030177	96.30	76.26	79.14	95.20	II	I	no	
NPDC025659	NPDC030806	96.32	76.34	77.73	95.20	II	I	no	
NPDC025659	NPDC032166	96.33	76.91	74.21	95.20	II	I	no	59.8
NPDC025659	NPDC032794	98.87	88.96	84.84	98.29	II	II	yes	85.6
NPDC025659	NPDC032847	98.87	88.10	88.14	98.27	II	II	yes	85.4
NPDC025659	NPDC032947	96.34	76.14	78.16	95.19	II	I	no	59.6
NPDC025659	NPDC033176	96.48	76.03	77.78	95.42	II	VIII	no	
NPDC025659	NPDC033267	96.05	74.91	69.84	94.83	II	III	no	57.0
NPDC025659	NPDC033460	96.30	76.34	77.63	95.14	II	I	no	59.4
NPDC025659	NPDC033730	96.31	76.34	75.51	95.17	II	I	no	59.5
NPDC025659	NPDC034584	98.86	87.71	84.67	98.24	II	II	yes	85.8
NPDC025659	NPDC035741	96.31	76.42	73.97	95.13	II	I	no	

NPDC025659	NPDC039428	96.28	76.35	76.38	95.14	II	I	no	
NPDC025659	NPDC041037	97.34	83.13	80.04	96.35	II	V	no	68.7
NPDC025659	NPDC042329	98.88	87.85	87.81	98.26	II	II	yes	85.5
NPDC025659	NPDC044766	98.84	87.64	87.67	98.22	II	II	yes	85.5
NPDC025659	NPDC046625	98.88	88.54	86.63	98.21	II	II	yes	85.5
NPDC025659	NPDC046888	97.13	80.65	77.22	96.21	II	V	no	67.2
NPDC025659	NPDC047015	97.19	80.35	79.75	96.16	II	V	no	67.2
NPDC025659	NPDC052111	96.27	76.71	75.47	95.15	II	VI	no	59.7
NPDC025659	NPDC057006	96.08	75.67	71.27	94.79	II	III	no	57.0
NPDC025659	NPDC088830	96.32	76.44	75.72	95.16	II	I	no	
NPDC025659	NPDC101287	96.14	75.21	71.07	94.79	II	III	no	57.0
NPDC025659	NPDC127097	96.06	75.47	73.64	94.86	II	III	no	57.1
NPDC025659	TP-A0598	96.29	76.41	76.48	95.12	II	I	no	
NPDC029235	NPDC029466	97.47	85.06	83.11	96.56	I	VII	no	68.3
NPDC029235	NPDC029498	96.15	77.34	74.71	95.07	I	VI	no	
NPDC029235	NPDC030177	99.41	92.40	95.81	99.13	I	I	yes	92.3
NPDC029235	NPDC030806	99.42	92.37	93.98	99.12	I	I	yes	92.5
NPDC029235	NPDC032166	99.42	92.34	89.03	99.07	I	I	yes	92.3
NPDC029235	NPDC032794	96.28	77.86	74.20	95.25	I	II	no	
NPDC029235	NPDC032847	96.26	78.10	78.07	95.31	I	II	no	
NPDC029235	NPDC032947	99.70	95.14	97.58	99.51	I	I	yes	95.7
NPDC029235	NPDC033176	96.77	79.19	80.96	95.91	I	VIII	no	64.4
NPDC029235	NPDC033267	96.27	79.50	74.06	95.28	I	III	no	58.9
NPDC029235	NPDC033460	99.69	94.54	96.06	99.46	I	I	yes	95.7
NPDC029235	NPDC033730	99.67	96.10	94.98	99.44	I	I	yes	95.3
NPDC029235	NPDC034584	96.22	77.91	75.15	95.28	I	II	no	
NPDC029235	NPDC035741	99.67	94.97	91.85	99.35	I	I	yes	94.7
NPDC029235	NPDC039428	99.66	94.85	94.81	99.37	I	I	yes	94.6
NPDC029235	NPDC041037	96.12	78.02	75.07	95.07	I	V	no	
NPDC029235	NPDC042329	96.23	78.15	78.05	95.31	I	II	no	
NPDC029235	NPDC044766	96.27	77.11	77.08	95.28	I	II	no	
NPDC029235	NPDC046625	96.32	77.44	75.71	95.23	I	II	no	
NPDC029235	NPDC046888	95.94	76.91	73.57	94.95	I	V	no	
NPDC029235	NPDC047015	95.96	76.29	75.66	94.98	I	V	no	
NPDC029235	NPDC052111	96.09	77.15	75.84	95.08	I	VI	no	
NPDC029235	NPDC057006	96.30	80.56	75.82	95.31	I	III	no	59.1
NPDC029235	NPDC088830	99.42	92.37	91.43	99.06	I	I	yes	92.2
NPDC029235	NPDC101287	96.29	79.25	74.82	95.23	I	III	no	59.0
NPDC029235	NPDC127097	96.29	79.54	77.56	95.30	I	III	no	59.4
NPDC029235	TP-A0598	99.41	92.81	92.83	99.12	I	I	yes	92.4
NPDC029466	NPDC029498	95.89	75.84	74.98	94.84	VII	VI	no	56.1
NPDC029466	NPDC030177	97.48	83.35	88.45	96.72	VII	I	no	69.1
NPDC029466	NPDC030806	97.50	83.02	86.45	96.67	VII	I	no	69.1

NPDC029466	NPDC032166	97.42	83.28	82.17	96.58	VII	I	no	68.5
NPDC029466	NPDC032794	96.06	74.09	72.27	95.04	VII	II	no	58.0
NPDC029466	NPDC032847	96.05	74.40	76.11	94.97	VII	II	no	57.8
NPDC029466	NPDC032947	97.45	82.38	86.48	96.63	VII	I	no	68.7
NPDC029466	NPDC033176	96.47	76.04	79.56	95.57	VII	VIII	no	61.1
NPDC029466	NPDC033267	96.19	78.58	74.93	95.18	VII	III	no	58.0
NPDC029466	NPDC033460	97.46	82.89	86.20	96.58	VII	I	no	68.5
NPDC029466	NPDC033730	97.48	82.56	83.52	96.62	VII	I	no	68.7
NPDC029466	NPDC034584	96.07	74.28	73.33	94.96	VII	II	no	57.7
NPDC029466	NPDC035741	97.51	82.68	81.85	96.55	VII	I	no	68.4
NPDC029466	NPDC039428	97.47	82.49	84.40	96.60	VII	I	no	68.6
NPDC029466	NPDC041037	95.91	75.49	74.34	94.78	VII	V	no	56.0
NPDC029466	NPDC042329	96.06	74.39	76.04	95.02	VII	II	no	57.8
NPDC029466	NPDC044766	96.04	74.24	75.96	94.97	VII	II	no	57.9
NPDC029466	NPDC046625	96.01	74.01	74.06	94.95	VII	II	no	57.8
NPDC029466	NPDC046888	95.94	75.20	73.63	94.77	VII	V	no	56.1
NPDC029466	NPDC047015	95.89	75.26	76.39	94.75	VII	V	no	56.0
NPDC029466	NPDC052111	95.93	76.20	76.67	94.86	VII	VI	no	55.9
NPDC029466	NPDC057006	96.25	79.60	76.66	95.31	VII	III	no	58.6
NPDC029466	NPDC088830	97.47	83.37	84.46	96.61	VII	I	no	68.6
NPDC029466	NPDC101287	96.25	79.11	76.44	95.19	VII	III	no	58.3
NPDC029466	NPDC127097	96.12	78.65	78.49	95.29	VII	III	no	58.5
NPDC029466	TP-A0598	97.49	83.27	85.24	96.67	VII	I	no	68.8
NPDC029498	NPDC030177	96.16	74.46	79.93	95.11	VI	I	no	
NPDC029498	NPDC030806	96.17	74.76	78.74	95.11	VI	I	no	
NPDC029498	NPDC032166	96.16	74.72	74.58	94.94	VI	I	no	57.4
NPDC029498	NPDC032794	96.32	74.71	73.71	95.25	VI	II	no	60.0
NPDC029498	NPDC032847	96.26	75.45	78.07	95.30	VI	II	no	60.0
NPDC029498	NPDC032947	96.17	74.88	79.51	95.12	VI	I	no	58.0
NPDC029498	NPDC033176	96.23	73.30	77.57	95.14	VI	VIII	no	
NPDC029498	NPDC033267	95.87	72.51	69.93	94.74	VI	III	no	55.4
NPDC029498	NPDC033460	96.10	74.86	78.74	95.12	VI	I	no	58.0
NPDC029498	NPDC033730	96.17	74.84	76.57	95.11	VI	I	no	58.1
NPDC029498	NPDC034584	96.32	75.29	75.18	95.23	VI	II	no	59.9
NPDC029498	NPDC035741	96.16	74.85	74.94	95.01	VI	I	no	
NPDC029498	NPDC039428	96.15	74.89	77.49	95.05	VI	I	no	
NPDC029498	NPDC041037	96.24	75.26	74.96	95.10	VI	V	no	58.8
NPDC029498	NPDC042329	96.29	75.10	77.65	95.30	VI	II	no	60.2
NPDC029498	NPDC044766	96.27	74.27	76.86	95.19	VI	II	no	60.1
NPDC029498	NPDC046625	96.28	74.16	75.06	95.25	VI	II	no	60.2
NPDC029498	NPDC046888	96.16	75.63	74.90	95.04	VI	V	no	58.2
NPDC029498	NPDC047015	96.15	75.49	77.50	95.05	VI	V	no	58.1
NPDC029498	NPDC052111	99.42	92.52	94.16	99.12	VI	VI	yes	92.2

NPDC029498	NPDC057006	95.87	73.45	71.56	94.76	VI	III	no	55.5
NPDC029498	NPDC088830	96.15	75.19	77.04	95.13	VI	I	no	
NPDC029498	NPDC101287	95.84	73.52	71.86	94.67	VI	III	no	55.6
NPDC029498	NPDC127097	95.86	72.67	73.35	94.68	VI	III	no	55.5
NPDC029498	TP-A0598	96.12	74.93	77.58	95.08	VI	I	no	
NPDC030177	NPDC030806	99.65	97.45	95.62	99.50	I	I	yes	95.6
NPDC030177	NPDC032166	99.65	97.90	91.03	99.46	I	I	yes	95.3
NPDC030177	NPDC032794	96.29	80.43	73.92	95.35	I	II	no	
NPDC030177	NPDC032847	96.30	80.51	77.62	95.35	I	II	no	
NPDC030177	NPDC032947	99.43	95.54	94.50	99.14	I	I	yes	92.4
NPDC030177	NPDC033176	96.83	82.32	81.17	95.98	I	VIII	no	64.7
NPDC030177	NPDC033267	96.29	83.17	74.73	95.39	I	III	no	59.3
NPDC030177	NPDC033460	99.42	95.37	93.46	99.13	I	I	yes	92.4
NPDC030177	NPDC033730	99.42	95.49	91.02	99.15	I	I	yes	92.4
NPDC030177	NPDC034584	96.24	81.01	75.36	95.31	I	II	no	
NPDC030177	NPDC035741	99.45	95.70	89.27	99.14	I	I	yes	92.1
NPDC030177	NPDC039428	99.45	95.68	92.25	99.15	I	I	yes	92.5
NPDC030177	NPDC041037	96.09	80.80	74.98	95.12	I	V	no	
NPDC030177	NPDC042329	96.33	80.55	77.58	95.33	I	II	no	
NPDC030177	NPDC044766	96.28	80.52	77.63	95.31	I	II	no	
NPDC030177	NPDC046625	96.34	79.99	75.43	95.26	I	II	no	
NPDC030177	NPDC046888	95.95	78.97	72.86	95.06	I	V	no	
NPDC030177	NPDC047015	95.98	79.00	75.56	95.06	I	V	no	
NPDC030177	NPDC052111	96.12	80.13	75.97	95.15	I	VI	no	
NPDC030177	NPDC057006	96.24	84.22	76.44	95.41	I	III	no	59.3
NPDC030177	NPDC088830	99.65	97.47	93.04	99.47	I	I	yes	95.6
NPDC030177	NPDC101287	96.40	83.01	75.58	95.29	I	III	no	59.3
NPDC030177	NPDC127097	96.22	82.81	77.88	95.38	I	III	no	59.5
NPDC030177	TP-A0598	99.64	97.33	93.89	99.45	I	I	yes	95.4
NPDC030806	NPDC032166	99.75	96.10	91.07	99.55	I	I	yes	96.5
NPDC030806	NPDC032794	96.34	78.94	73.95	95.35	I	II	no	
NPDC030806	NPDC032847	96.29	79.30	77.91	95.36	I	II	no	
NPDC030806	NPDC032947	99.44	93.65	94.41	99.14	I	I	yes	92.5
NPDC030806	NPDC033176	96.84	80.88	81.27	95.96	I	VIII	no	64.8
NPDC030806	NPDC033267	96.28	81.46	74.59	95.39	I	III	no	59.3
NPDC030806	NPDC033460	99.43	94.04	93.92	99.14	I	I	yes	92.4
NPDC030806	NPDC033730	99.43	93.88	91.20	99.12	I	I	yes	92.5
NPDC030806	NPDC034584	96.24	79.27	75.15	95.26	I	II	no	
NPDC030806	NPDC035741	99.45	94.29	89.64	99.14	I	I	yes	92.3
NPDC030806	NPDC039428	99.44	94.14	92.50	99.10	I	I	yes	92.3
NPDC030806	NPDC041037	96.16	78.61	74.34	95.17	I	V	no	
NPDC030806	NPDC042329	96.31	79.04	77.59	95.32	I	II	no	
NPDC030806	NPDC044766	96.32	78.78	77.40	95.25	I	II	no	

NPDC030806	NPDC046625	96.33	78.27	75.22	95.24	I	II	no	
NPDC030806	NPDC046888	95.96	77.96	73.31	95.04	I	V	no	
NPDC030806	NPDC047015	95.95	77.95	75.98	95.07	I	V	no	
NPDC030806	NPDC052111	96.08	78.76	76.10	95.11	I	VI	no	
NPDC030806	NPDC057006	96.27	82.47	76.28	95.41	I	III	no	59.3
NPDC030806	NPDC088830	100.00	98.17	95.50	99.85	I	I	yes	99.0
NPDC030806	NPDC101287	96.39	81.44	75.58	95.29	I	III	no	59.2
NPDC030806	NPDC127097	96.28	81.06	77.69	95.41	I	III	no	59.6
NPDC030806	TP-A0598	99.64	96.05	94.42	99.46	I	I	yes	95.7
NPDC032166	NPDC032794	96.27	74.67	73.81	95.25	I	II	no	60.1
NPDC032166	NPDC032847	96.27	74.89	77.65	95.33	I	II	no	60.2
NPDC032166	NPDC032947	99.43	88.91	94.59	99.12	I	I	yes	92.1
NPDC032166	NPDC033176	96.80	76.78	81.41	95.83	I	VIII	no	64.2
NPDC032166	NPDC033267	96.27	76.68	74.09	95.29	I	III	no	59.1
NPDC032166	NPDC033460	99.41	89.03	93.83	99.12	I	I	yes	92.3
NPDC032166	NPDC033730	99.40	89.23	91.47	99.06	I	I	yes	91.7
NPDC032166	NPDC034584	96.29	75.06	75.10	95.23	I	II	no	59.9
NPDC032166	NPDC035741	99.40	89.31	89.59	99.08	I	I	yes	91.6
NPDC032166	NPDC039428	99.43	89.21	92.50	99.07	I	I	yes	92.0
NPDC032166	NPDC041037	96.13	74.62	74.46	95.12	I	V	no	58.4
NPDC032166	NPDC042329	96.29	74.96	77.65	95.27	I	II	no	60.0
NPDC032166	NPDC044766	96.27	74.54	77.28	95.28	I	II	no	60.0
NPDC032166	NPDC046625	96.33	74.26	75.31	95.23	I	II	no	60.2
NPDC032166	NPDC046888	96.08	75.00	74.42	95.07	I	V	no	58.3
NPDC032166	NPDC047015	96.03	74.02	76.13	95.00	I	V	no	57.9
NPDC032166	NPDC052111	96.14	74.85	76.32	95.14	I	VI	no	58.0
NPDC032166	NPDC057006	96.30	78.42	76.54	95.24	I	III	no	58.8
NPDC032166	NPDC088830	99.66	91.26	93.69	99.41	I	I	yes	95.0
NPDC032166	NPDC101287	96.38	77.04	75.44	95.22	I	III	no	59.1
NPDC032166	NPDC127097	96.31	76.63	77.50	95.35	I	III	no	59.5
NPDC032166	TP-A0598	99.66	91.22	94.63	99.38	I	I	yes	94.9
NPDC032794	NPDC032847	99.48	89.15	93.51	99.20	II	II	yes	93.3
NPDC032794	NPDC032947	96.31	74.11	79.76	95.34	II	I	no	60.3
NPDC032794	NPDC033176	96.48	74.25	79.65	95.56	II	VIII	no	
NPDC032794	NPDC033267	96.01	72.86	71.23	94.90	II	III	no	57.1
NPDC032794	NPDC033460	96.27	74.15	79.06	95.31	II	I	no	60.0
NPDC032794	NPDC033730	96.24	74.26	77.01	95.32	II	I	no	60.2
NPDC032794	NPDC034584	99.38	89.07	90.15	99.02	II	II	yes	92.1
NPDC032794	NPDC035741	96.28	74.57	75.68	95.28	II	I	no	
NPDC032794	NPDC039428	96.28	74.22	77.85	95.32	II	I	no	
NPDC032794	NPDC041037	97.33	80.86	81.63	96.46	II	V	no	69.2
NPDC032794	NPDC042329	99.40	89.73	94.04	99.06	II	II	yes	92.1
NPDC032794	NPDC044766	99.38	89.40	93.78	99.08	II	II	yes	92.2

NPDC032794	NPDC046625	99.68	92.32	94.71	99.47	II	II	yes	96.0
NPDC032794	NPDC046888	97.23	77.86	78.16	96.25	II	V	no	67.5
NPDC032794	NPDC047015	97.25	77.64	80.79	96.29	II	V	no	67.8
NPDC032794	NPDC052111	96.26	73.70	76.02	95.32	II	VI	no	59.8
NPDC032794	NPDC057006	96.00	73.16	72.24	94.85	II	III	no	57.0
NPDC032794	NPDC088830	96.29	74.14	77.00	95.24	II	I	no	
NPDC032794	NPDC101287	95.96	73.08	72.40	94.84	II	III	no	57.1
NPDC032794	NPDC127097	96.04	72.71	74.39	94.92	II	III	no	57.1
NPDC032794	TP-A0598	96.34	74.16	77.82	95.25	II	I	no	
NPDC032847	NPDC032947	96.24	78.21	80.25	95.37	II	I	no	60.2
NPDC032847	NPDC033176	96.47	78.30	80.08	95.59	II	VIII	no	
NPDC032847	NPDC033267	96.01	76.74	71.52	94.99	II	III	no	57.1
NPDC032847	NPDC033460	96.24	78.33	79.62	95.35	II	I	no	60.1
NPDC032847	NPDC033730	96.27	78.03	77.15	95.33	II	I	no	60.2
NPDC032847	NPDC034584	99.37	92.48	89.24	98.97	II	II	yes	91.1
NPDC032847	NPDC035741	96.30	78.46	75.91	95.29	II	I	no	
NPDC032847	NPDC039428	96.26	78.15	78.15	95.31	II	I	no	
NPDC032847	NPDC041037	97.39	84.65	81.47	96.58	II	V	no	69.4
NPDC032847	NPDC042329	99.37	93.45	93.37	98.97	II	II	yes	91.3
NPDC032847	NPDC044766	99.33	92.77	92.77	98.99	II	II	yes	91.4
NPDC032847	NPDC046625	99.46	93.74	91.68	99.13	II	II	yes	93.0
NPDC032847	NPDC046888	97.18	81.89	78.37	96.32	II	V	no	67.5
NPDC032847	NPDC047015	97.20	81.75	81.10	96.30	II	V	no	67.3
NPDC032847	NPDC052111	96.28	78.24	76.94	95.33	II	VI	no	59.9
NPDC032847	NPDC057006	96.06	77.09	72.57	94.94	II	III	no	57.4
NPDC032847	NPDC088830	96.27	78.12	77.35	95.30	II	I	no	
NPDC032847	NPDC101287	95.92	77.44	73.14	94.88	II	III	no	57.0
NPDC032847	NPDC127097	95.90	76.50	74.62	94.95	II	III	no	57.1
NPDC032847	TP-A0598	96.26	78.10	78.14	95.30	II	I	no	
NPDC032947	NPDC033176	96.77	81.15	80.88	95.93	I	VIII	no	64.6
NPDC032947	NPDC033267	96.35	81.05	73.61	95.30	I	III	no	59.2
NPDC032947	NPDC033460	99.68	96.03	95.13	99.48	I	I	yes	95.4
NPDC032947	NPDC033730	99.67	97.64	94.09	99.52	I	I	yes	96.1
NPDC032947	NPDC034584	96.27	80.42	75.63	95.30	I	II	no	60.1
NPDC032947	NPDC035741	99.68	96.95	91.42	99.44	I	I	yes	95.5
NPDC032947	NPDC039428	99.70	97.39	94.92	99.52	I	I	yes	95.9
NPDC032947	NPDC041037	96.09	79.42	74.50	95.11	I	V	no	58.5
NPDC032947	NPDC042329	96.27	79.90	77.80	95.32	I	II	no	60.3
NPDC032947	NPDC044766	96.30	79.18	77.17	95.31	I	II	no	60.4
NPDC032947	NPDC046625	96.33	79.45	75.73	95.27	I	II	no	60.3
NPDC032947	NPDC046888	96.11	78.57	73.28	95.00	I	V	no	58.1
NPDC032947	NPDC047015	96.11	78.51	75.91	95.01	I	V	no	58.0
NPDC032947	NPDC052111	96.02	79.13	75.85	95.09	I	VI	no	57.9

NPDC032947	NPDC057006	96.35	81.79	75.04	95.35	I	III	no	59.3
NPDC032947	NPDC088830	99.45	94.39	91.09	99.12	I	I	yes	92.1
NPDC032947	NPDC101287	96.35	81.44	74.96	95.27	I	III	no	59.2
NPDC032947	NPDC127097	96.29	81.05	77.05	95.36	I	III	no	59.4
NPDC032947	TP-A0598	99.42	94.50	92.15	99.16	I	I	yes	92.4
NPDC033176	NPDC033267	96.11	78.58	71.61	95.16	VIII	III	no	58.5
NPDC033176	NPDC033460	96.83	80.88	80.39	95.99	VIII	I	no	64.5
NPDC033176	NPDC033730	96.76	81.16	78.47	95.95	VIII	I	no	64.7
NPDC033176	NPDC034584	96.47	80.27	75.74	95.53	VIII	II	no	
NPDC033176	NPDC035741	96.81	81.61	77.21	95.92	VIII	I	no	64.5
NPDC033176	NPDC039428	96.86	81.45	79.65	95.94	VIII	I	no	64.5
NPDC033176	NPDC041037	96.28	80.25	75.53	95.30	VIII	V	no	
NPDC033176	NPDC042329	96.48	80.25	78.40	95.61	VIII	II	no	
NPDC033176	NPDC044766	96.48	79.30	77.54	95.57	VIII	II	no	
NPDC033176	NPDC046625	96.46	79.23	75.77	95.49	VIII	II	no	
NPDC033176	NPDC046888	96.14	78.49	73.45	95.14	VIII	V	no	
NPDC033176	NPDC047015	96.14	78.25	75.92	95.14	VIII	V	no	
NPDC033176	NPDC052111	96.21	78.08	75.09	95.23	VIII	VI	no	
NPDC033176	NPDC057006	96.15	78.65	72.40	95.17	VIII	III	no	58.7
NPDC033176	NPDC088830	96.84	81.28	78.69	95.91	VIII	I	no	64.5
NPDC033176	NPDC101287	96.24	78.27	72.28	95.04	VIII	III	no	58.7
NPDC033176	NPDC127097	96.13	77.86	74.26	95.17	VIII	III	no	58.7
NPDC033176	TP-A0598	96.82	81.42	79.66	95.95	VIII	I	no	64.8
NPDC033267	NPDC033460	96.32	73.68	80.36	95.28	III	I	no	59.0
NPDC033267	NPDC033730	96.23	74.92	79.48	95.24	III	I	no	59.0
NPDC033267	NPDC034584	95.95	72.25	74.81	94.84	III	II	no	56.8
NPDC033267	NPDC035741	96.25	74.25	77.08	95.21	III	I	no	58.8
NPDC033267	NPDC039428	96.26	74.40	79.83	95.25	III	I	no	59.1
NPDC033267	NPDC041037	95.76	71.98	74.34	94.66	III	V	no	55.2
NPDC033267	NPDC042329	95.97	71.96	77.14	94.95	III	II	no	57.3
NPDC033267	NPDC044766	95.98	71.57	76.80	94.84	III	II	no	57.1
NPDC033267	NPDC046625	96.00	70.63	74.12	94.85	III	II	no	57.3
NPDC033267	NPDC046888	95.76	71.31	73.23	94.72	III	V	no	55.9
NPDC033267	NPDC047015	95.79	71.08	75.67	94.75	III	V	no	56.0
NPDC033267	NPDC052111	95.80	70.29	74.17	94.70	III	VI	no	55.2
NPDC033267	NPDC057006	98.94	87.08	87.96	98.51	III	III	yes	84.9
NPDC033267	NPDC088830	96.26	74.70	79.37	95.29	III	I	no	59.0
NPDC033267	NPDC101287	98.75	87.10	88.27	98.26	III	III	yes	83.1
NPDC033267	NPDC127097	98.78	86.97	91.03	98.33	III	III	yes	83.5
NPDC033267	TP-A0598	96.30	74.60	80.09	95.29	III	I	no	59.1
NPDC033460	NPDC033730	99.67	95.27	92.67	99.38	I	I	yes	95.0
NPDC033460	NPDC034584	96.30	79.35	75.33	95.25	I	II	no	59.8
NPDC033460	NPDC035741	99.67	96.21	91.58	99.50	I	I	yes	95.2

NPDC033460	NPDC039428	99.68	95.52	93.97	99.46	I	I	yes	95.3
NPDC033460	NPDC041037	96.08	79.69	75.46	95.09	I	V	no	58.5
NPDC033460	NPDC042329	96.30	79.69	78.33	95.35	I	II	no	60.1
NPDC033460	NPDC044766	96.24	78.73	77.45	95.28	I	II	no	60.0
NPDC033460	NPDC046625	96.31	78.69	75.71	95.22	I	II	no	60.2
NPDC033460	NPDC046888	96.01	78.04	73.47	94.99	I	V	no	57.8
NPDC033460	NPDC047015	96.05	77.94	76.08	95.01	I	V	no	57.9
NPDC033460	NPDC052111	96.11	78.93	76.36	95.08	I	VI	no	57.9
NPDC033460	NPDC057006	96.26	81.44	75.43	95.33	I	III	no	59.2
NPDC033460	NPDC088830	99.42	94.05	91.62	99.07	I	I	yes	92.1
NPDC033460	NPDC101287	96.30	80.56	74.85	95.23	I	III	no	58.9
NPDC033460	NPDC127097	96.25	80.54	77.28	95.29	I	III	no	59.2
NPDC033460	TP-A0598	99.42	94.15	92.68	99.13	I	I	yes	92.1
NPDC033730	NPDC034584	96.25	77.89	76.01	95.30	I	II	no	60.1
NPDC033730	NPDC035741	99.69	93.62	91.62	99.39	I	I	yes	95.0
NPDC033730	NPDC039428	99.71	94.90	95.98	99.46	I	I	yes	95.7
NPDC033730	NPDC041037	96.04	76.89	74.85	95.11	I	V	no	58.5
NPDC033730	NPDC042329	96.26	77.13	77.94	95.32	I	II	no	60.2
NPDC033730	NPDC044766	96.28	76.83	77.71	95.27	I	II	no	60.3
NPDC033730	NPDC046625	96.34	76.49	75.66	95.25	I	II	no	60.4
NPDC033730	NPDC046888	96.00	75.77	73.34	94.97	I	V	no	57.9
NPDC033730	NPDC047015	96.01	75.50	75.75	95.04	I	V	no	58.0
NPDC033730	NPDC052111	96.09	76.66	76.25	95.01	I	VI	no	57.9
NPDC033730	NPDC057006	96.21	79.41	75.61	95.28	I	III	no	59.0
NPDC033730	NPDC088830	99.43	91.33	91.47	99.04	I	I	yes	91.7
NPDC033730	NPDC101287	96.33	78.89	75.36	95.25	I	III	no	58.9
NPDC033730	NPDC127097	96.25	78.51	77.45	95.33	I	III	no	59.3
NPDC033730	TP-A0598	99.44	91.28	92.38	99.09	I	I	yes	92.0
NPDC034584	NPDC035741	96.26	75.88	76.08	95.22	II	I	no	
NPDC034584	NPDC039428	96.24	75.75	78.50	95.30	II	I	no	
NPDC034584	NPDC041037	97.32	81.35	81.14	96.49	II	V	no	69.3
NPDC034584	NPDC042329	99.68	92.21	95.48	99.46	II	II	yes	95.8
NPDC034584	NPDC044766	99.66	93.18	96.56	99.43	II	II	yes	95.4
NPDC034584	NPDC046625	99.38	90.39	91.62	98.98	II	II	yes	92.0
NPDC034584	NPDC046888	97.13	78.88	78.23	96.21	II	V	no	67.4
NPDC034584	NPDC047015	97.11	78.65	80.86	96.28	II	V	no	67.9
NPDC034584	NPDC052111	96.27	75.87	77.32	95.35	II	VI	no	59.8
NPDC034584	NPDC057006	95.97	74.68	72.86	94.86	II	III	no	57.0
NPDC034584	NPDC088830	96.24	75.43	77.40	95.21	II	I	no	
NPDC034584	NPDC101287	96.05	74.78	73.19	94.79	II	III	no	56.8
NPDC034584	NPDC127097	95.88	73.98	74.78	94.87	II	III	no	56.9
NPDC034584	TP-A0598	96.27	75.20	77.97	95.22	II	I	no	
NPDC035741	NPDC039428	99.70	94.56	97.73	99.52	I	I	yes	95.7

NPDC035741	NPDC041037	96.14	74.82	74.43	95.05	I	V	no	
NPDC035741	NPDC042329	96.26	75.92	78.39	95.36	I	II	no	
NPDC035741	NPDC044766	96.29	74.92	77.43	95.29	I	II	no	
NPDC035741	NPDC046625	96.33	74.98	75.79	95.25	I	II	no	
NPDC035741	NPDC046888	95.92	74.13	73.33	94.90	I	V	no	
NPDC035741	NPDC047015	95.91	74.54	76.42	94.94	I	V	no	
NPDC035741	NPDC052111	96.14	75.29	76.52	95.02	I	VI	no	
NPDC035741	NPDC057006	96.26	78.26	76.15	95.22	I	III	no	58.9
NPDC035741	NPDC088830	99.44	89.65	91.75	99.08	I	I	yes	91.5
NPDC035741	NPDC101287	96.36	77.08	75.24	95.22	I	III	no	59.0
NPDC035741	NPDC127097	96.26	77.03	77.65	95.30	I	III	no	59.1
NPDC035741	TP-A0598	99.42	89.80	92.86	99.08	I	I	yes	92.0
NPDC039428	NPDC041037	96.08	77.67	74.76	95.09	I	V	no	
NPDC039428	NPDC042329	96.27	78.01	77.94	95.33	I	II	no	
NPDC039428	NPDC044766	96.30	77.38	77.38	95.31	I	II	no	
NPDC039428	NPDC046625	96.32	77.54	75.83	95.22	I	II	no	
NPDC039428	NPDC046888	95.92	76.84	73.54	94.99	I	V	no	
NPDC039428	NPDC047015	95.92	76.40	75.79	95.02	I	V	no	
NPDC039428	NPDC052111	96.16	77.49	76.21	95.07	I	VI	no	
NPDC039428	NPDC057006	96.22	80.78	76.05	95.28	I	III	no	59.0
NPDC039428	NPDC088830	99.45	92.37	91.46	99.05	I	I	yes	91.8
NPDC039428	NPDC101287	96.38	79.87	75.43	95.23	I	III	no	59.0
NPDC039428	NPDC127097	96.26	79.38	77.42	95.28	I	III	no	59.3
NPDC039428	TP-A0598	99.44	92.56	92.61	99.09	I	I	yes	92.3
NPDC041037	NPDC042329	97.29	81.43	84.53	96.51	V	II	no	69.4
NPDC041037	NPDC044766	97.35	81.22	84.38	96.48	V	II	no	69.5
NPDC041037	NPDC046625	97.30	81.17	82.48	96.41	V	II	no	69.3
NPDC041037	NPDC046888	97.80	82.42	81.95	96.91	V	V	yes	72.5
NPDC041037	NPDC047015	97.77	82.22	84.75	96.96	V	V	yes	72.8
NPDC041037	NPDC052111	96.15	75.53	77.17	95.20	V	VI	no	58.6
NPDC041037	NPDC057006	95.83	74.72	73.08	94.78	V	III	no	55.7
NPDC041037	NPDC088830	96.14	74.67	76.81	95.02	V	I	no	
NPDC041037	NPDC101287	95.80	74.31	72.92	94.70	V	III	no	55.7
NPDC041037	NPDC127097	95.78	73.88	74.88	94.69	V	III	no	55.7
NPDC041037	TP-A0598	96.01	74.49	77.43	95.09	V	I	no	
NPDC042329	NPDC044766	99.67	95.73	95.81	99.43	II	II	yes	95.9
NPDC042329	NPDC046625	99.38	93.92	91.94	99.03	II	II	yes	92.0
NPDC042329	NPDC046888	97.13	81.82	78.38	96.25	II	V	no	67.3
NPDC042329	NPDC047015	97.08	81.61	81.04	96.36	II	V	no	67.6
NPDC042329	NPDC052111	96.25	78.37	77.13	95.40	II	VI	no	59.9
NPDC042329	NPDC057006	95.93	77.73	73.24	94.88	II	III	no	57.0
NPDC042329	NPDC088830	96.31	77.78	77.09	95.28	II	I	no	
NPDC042329	NPDC101287	96.01	77.21	72.99	94.86	II	III	no	57.2

NPDC042329	NPDC127097	95.91	76.45	74.63	94.92	II	III	no	56.9
NPDC042329	TP-A0598	96.30	77.51	77.62	95.28	II	I	no	
NPDC044766	NPDC046625	99.38	93.35	91.30	99.03	II	II	yes	92.0
NPDC044766	NPDC046888	97.16	81.60	78.09	96.24	II	V	no	67.5
NPDC044766	NPDC047015	97.14	81.16	80.51	96.28	II	V	no	67.9
NPDC044766	NPDC052111	96.27	77.24	75.96	95.31	II	VI	no	59.8
NPDC044766	NPDC057006	96.01	76.73	72.23	94.86	II	III	no	57.2
NPDC044766	NPDC088830	96.32	77.91	77.15	95.20	II	I	no	
NPDC044766	NPDC101287	96.01	76.63	72.37	94.82	II	III	no	57.0
NPDC044766	NPDC127097	96.07	76.23	74.35	94.90	II	III	no	57.1
NPDC044766	TP-A0598	96.25	77.25	77.29	95.21	II	I	no	
NPDC046625	NPDC046888	97.24	79.55	77.84	96.24	II	V	no	67.8
NPDC046625	NPDC047015	97.16	79.68	80.82	96.25	II	V	no	67.8
NPDC046625	NPDC052111	96.29	75.70	76.12	95.29	II	VI	no	60.0
NPDC046625	NPDC057006	96.03	75.07	72.26	94.81	II	III	no	57.1
NPDC046625	NPDC088830	96.33	75.31	76.25	95.18	II	I	no	
NPDC046625	NPDC101287	96.03	74.42	71.86	94.77	II	III	no	57.2
NPDC046625	NPDC127097	95.98	74.15	73.95	94.81	II	III	no	57.1
NPDC046625	TP-A0598	96.36	75.18	76.91	95.18	II	I	no	
NPDC046888	NPDC047015	99.67	90.47	93.79	99.38	V	V	yes	95.3
NPDC046888	NPDC052111	96.08	75.54	77.63	95.10	V	VI	no	57.8
NPDC046888	NPDC057006	95.68	74.40	73.19	94.69	V	III	no	55.7
NPDC046888	NPDC088830	95.93	74.75	77.34	94.91	V	I	no	
NPDC046888	NPDC101287	95.84	75.16	74.17	94.74	V	III	no	56.3
NPDC046888	NPDC127097	95.83	73.47	74.88	94.77	V	III	no	56.2
NPDC046888	TP-A0598	95.91	73.84	77.20	94.93	V	I	no	
NPDC047015	NPDC052111	96.03	78.63	77.95	95.07	V	VI	no	57.7
NPDC047015	NPDC057006	95.75	76.95	73.02	94.76	V	III	no	55.9
NPDC047015	NPDC088830	95.93	77.17	77.02	95.02	V	I	no	
NPDC047015	NPDC101287	95.85	77.43	73.71	94.79	V	III	no	56.3
NPDC047015	NPDC127097	95.78	75.49	74.23	94.78	V	III	no	56.2
NPDC047015	TP-A0598	95.90	75.88	76.53	95.02	V	I	no	
NPDC052111	NPDC057006	95.81	75.67	72.44	94.76	VI	III	no	55.3
NPDC052111	NPDC088830	96.09	76.75	77.27	95.03	VI	I	no	
NPDC052111	NPDC101287	95.78	74.96	71.99	94.63	VI	III	no	55.3
NPDC052111	NPDC127097	95.70	74.08	73.48	94.76	VI	III	no	55.6
NPDC052111	TP-A0598	96.06	76.36	77.69	95.13	VI	I	no	
NPDC057006	NPDC088830	96.27	76.56	80.53	95.35	III	I	no	59.3
NPDC057006	NPDC101287	98.81	89.83	90.12	98.24	III	III	yes	83.5
NPDC057006	NPDC127097	98.87	87.81	90.98	98.41	III	III	yes	84.7
NPDC057006	TP-A0598	96.29	76.73	81.55	95.34	III	I	no	59.4
NPDC088830	NPDC101287	96.36	79.88	76.19	95.24	I	III	no	59.2
NPDC088830	NPDC127097	96.28	79.40	78.22	95.37	I	III	no	59.4

NPDC088830	TP-A0598	99.64	93.63	94.61	99.43	I	I	yes	94.9
NPDC101287	NPDC127097	98.80	87.51	90.38	98.29	III	III	yes	83.6
NPDC101287	TP-A0598	96.41	75.67	80.16	95.25	III	I	no	59.1
NPDC127097	TP-A0598	96.28	77.62	79.62	95.34	III	I	no	59.5

Table S8: Genome panels used in this work.

**Panel**	**Genomes**	**Purpose**
Genus scan	13,198: 13,172 unique GenBank/RefSeq assemblies of Streptomyces plus the genomes of the NPDC collection that carry no INSDC accession	Frequency of carriage of the oxytetracycline locus across the genus (Table 1, Table S5)
Species panel	25 strains of S. lydicamycinicus: bja295, 12 GenBank assemblies and 22 carriers of the NPDC collection, ten of which duplicate GenBank strains	Carriage within the species; pangenome statistics (Table S6)
De-replicated species panel	19 representatives, 20 with bja295, collapsed at 99.9% ANI	Pangenome statistics controlled for clonal redundancy
Complex panel	46 genomes of the S. lydicamycinicus complex	Genomospecies delimitation by dDDH and ANI (Tables S3, S7)
Carrier panel	42 de-duplicated carrier profiles	Cluster phylogeny and its comparison with the genome topology (Section 2.7)
Core-genome panel	325 genomes: 42 carriers and 283 non-carriers, 138 single-copy markers	Core-genome phylogeny, ancestral state reconstruction, cophylogenetic statistics
Species-level panel	66 genomes, 17 of them carriers	Ancestral state reconstruction at species level

Table S9: Composition of the culture media used in this work. Amounts are given per liter of medium. The pH is that specified by the source cited in the last column; where the source gives a range, the range is reproduced. V5 and the medium referred to as CSPY-ME in Section 3.6 are the same formulation.

**Medium**	**Composition (per litre)**	**Ref.**
ISP2 (yeast extract–malt extract agar)	Yeast extract 4.0 g, malt extract 10.0 g, glucose 4.0 g, agar 20.0 g, (pH 7.3)	[66]
ISP3 (oatmeal agar)	Oatmeal 20.0 g (cooked and filtered), trace salts solution 1.0 mL, agar 18.0 g, (pH 7.2)	[66]
ISP4 (inorganic salts–starch agar)	Soluble starch 10.0 g, $(NH_4)_2SO_4$ 2.0 g, K_2HPO_4 1.0 g, $MgSO_4{\cdot}7H_2O$ 1.0 g, NaCl 1.0 g, $CaCO_3$ 2.0 g, trace salts solution 1.0 mL, agar 20.0 g, (pH 7.2)	[66]
ISP5 (glycerol–asparagine agar)	L-Asparagine 1.0 g, glycerol 10.0 g, K_2HPO_4 1.0 g, trace salts solution 1.0 mL, agar 20.0 g, (pH 7.2)	[66]
ISP6 (peptone–yeast extract iron agar)	Peptone 15.0 g, proteose peptone 5.0 g, yeast extract 1.0 g, K_2HPO_4 1.0 g, ferric ammonium citrate 0.5 g, sodium thiosulfate 0.08 g, agar 15.0 g, (pH 7.2)	[66]
ISP9 (carbon utilisation basal medium)	$(NH_4)_2SO_4$ 2.64 g, KH_2PO_4 2.38 g, $K_2HPO_4{\cdot}3H_2O$ 5.65 g, $MgSO_4{\cdot}7H_2O$ 1.00 g, Pridham–Gottlieb trace salts solution 1.0 mL, agar 15.0 g, plus the carbon substrate under test at 1% (w/v), added aseptically after sterilization, (pH 7.0)	[66]
V1	Maltose 10 g, beef extract 5 g, peptone 5 g, NaCl 5 g, agar 20 g, (pH 7.0)	[67]
V2	Glucose 10 g, beef extract 5 g, peptone 5 g, NaCl 3 g, agar 20 g, (pH 7.0)	[67]
V3	Malt extract 15 g, yeast extract 5 g, soluble starch 5 g, agar 20 g, (pH 7.5)	[66]
V4 (Gause's medium No. 1)	Soluble starch 20 g, KNO_3 1.0 g, K_2HPO_4 0.5 g, $MgSO_4$ 0.5 g, NaCl 0.5 g, $FeSO_4$ 0.01 g, agar 20 g , (pH 7.4)	[68]
V5 (CSPY-ME)	Maltose 10 g, yeast extract 1.0 g, peptone 1.0 g, casein 0.3 g, K_2HPO_4 0.5 g, agar 20 g, (pH 7.0)	[69]

[a] The source specifies that the pH is not to be adjusted if it already lies within the stated range. [b] The same source states that the pH of the medium after sterilisation and solidification is about 7.4. [c] Reference [68] does not state a pH for this medium; the value given is that of the corresponding curated formulation of Gause's synthetic medium No. 1 (DSMZ Medium 1048).

Table S10: Elution programme of the preparative reversed-phase HPLC separation described in Section 3.6 (Agilent 1260 with diode-array detection and automated fraction collection; Luna C18(2), 5 µm, 100 Å, 250 ×

4.6 mm). Solvent A, water; solvent B, acetonitrile. Segments are linear. The active compound eluted as a single peak at 11.59 min, with an absorption maximum at 356 nm.

**Time (min)**	**Solvent A, water (%)**	**Solvent B, acetonitrile (%)**	**Step**
0	100	0	Start
2	90	10	Linear ramp
10	66	34	Linear ramp
14	0	100	Linear ramp
16	0	100	Hold; end of run

Table S11: Component-by-component comparison of the culture media of defined composition used in this work, with the pH of each medium and the production of oxytetracycline (OTC) on it. Rows are media, columns are individual components; each cell gives the amount of that component in grams per litre of medium, or a dash where the component is absent. ISP3 (oatmeal agar) is not included, because oatmeal is an undefined raw material whose components cannot be listed individually.

**Medium**	**Glucose**	**Maltose**	**Soluble starch**	**Glycerol**	**Carbon substrate under test**	**Malt extract**	**Yeast extract**	**Peptone**	**Proteose peptone**	**Beef extract**	**Casein**	**L-Asparagine**	**$(NH_4)_2SO_4$**	**KNO_3**	**K_2HPO_4**	**KH_2PO_4**	**$MgSO_4$**	**NaCl**	**$CaCO_3$**	**$FeSO_4$**	**Ferric ammonium citrate**	**Sodium thiosulfate**	**Trace salts solution (mL)**	**Agar**	**OTC production**	**Ref.**
**ISP2**	**4.0**	**–**	**–**	**–**	**–**	**10.0**	**4.0**	**–**	**–**	**–**	**–**	**–**	**–**	**–**	**–**	**–**	**–**	**–**	**–**	**–**	**–**	**–**	**–**	**20.0**	**+++**	**[66]**
ISP4	–	–	10.0	–	–	–	–	–	–	–	–	–	2.0	–	1.0	–	1.0	1.0	2.0	–	–	–	1.0	20.0	++	[66]
ISP5	–	–	–	10.0	–	–	–	–	–	–	–	1.0	–	–	1.0	–	–	–	–	–	–	–	1.0	20.0	++	[66]
ISP6	–	–	–	–	–	–	1.0	15.0	5.0	–	–	–	–	–	1.0	–	–	–	–	–	0.5	0.08	–	15.0	+	[66]
ISP9	–	–	–	–	10.0	–	–	–	–	–	–	–	2.64	–	5.65	2.38	1.00	–	–	–	–	–	1.0	15.0	-	[66]
V1	–	10	–	–	–	–	–	5.0	–	5.0	–	–	–	–	–	–	–	5.0	–	–	–	–	–	20	-	[67]
V2	10	–	–	–	–	–	–	5.0	–	5.0	–	–	–	–	–	–	–	3.0	–	–	–	–	–	20	-	[67]
V3	–	–	5.0	–	–	15	5.0	–	–	–	–	–	–	–	–	–	–	–	–	–	–	–	–	20	-	[66]
V4 (Gause's No. 1)	–	–	20	–	–	–	–	–	–	–	–	–	–	1.0	0.5	–	0.5	0.5	–	0.01	–	–	–	20	-	[68]
V5 (CSPY-ME)	–	10	–	–	–	–	1.0	1.0	–	–	0.3	–	–	–	0.5	–	–	–	–	–	–	–	–	20	-	[69]

Amounts are in g L^{-1} except for the trace salts solution, which is given in mL L^{-1}. K_2HPO_4 is the anhydrous salt in every medium except ISP9, where the source specifies $K_2HPO_4 \cdot 3H_2O$. $MgSO_4$ and $FeSO_4$ are the heptahydrates in ISP4, ISP5 and ISP9; the source of V4 does not specify a hydration state. The trace salts solution of ISP4 and ISP5 contains $FeSO_4 \cdot 7H_2O$, $MnCl_2 \cdot 4H_2O$ and $ZnSO_4 \cdot 7H_2O$ at 0.1 g per 100 mL each; that of ISP9 is the Pridham–Gottlieb solution ($CuSO_4 \cdot 5H_2O$ 0.64 g, $FeSO_4 \cdot 7H_2O$ 0.11 g, $MnCl_2 \cdot 4H_2O$ 0.79 g, $ZnSO_4 \cdot 7H_2O$ 0.15 g per 100 mL). In ISP9 the carbon substrate under test is added aseptically after sterilisation to a final concentration of 1% (w/v).

OTC production refers to growth on the solid medium. Activity was followed with the Katushka2S reporter assay and quantified against a pharmacopoeial standard (Sections 2.1 and 2.2). ISP2 was the most productive of the media tested and is the only medium for which a titre was determined; on ISP4, ISP5 and ISP6 the antibiotic was detected at a lower level and was not quantified. Activity was also detected on ISP3, which is not shown in this table for the reason given in the legend. The V media were used for clonal resolution, and no production was detected on them as solid media; V3 and V5 were subsequently used for submerged cultivation, in which the compound was produced and isolated (Section 3.6).
